# Selective and Long‐Term Stable Ammonia Electrolysis Using Pt–WO_x_ Catalysts with Suppressed NO_x_ Formation and Enhanced Activity

**DOI:** 10.1002/advs.202515944

**Published:** 2025-10-27

**Authors:** Changhyun Lim, Hyogyun Roh, Hyeon Kim, Dayoung Kwon, Okkyun Seo, Jiayi Tang, Takeshi Watanabe, Wooyul Kim, Donghwa Lee, Kijung Yong

**Affiliations:** ^1^ Surface Chemistry Laboratory of Electronic Materials (SCHEMA) Department of Chemical Engineering Pohang University of Science and Technology (POSTECH) Pohang 37673 Republic of Korea; ^2^ Research Center for Carbon‐zero Green Ammonia Cycling Pohang University of Science and Technology (POSTECH) Pohang 37673 Republic of Korea; ^3^ Department of Materials Science and Engineering (MSE) Pohang University of Science and Technology (POSTECH) Pohang 37673 Republic of Korea; ^4^ Division of Advanced Materials Science (AMS) Pohang University of Science and Technology (POSTECH) Pohang 37673 Republic of Korea; ^5^ Institute for Convergence Research and Education in Advanced Technology (I_CREATE) Yonsei University Incheon 21983 Republic of Korea; ^6^ Department of Energy Engineering Korea Institute of Energy Technology (KENTECH) Naju 58217 Republic of Korea; ^7^ Japan Synchrotron Radiation Research Institute (JASRI) Kouto, Sayo‐cho Sayo‐gun Hyogo 679–5198 Japan

**Keywords:** ammonia electrolysis, ammonia oxidation reaction, hydrogen evolution reaction, metal–support interaction, photodeposition

## Abstract

Ammonia electrolysis presents a promising strategy for low‐voltage hydrogen production; however, its advancement is impeded by limitations in electrocatalyst performance due to sluggish reaction kinetics and deactivation caused by strongly adsorbed nitrogen‐containing intermediates. In this study, a photodeposited Pt on tungsten suboxide (WO_x_) nanowires (Pt–WO_x_ (P)) is introduced as a bifunctional electrocatalyst for ammonia electrolysis. The photodeposition induces strong metal–support interaction, resulting in robust interfacial bonding between Pt and WO_x_ and facilitating electron transfer from WO_x_ to Pt. ATR‐SEIRAS (attenuated total reflection‐surface enhanced infrared absorption spectroscopy) identifies the Gerischer–Mauerer (G–M) mechanism through absorbance peaks corresponding to NH_2_ and N_x_H_y_ (x = 1–2) and reveals effective suppression of NO_x_ poisoning on Pt–WO_x_ (P). DFT confirms the electronic modulation of Pt significantly lowers the energy barrier of the rate‐determining step (RDS) for the conversion of *NH_2_ to *NH by enhancing hydrogen bonding of *NH and *OH. As a result, Pt–WO_x_ (P) exhibits outstanding AOR activity, achieving a high peak current density of 49.69 mA cm^−2^. Furthermore, it demonstrates remarkable stability for over 120 h during ammonia electrolysis. In an MEA‐based flow cell, Pt–WO_x_ (P) delivers current densities exceeding 500 mA cm^−2^, underscoring its potential as a bifunctional catalyst for ammonia electrolysis applications.

## Graphical abstract

This study reports photodeposited Pt nanoparticles on WO_x_ nanowires (Pt–WO_x_ (P)) as a bifunctional catalyst for ammonia electrolysis. Strong metal–support interactions promote electron transfer to Pt, lowering the energy barrier of the rate‐determining step and suppressing NO_x_ formation. The catalyst achieves 500 mA cm^−2^ current density and 120 h durability, advancing sustainable hydrogen production from green ammonia.

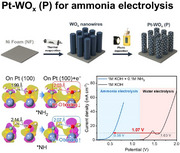

## 1 Introduction

Ammonia (NH_3_) has emerged as a promising hydrogen carrier due to its high hydrogen content (17.8 wt.%), substantial volumetric energy density (11.3 MJ L^−1^), and ease of liquefaction under mild conditions (20 °C and 9.2 bar).^[^ ^1^, ^2^, ^3^ ^]^ It also benefits from well‐established infrastructure, making it suitable for safe and efficient storage and transport as well as serving as a key energy carrier within the nitrogen cycle.^[^ ^4^, ^5^, ^6^, ^7^, ^8^ ^]^ To enable hydrogen production from green ammonia, the ammonia oxidation reaction (AOR) is essential and can be conducted either thermally or electrochemically. Thermochemical decomposition requires high temperatures and pressures, inevitably resulting in significant greenhouse gas emissions.^[^ ^9^, ^10^, ^11^ ^]^ In contrast, electrochemical AOR, when coupled with the hydrogen evolution reaction (HER) in ammonia electrolysis cells (AECs), provides a more environmentally benign pathway.^[^ ^12^, ^13^, ^14^ ^]^ This process offers considerable energy savings due to its significantly lower theoretical cell voltage (0.06 V_cell_) compared to conventional water electrolysis (1.23 V_cell_).^[^ ^15^, ^16^ ^]^ Moreover, electrochemical AOR operates at low temperatures and produces only nitrogen and water as by‐products, rendering it a sustainable and carbon‐free alternative for hydrogen generation. (Equations (1), (2), (3)):^[^ ^17^ ^]^

(1) $$\begin{array}{cc} & \mathrm{Anode}\;\left(\mathrm{AOR}\right)\;2NH_{3}+6OH^{-}\to \\ & \;N_{2}+6H_{2}O+6e^{-}\left(E^{0}=-0.77\;V\;\mathrm{vers}\mathrm{us}\;\mathrm{SHE}\right) \end{array}$$

(2) $$\begin{array}{cc} & \mathrm{Cath}\mathrm{ode}\left(\mathrm{HER}\right)6H_{2}O+6e^{-}\to \\ & \;3H_{2}+6OH^{-}\left(E^{0}=-0.83\;V\;\mathrm{vers}\mathrm{us}\;\mathrm{SHE}\right) \end{array}$$

(3) $$\mathrm{Over}\mathrm{all}2NH_{3}\to N_{2}+3H_{2}\left(E^{0}=0.06\;V_{\mathrm{cell}}\right)$$

Despite its advantages, ammonia electrolysis faces significant challenges that hinder its widespread adoption. The sluggish kinetics of the six‐electron transfer process in the AOR lead to high overpotentials and limited current densities.^[^ ^18^ ^]^ Additionally, the excessive adsorption strength between nitrogen species and the catalyst surface leads to the formation of NO_x_ by‐products, which block active sites and ultimately trigger catalyst deactivation and poisoning.^[^ ^19^, ^20^ ^]^ The low power output per unit area further remains a critical obstacle to commercialization. Therefore, the development of advanced AOR electrocatalysts capable of accelerating reaction kinetics, suppressing nitrogen‐related surface poisoning, and delivering high areal power output is essential for realizing energy‐efficient, durable, and commercially viable ammonia electrolysis systems.

Platinum (Pt) is widely regarded as the most active catalyst for AOR due to its optimal adsorption strength for ammonia and nitrogen‐based intermediates.^[^ ^21^, ^22^, ^23^, ^24^ ^]^ To enhance AOR catalyst performance, several strategies have been explored, including facet engineering of Pt nanoparticles to expose highly active (100) surfaces,^[^ ^25^, ^26^ ^]^ and alloying Pt with various noble (Ir, Ru, and Pd) and non‐noble (Ni, Fe, Mo, and Co) metals to modulate its electronic structure.^[^ ^27^, ^28^, ^29^, ^30^, ^31^ ^]^ Among these, PtIr‐based electrocatalysts have exhibited notable activity at low overpotentials.^[^ ^32^ ^]^ However, their practical application is constrained by the high cost and scarcity of Ir, as well as its excessively strong binding affinity for nitrogen‐containing species, which often leads to increased formation of NO_x_ by‐products.^[^ ^33^ ^]^ Crucially, while substantial efforts have been directed toward improving either geometric or mass activity, achieving both high geometric current density and efficient noble metal utilization remains a major challenge. Many studies emphasize only one performance metric, complicating direct comparisons across systems.^[^ ^34^ ^]^ Moreover, the mechanistic understanding of AOR is still limited, particularly with respect to the identification of nitrogenous intermediates (*N_x_H_y_) and the suppression of nitrogenous by‐products (*NO_x_).^[^ ^35^, ^36^ ^]^ These limitations underscore the urgent need for rational catalyst design strategies that balance performance metrics while providing insight into selectivity control under practical electrochemical conditions.

In this study, we propose photodeposited Pt on WO_x_ nanowires (Pt–WO_x_ (P)) as a high‐performance electrocatalyst for the AOR. Although photodeposition is widely employed in photocatalysis,^[^ ^37^, ^38^ ^]^ its application in electrocatalyst design, particularly for AOR, remains largely unexplored. In contrast to conventional Pt synthesis methods, such as hydrothermal, polyol, or high‐temperature H_2_ reduction, which often involve harsh chemicals or elevated temperatures with potential environmental risks, the photodeposition approach operates under ambient conditions while utilizing methanol as a mild hole scavenger.^[^ ^39^ ^]^ Previous studies have commonly utilized photoactive, fully oxidized tungsten oxide (WO_3_) as a support for Pt photodeposition; however, these systems often suffer from heterogeneous particle distribution and poor morphological control.^[^ ^40^, ^41^ ^]^ In this work, we introduce tungsten suboxide (WO_x_ based on nonstoichiometric W_18_O_49_) as a novel support material, offering stronger metal–support interactions with Pt than WO_3_. This enhanced interfacial interaction promotes the formation of smaller and more uniformly dispersed Pt nanoparticles, while also facilitating electron transfer from WO_x_ to the 5d orbitals of Pt. F. Yang et al. emphasized that optimizing nanoparticle size is essential for enhancing mass activity and maximizing active site utilization.^[^ ^42^ ^]^ S. Barik et al. underscored the critical role of metal–support interactions in governing the stability of ammonia oxidation catalysts.^[^ ^43^ ^]^ Also, C. Wang et al. demonstrated that increasing the electronic density of Pt effectively suppresses nitrogen poisoning in ammonia oxidation.^[^ ^44^ ^]^ Drawing from these insights, we anticipate that Pt–WO_x_ (P) will exhibit superior AOR performance with respect to both activity and durability.

The Pt–WO_x_ (P) catalyst exhibits exceptional AOR activity, achieving a peak current density of 49.69 mA cm^−2^ and a mass activity of 79.97 A g^−1^_PGM_ in 1 m KOH with 0.1 m NH_3_, which demonstrates high intrinsic performance and efficient Pt utilization. In addition, Pt–WO_x_ (P) shows excellent hydrogen evolution activity, requiring only 26.0 mV of overpotential to reach 10 mA cm^−2^, thereby functioning as a highly efficient bifunctional catalyst for AECs. Building on the concept of water electrolysis and small molecule‐assisted electrolysis,^[^ ^45^, ^46^, ^47^, ^48^ ^]^ we established an ammonia electrolysis system that delivers a current density of 10 mA cm^−2^ at only 0.56 V, representing a 1.07 V reduction compared to conventional water electrolysis. Pt–WO_x_ (P) also exhibits remarkable operational stability, sustaining continuous operation for 120 h under a pulsed current protocol. When incorporated into a membrane electrode assembly (MEA)‐based flow cell with a 5 cm^2^ electrode area, the catalyst delivers current densities exceeding 500 mA cm^−2^, confirming its scalability and practical viability. To gain mechanistic insights into AOR, attenuated total reflectance surface‐enhanced infrared absorption spectroscopy (ATR‐SEIRAS) identified NH_2_ and N_x_H_y_ intermediates, confirming the Gerischer–Mauerer (G–M) pathway^[^ ^18^ ^]^ as the dominant reaction mechanism. Notably, the formation of NO and NO_2_ by‐products was effectively suppressed within the operating potential window, in contrast to commercial and control catalysts. DFT calculations revealed that nonstoichiometric WO_x_ forms stronger interfacial bonding with Pt than fully oxidized WO_3_, resulting in smaller and more uniformly dispersed Pt nanoparticles. Furthermore, the electron‐enriched Pt surface on WO_x_ support compared to WO_3_ support strengthens the hydrogen bonding between the H atom of *NH and the O atom of *OH, which stabilizes the *NH intermediate, thereby lowering the energy barrier of the RDS and enhancing the AOR catalytic activity.

## 2 Results and Discussion

### 2.1 AOR Electrocatalysts Synthesis

**Scheme 1:**
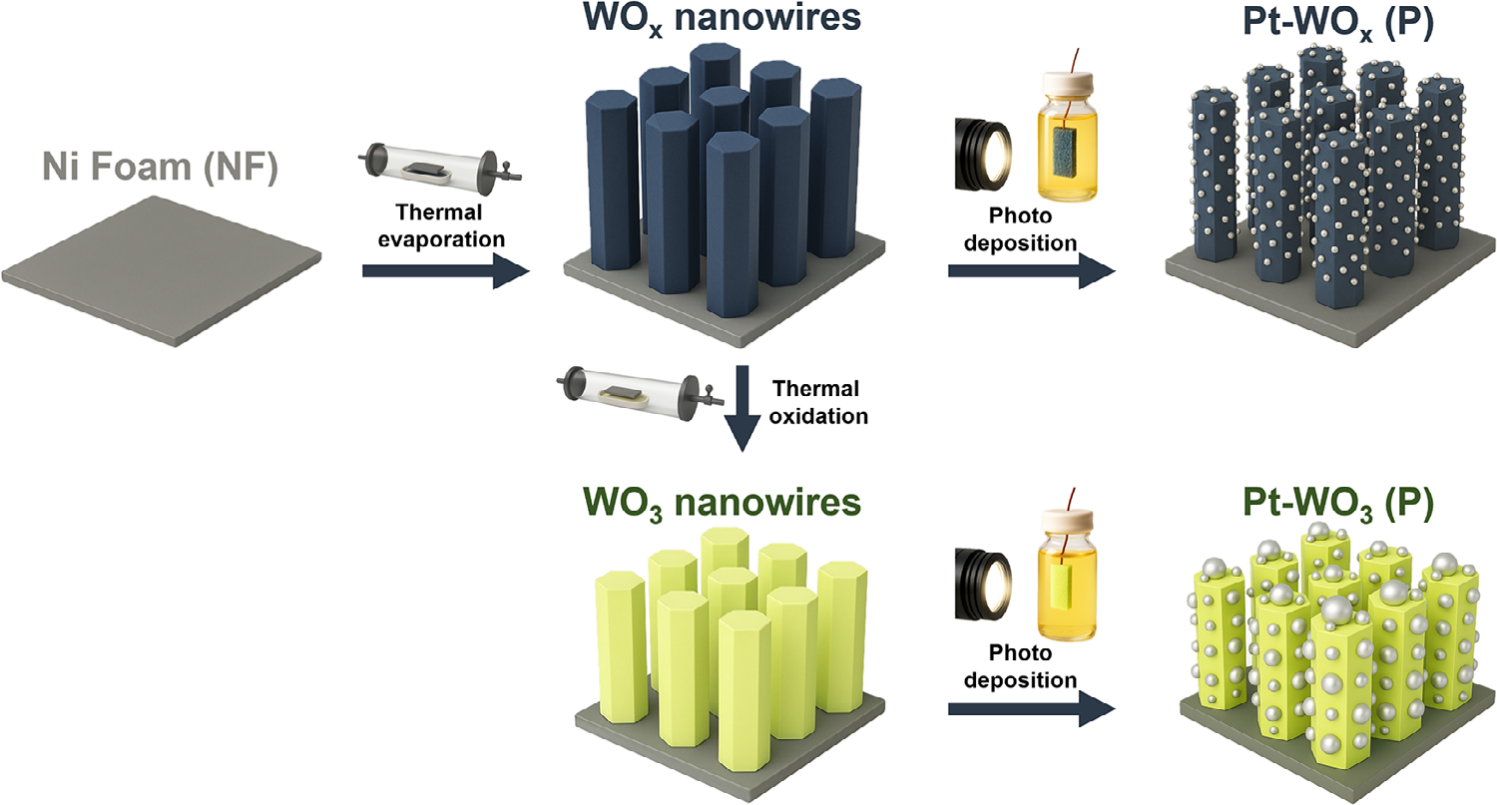
Schematic representation of the fabrication processes for Pt–WO_x_ (P) and Pt–WO_3_ (P).

**Scheme** **1** illustrates the synthetic procedures for Pt–WO_x_ (P) and Pt–WO_3_ (P). Pt–WO_x_ (P) was synthesized via a straightforward two‐step process involving thermal evaporation followed by photodeposition. WO_x_ nanowires (NWs) were directly grown on cleaned Ni foam using a vapor–solid growth mechanism,^[^ ^49^ ^]^ resulting in the formation of dark bluish WO_x_, as shown in Figure S1 (Supporting Information). The WO_x_ NWs on Ni foam were then immersed in an aqueous solution containing H_2_PtCl_6_, methanol, and deionized water, and irradiated under a 300 W Xe lamp (Newport, 66 902) with an intensity equivalent to one sun for 3 h. Leveraging the photocatalytic properties of the semiconductor WO_x_, Pt nanoparticles were strongly anchored onto the WO_x_ surface during the photodeposition process. WO_3_ NWs on Ni foam were obtained by thermochemical oxidation of the as‐grown WO_x_ nanowires, during which the dark bluish color changed to greenish‐yellow, as shown in Figure S2 (Supporting Information). Using the same photodeposition method, Pt–WO_3_ (P) was subsequently synthesized. For comparison, Pt–WO_x_ (S) was prepared by sputtering Pt onto WO_x_ NWs, while commercial PtIr/C and Pt/C catalysts were drop‐cast onto Ni foam.

### 2.2 Characterization of Pt–WO_x_ (P)

**Figure 1:**
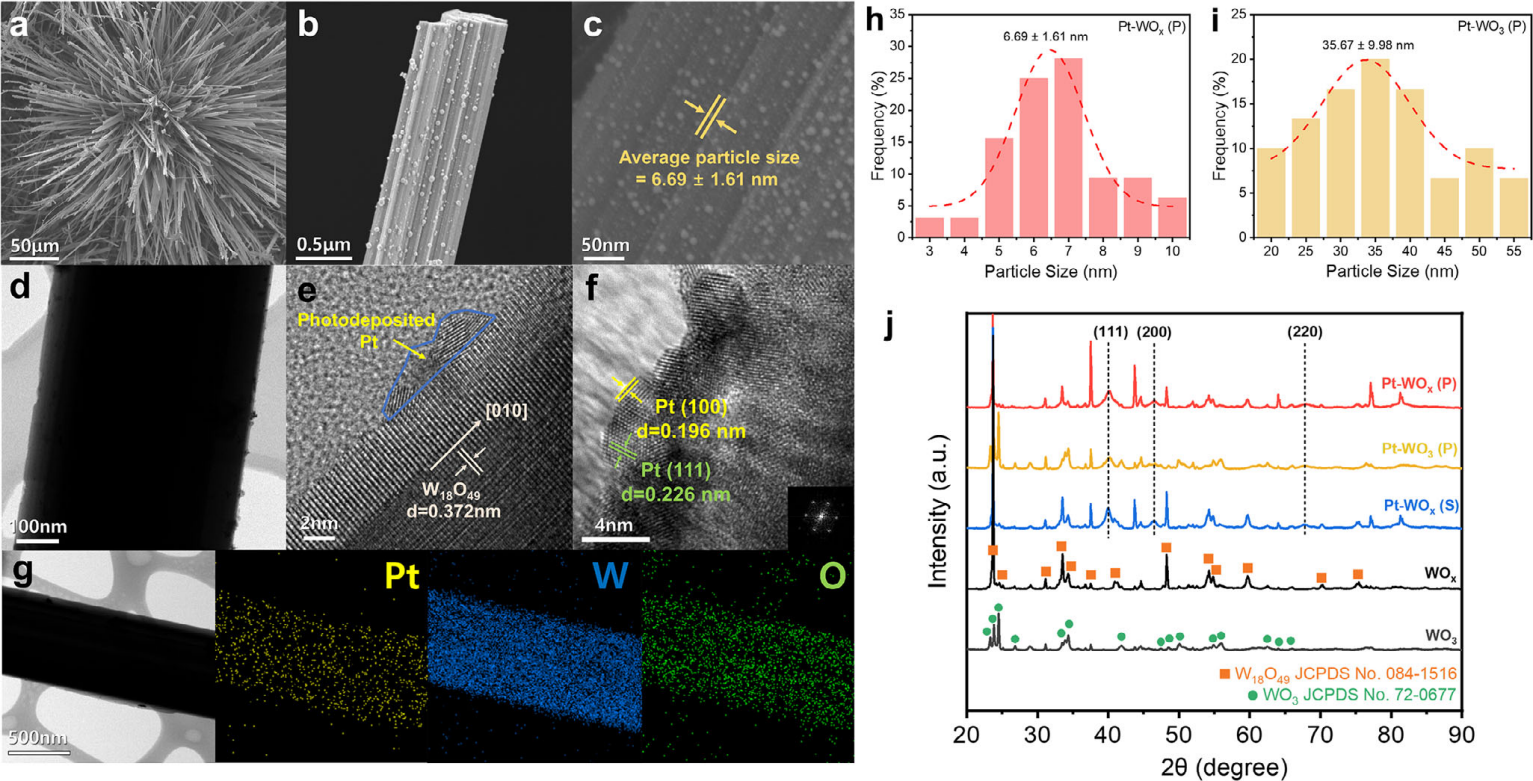
a,b) Low‐magnification and c) high‐magnification FESEM images of Pt–WO_x_ (P). d) Low‐resolution TEM image of Pt–WO_x_ (P). High‐resolution TEM images of Pt–WO_x_ (P) showing the crystallinity of e) W_18_O_49_ and f) Pt (insets: corresponding FFT patterns). g) TEM image and corresponding EDS elemental mappings of Pt, W, and O. h) Particle size distribution of Pt–WO_x_ (P); i) particle size distribution of Pt–WO_3_ (P). j) XRD patterns of Pt–WO_x_ (P), Pt–WO_3_ (P), Pt–WO_x_ (S), WO_x_, and WO_3_.

The morphology and structure of Pt–WO_x_ (P), Pt–WO_3_ (P), and Pt–WO_x_ (S) were analyzed by field emission scanning electron microscopy (FESEM). In Pt–WO_x_ (P) (**Figure** **1**a; Figure S3, Supporting Information) and Pt–WO_x_ (S) (Figure S5, Supporting Information), as well as Pt–WO_3_ (P) (Figure S4, Supporting Information), the WO_x_ and WO_3_ NWs are vertically grown on Ni foam, forming a uniform urchin‐like architecture. These NWs are morphologically consistent with bare WO_x_ and WO_3_ NWs without Pt deposition (Figures S6 and S7, Supporting Information). The intrinsic 1D growth behavior of tungsten oxide facilitates the formation of long, smooth nanowires during thermal evaporation. This urchin‐like morphology enhances light absorption through multiple internal reflections, which minimize shadowing effects and promote uniform photon distribution across the surface.^[^ ^50^, ^51^ ^]^ A high‐magnification FESEM image of Pt–WO_x_ (P) (Figure 1b; Figure S3, Supporting Information) reveals individual WO_x_ NWs decorated with uniformly distributed Pt nanoparticles. The average particle size of Pt–WO_x_ (P) is 6.69 ± 1.61 nm (Figure 1c,h), suggesting well‐controlled photodeposition. In contrast, Pt–WO_3_ (P) exhibits non‐uniform Pt nanoparticle distribution and significant size variation, as shown in Figure S4b (Supporting Information), with a considerably larger average particle size of 35.67 ± 9.98 nm (Figure 1i; Figure S4, Supporting Information). This broader size distribution indicates uncontrolled nucleation and agglomeration on the WO_3_ surface, likely resulting from its weaker interaction with Pt atoms. For Pt–WO_x_ (S), where Pt was deposited via sputtering, a distinctly different morphology is observed: Pt forms a continuous thin film that uniformly covers the WO_x_ surface, rather than discrete nanoparticles (Figure S5, Supporting Information).

WO_x_ NWs and surface‐deposited Pt nanoparticles are clearly discernible in Figure 1d. High‐resolution transmission electron microscopy (TEM) reveals lattice fringes with a d‐spacing of 0.372 nm, corresponding to the (010) plane of W_18_O_49_, indicating a preferential growth orientation (Figure 1e). These findings are consistent with the d‐spacing and fast Fourier transform (FFT) pattern observed for bare WO_x_ in Figure S8a (Supporting Information). Lattice spacings of 0.196 and 0.226 nm, corresponding to the (100) and (111) facets of metallic Pt, respectively, indicate the coexistence of both facets on the surface (Figure 1f). The pronounced exposure of the Pt (100) facet is particularly significant for the AOR, as it provides balanced adsorption energies for NH_2_ and NH intermediates, thereby facilitating both dehydrogenation and N–N dimerization steps.^[^ ^52^ ^]^ The FFT pattern shown in the inset of Figure 1f further confirms the presence of these planes through corresponding diffraction spots. Energy‐dispersive X‐ray spectroscopy (EDS) elemental mapping (Figure 1g) demonstrates a homogeneous spatial distribution of Pt, W, and O atoms across the NW surface in Pt–WO_x_ (P). In contrast, TEM analysis of Pt–WO_3_ (P) reveals irregular Pt distribution and the formation of larger aggregates on the WO_3_ surface (Figure S9, Supporting Information). Pt–WO_3_ (P) exhibits distinct lattice fringes with a spacing of 0.362 nm, attributed to the (200) plane of monoclinic WO_3_ (Figure S10a, Supporting Information), consistent with the fringes observed for bare WO_3_ in Figure S8b (Supporting Information). Although EDS elemental mapping confirms uniform distributions of W and O along the wire backbone, the Pt signal displays noticeable local variations in intensity (Figure S10b, Supporting Information). In Pt–WO_x_ (S), the Pt phase forms a continuous coating along the NW edges (Figure S11, Supporting Information). The dominant exposed facet is identified as Pt (111), with a lattice spacing of 0.226 nm (Figure S12a, Supporting Information). Elemental mapping (Figure S12b, Supporting Information) reveals that Pt forms a conformal thin‐film morphology, distinct from the nanoparticle‐based architecture observed in the photodeposited samples.

Pt–WO_x_ (P), Pt–WO_3_ (P), and Pt–WO_x_ (S) exhibit XRD reflections at 39.8^○^, 46.2^○^, and 67.5^○^, corresponding to the (111), (200), and (220) planes of face‐centered cubic Pt, respectively, consistent with the standard JCPDS pattern No. 04–0802 (Figure 1j). Among the samples, Pt–WO_x_ (P) displays the most intense and well‐resolved Pt peaks, indicating high crystallinity and uniform nanoparticle deposition. The WO_x_‐ and WO_3_‐based supports also exhibit distinct phase signatures. Both Pt–WO_x_ (P) and Pt–WO_x_ (S) show diffraction peaks matching monoclinic W_18_O_49_ (JCPDS No. 084–1516), including a prominent peak at 23.7^○^ (d = 0.375 nm). In contrast, Pt–WO_3_ (P) exhibits a strong reflection at 24.5^○^ (d = 0.363 nm), corresponding to the (200) plane of crystalline WO_3_ (JCPDS No. 72–0677). These results are consistent with the HRTEM analysis and confirm that Pt deposition does not disrupt the underlying crystal structure of the supports.

To investigate the intrinsic physicochemical properties of the catalysts, X‐ray absorption spectroscopy (XAS), including X‐ray absorption near‐edge structure (XANES) and extended X‐ray absorption fine structure (EXAFS), was employed. These analyses provide a detailed understanding of the oxidation states of Pt and W, elucidating the electronic and structural characteristics that govern catalytic behavior. To probe the electronic structure and oxidation state of Pt, the Pt L_2_‐edge (2p_1/2_ → 5d) was utilized instead of the L_3_‐edge (2p_3/2_ → 5d), due to spectral overlap with the W L_3_‐edges. Moreover, accurate EXAFS analysis requires a sufficiently long post‐edge region (≈1000 eV beyond the absorption edge), which is not available at the Pt L_3_‐edge.^[^ ^53^, ^54^ ^]^

**Figure 2:**
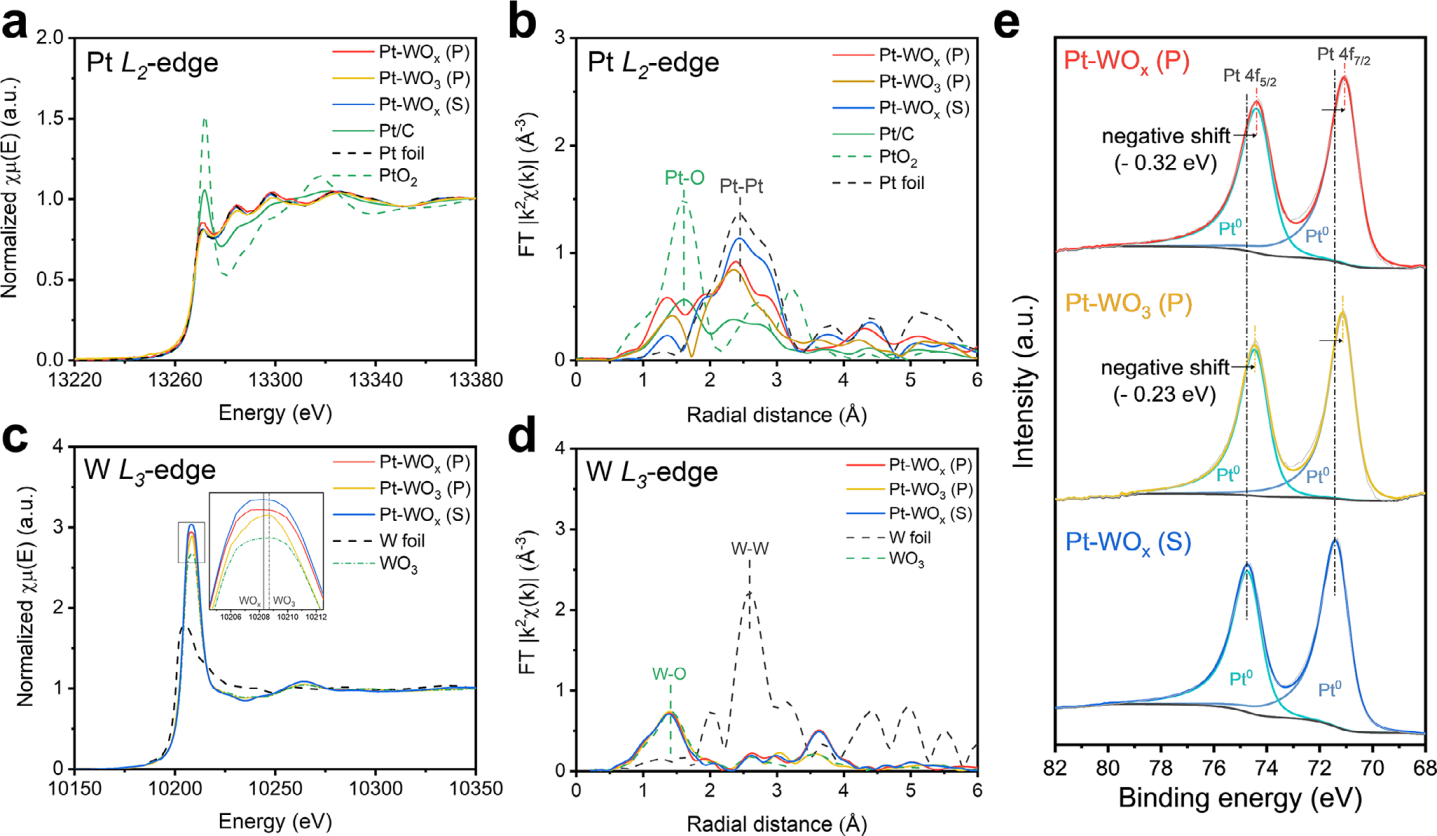
a) Normalized XANES spectra and b) FT‐EXAFS spectra at the Pt L_2_‐edge from Pt–WO_x_ (P), Pt–WO_3_ (P), Pt–WO_x_ (S), Pt/C, and reference samples of PtO_2_, and Pt foil. c) Normalized XANES spectra and d) FT‐EXAFS spectra at the W L_3_‐edge from Pt–WO_x_ (P), Pt–WO_3_ (P), Pt–WO_x_ (S), reference samples of W foil and WO_3_. e) XPS spectra of Pt 4f from Pt–WO_x_ (P), Pt–WO_3_ (P), and Pt–WO_x_ (S).

Normalized XANES spectra of Pt–WO_x_ (P), Pt–WO_3_ (P), and Pt–WO_x_ (S) exhibit white line intensities and edge positions closely resembling those of Pt foil, indicating the presence of predominantly metallic Pt species (**Figure** **2**a). Both photodeposited and sputtered samples show characteristic features of metallic Pt. In the case of photodeposition, the use of methanol as a hole scavenger likely facilitated the formation of electron‐rich Pt nanoparticles. For the sputtered sample, metallic Pt atoms ejected from the Pt target under Ar⁺ ion bombardment were physically deposited onto the WO_x_ surface in vacuum, without exposure to oxidizing species, thereby preserving their metallic state. In contrast, the commercial Pt/C catalyst exhibits mixed oxidation states, as evidenced by the higher white line intensity, indicating the presence of oxidized Pt species. Similarly, the PtIr/C reference catalyst displays spectral features consistent with the coexistence of Pt oxides, as shown in Figure S13a (Supporting Information). EXAFS analysis at the Pt L_2_‐edge (Figure 2b) reveals pronounced Pt–Pt coordination peaks for Pt–WO_x_ (P), Pt–WO_3_ (P), and Pt–WO_x_ (S), consistent with the structure of face‐centered cubic metallic Pt.^[^ ^55^ ^]^ Weak Pt–O coordination peaks of Pt–WO_x_ (P) are also observed, appearing at slightly shorter radial distances than those in PtO_2_ or Pt/C, suggesting compressed Pt─O bonding due to strong interactions with the tungsten suboxide support.^[^ ^56^ ^]^ Notably, the photodeposited Pt nanoparticles in Pt–WO_x_ (P), with an average size of 6–7 nm, likely undergo significant lattice distortion induced by the support, resulting in the most intense Pt─O coordination peak. For commercial catalysts, Pt–Pt coordination is scarcely observed in both Pt/C (Figure 2b) and PtIr/C (Figure S13b, Supporting Information), while Pt─O coordination appears with significantly higher intensity.

The oxidation state of tungsten was investigated using W L_3_‐edge XANES analysis, with particular attention to the edge shift of the spectrum peak (Figure 2c). Metallic W (W^0^) exhibited a characteristic absorption peak at 10 204.1 eV, whereas fully oxidized WO_3_ (W^6+^) showed a peak at 10 208.9 eV.^[^ ^57^, ^58^ ^]^ The spectrum of Pt–WO_3_ (P) closely matched that of the WO_3_ reference, confirming a fully oxidized W^6+^ state. In contrast, Pt–WO_x_ (P) and Pt–WO_x_ (S) exhibited edge positions shifted 0.6 eV lower, at 10 208.3 eV, consistent with partially reduced W species. This subtle downshift reflects the non‐stoichiometric nature of WO_x_, where some tungsten atoms retain more electrons due to oxygen deficiency, resulting in absorption at slightly lower energies.^[^ ^59^ ^]^ EXAFS analysis of the W coordination environment (Figure 2d) revealed that all catalysts maintain W–O radial distances comparable to those in the WO_3_ reference, indicating that the WO_x_ and WO_3_ lattice frameworks remained structurally intact following Pt deposition.

XPS analysis of the Pt 4f region further confirmed the metallic nature of Pt in all samples (Figure 2e). In Pt–WO_x_ (S), the Pt^0^ peaks appeared at 71.33 eV (4f_7/2_) and 74.66 eV (4f_5/2_), indicating the exclusive presence of metallic Pt.^[^ ^60^, ^61^ ^]^ For Pt–WO_x_ (P), the corresponding peaks were observed at 71.00 and 74.32 eV, representing a negative shift of 0.32 eV compared to the sputtered sample. This shift suggests electron transfer from WO_x_ to Pt, indicative of a metal–support interaction induced by the photodeposition process.^[^ ^62^ ^]^ Theoretically, such charge transfer is favored due to the lower work function of W_18_O_49_ (≈4.55 eV)^[^ ^63^, ^64^ ^]^ relative to Pt (≈5.12–5.93 eV),^[^ ^65^, ^66^ ^]^ which facilitates electron donation from the support to the metal and promotes interfacial bonding. Pt–WO_3_ (P) exhibited Pt^0^ peaks at 71.08 and 74.40 eV, corresponding to a moderate negative shift of ≈0.24 eV, less pronounced than that observed for Pt–WO_x_ (P). Finally, W 4f XPS analysis (Figure S14, Supporting Information) was used to distinguish between WO_x_ and WO_3_ supports. The W^6+^ to W^5+^ ratio in Pt–WO_x_ (P) was 75.4:24.6, compared to 84.5:15.5 in Pt–WO_3_ (P), confirming a higher content of W^5+^ in the WO_x_‐based catalyst. This elevated degree of oxygen deficiency supports the hypothesis of electronic modulation at the Pt–WO_x_ interface, where enhanced metal–support interactions contribute to improved AOR stability.

### 2.3 Electrocatalytic Performances of Catalysts in AOR

**Figure 3:**
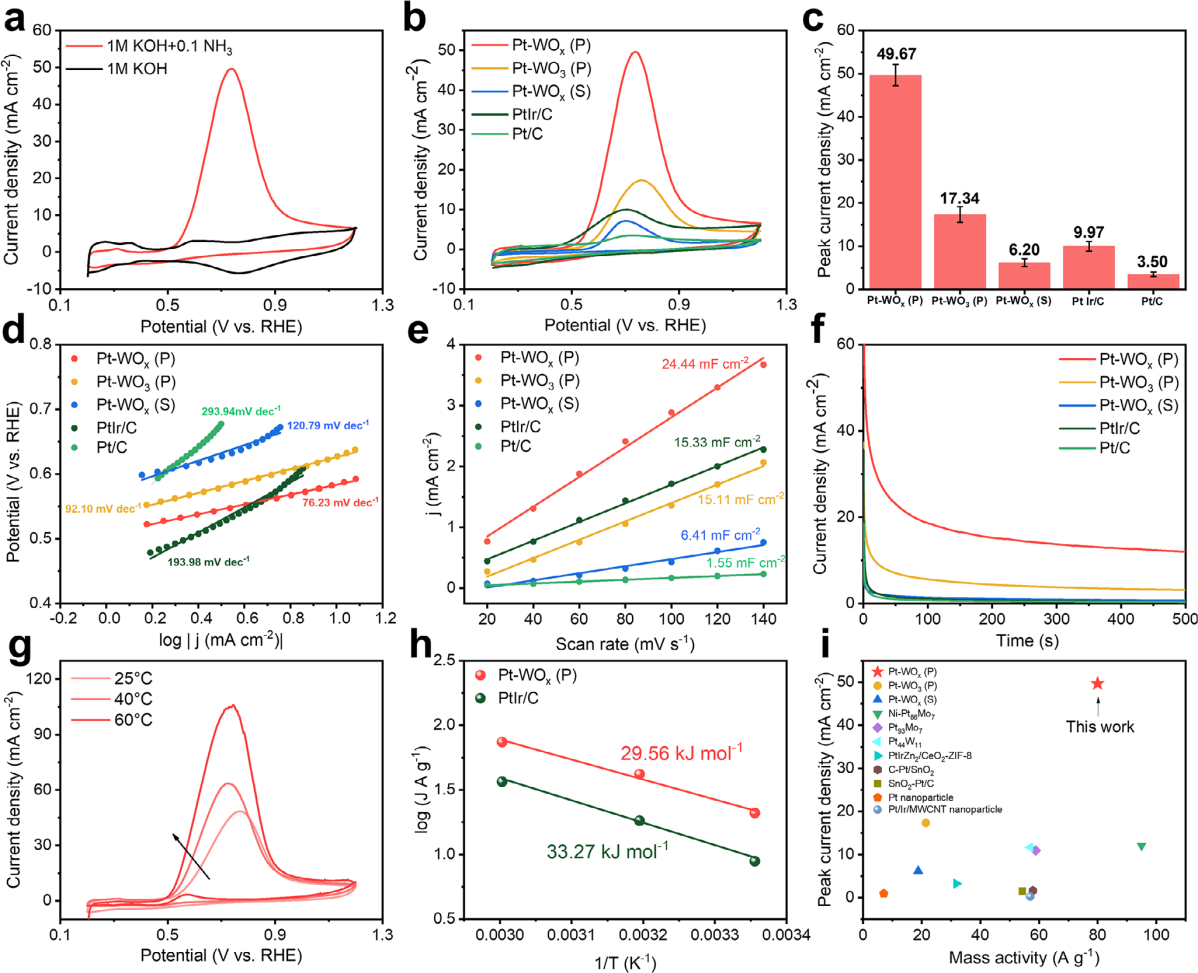
a) CV curves of Pt–WO_x_ (P) in 1 m KOH with and without 0.1 m NH_3_. b) CV curves of various catalysts in Ar‐saturated 1.0 m KOH + 0.1 m NH_3_ at a scan rate of 5 mV s^−1^ without iR correction. c) Comparison of peak current densities, d) Tafel plots, and e) electrochemical double‐layer capacitance (C_dl_) for different catalysts. f) Chronoamperometry (CA) profiles over 500 s in Ar‐saturated 1.0 m KOH + 0.1 m NH_3_. g) CV curves at different temperatures and h) corresponding Arrhenius plots for ammonia oxidation on Pt–WO_x_ (P) and commercial PtIr/C. i) Comparison of ammonia oxidation performance with catalysts reported in previous studies.

Electrocatalytic activity toward ammonia oxidation was evaluated using cyclic voltammetry (CV) in an electrolyte containing 1.0 m KOH and 0.1 m NH_3_ at room temperature, with a scan rate of 5 mV s^−1^. All AOR measurements are reported without iR compensation. As shown in **Figure** **3**a, the CV curve of Pt–WO_x_ (P) exhibits a distinct oxidation peak between 0.4 and 0.9 V versus RHE, which appears only in the presence of ammonia, confirming its assignment as an ammonia oxidation peak. Comparative analysis of current densities across all catalysts (Figure 3b,c) revealed outstanding performance from Pt–WO_x_ (P), which achieved a geometric peak current density of 49.67 mA cm^−2^. In contrast, Pt–WO_3_ (P), prepared using the same photodeposition method, exhibited a significantly lower peak current density of 17.34 mA cm^−2^. This discrepancy is primarily attributed to the more uniform distribution and smaller size of Pt nanoparticles in Pt–WO_x_ (P), along with stronger catalyst–support interactions that enhance intrinsic catalytic activity. The sputtered catalyst, Pt–WO_x_ (S), showed even lower activity, reaching only 6.20 mA cm^−2^, despite the expected extensive surface coverage. This reduced performance is likely due to the physical sputtering process, which typically yields weaker metal–support interactions and limited interfacial charge transfer, resulting in continuous metallic Pt films lacking optimized electronic properties. For comparison, the commercial PtIr/C and Pt/C catalysts attained peak current densities of 9.97 and 3.50 mA cm^−2^, respectively.

Also, control experiments confirmed that bare Ni foam shows negligible activity toward both AOR and HER compared with the Pt–WO_x_ (P) catalyst in Figure S15 (Supporting Information). Furthermore, a comparison between Pt–WO_x_ (P) deposited on Ni foam and on stainless steel fiber with negligible Ni sites revealed nearly identical performance, demonstrating that the observed catalytic activity originates from Pt–WO_x_ (P) rather than the substrate Figure S16 (Supporting Information).

Inductively coupled plasma optical emission spectroscopy (ICP‐OES) analysis (Table S1, Supporting Information) was employed to determine the Pt and W loadings per geometric area, allowing for a comparison of Pt‐group metal (PGM) mass‐normalized activities. A Pt loading of 0.62 mg cm^−2^ was photodeposited on the electrode surface of Pt–WO_x_ (P), while 0.81 mg cm^−2^ of Pt was deposited on Pt–WO_3_ (P). Despite identical photodeposition conditions, Pt exhibited greater aggregation on the WO_3_ support compared to WO_x_. In the sputtered catalyst, Pt–WO_x_ (S), the Pt loading was 0.38 mg cm^−2^. For the commercial catalysts prepared via drop‐casting, the noble metal (Pt or PtIr) loading was fixed at 0.8 mg cm^−2^. As shown in Figure S17 (Supporting Information), Pt–WO_x_ (P) demonstrated the highest mass activity, reaching 79.97 A g^−1^_PGM_, significantly outperforming Pt–WO_3_ (P), Pt–WO_x_ (S), PtIr/C, and Pt/C, which exhibited mass activities of 21.40, 18.78, 12.45, and 4.37 A g^−1^_PGM_, respectively representing enhancements of ≈4‐ to 18‐fold.

To further optimize AOR performance, the H_2_PtCl_6_ precursor concentration used during photodeposition was systematically varied, progressively increasing the amount of Pt deposited. This resulted in higher peak current densities with increasing precursor concentration, reaching up to 84.66 mA cm^−2^ (Figure S18, Supporting Information). Although geometric activity improved with greater Pt loading, mass activity peaked at a precursor concentration of 1 mM (79.79 A g^−1^_PGM_) and declined at higher concentrations, suggesting reduced utilization efficiency due to nanoparticle agglomeration or active site overlap (Figure S19, Supporting Information). This trend was further corroborated by CA tests normalized to Pt content.

Additionally, the surface crystallographic facets of Pt exposed during the electrochemical reaction were elucidated by analyzing *H and *OH adsorption/desorption features via cyclic voltammetry in Ar‐saturated 1.0 m KOH. The adsorption/desorption peaks corresponding to the Pt (111), Pt (110), and Pt (100) facets appeared at ≈0.05, 0.26, and 0.36 V versus RHE, respectively.^[^ ^22^, ^67^, ^68^ ^]^ As shown in Figure S20a (Supporting Information), both Pt–WO_x_ (P) and Pt–WO_3_ (P) exhibited distinct peaks for all three crystallographic facets, indicating successful formation of the desired surface structures following photodeposition. In contrast, Pt–WO_x_ (S) displayed prominent Pt (111) and Pt (110) peaks, while the Pt (100) peak, commonly associated with the highest activity for AOR, was significantly suppressed. Quantitative analysis of the peak current density ratios between Pt (100) and Pt (111) revealed a substantially higher proportion of electrochemically active Pt (100) sites on Pt–WO_x_ (P), implying that the substrate used during photodeposition significantly influences the resultant crystallographic orientation of the catalyst surface (Figure S20b, Supporting Information).

Figure 3d presents Tafel plots that further confirm the superior intrinsic kinetics of Pt–WO_x_ (P), which exhibited the lowest Tafel slope of 76.23 mV dec^−1^ among the tested catalysts, indicating enhanced electron transfer kinetics during ammonia oxidation. Correspondingly, the calculated charge transfer coefficient reached a maximum value of 0.75 for Pt–WO_x_ (P), highlighting its efficiency in facilitating electron transport during the electrocatalytic process (Figure S21, Supporting Information). The electrochemical surface areas (ECSA) of the catalysts were evaluated by measuring the double‐layer capacitance (C_dl_) via CV at scan rates ranging from 20 to 140 mV s^−1^ within the potential window of 0.90–1.00 V versus RHE (Figure S22, Supporting Information). At a representative potential of 0.95 V versus RHE, Pt–WO_x_ (P) exhibited the highest double‐layer capacitance of 24.44 mF cm^−2^, indicating the largest electrochemically active surface area among the samples. In comparison, PtIr/C and Pt–WO_3_ (P) showed significantly lower capacitances of 15.33 and 15.11 mF cm^−2^, respectively.

CA was employed to evaluate the short‐term stability of the catalysts for ammonia oxidation at a fixed potential of 0.7 V versus RHE over 500 s, as shown in Figure 3f. An initial rapid decline in current density was observed due to poisoning by nitrogen‐containing intermediates (*N and *NO_x_), which significantly reduced the availability of active Pt sites; however, a steady‐state current density was subsequently established.^[^ ^19^ ^]^ The optimized Pt–WO_x_ (P) catalyst maintained a stable current density of 11.98 mA cm^−2^ (19.32 A g^−1^_PGM_), significantly outperforming Pt–WO_3_ (P) (3.11 mA cm^−2^, 3.84 A g^−1^_PGM_), Pt–WO_x_ (S) (0.70 mA cm^−2^, 1.84 A g^−1^_PGM_), PtIr/C (0.44 mA cm^−2^, 0.55 A g^−1^_PGM_), and Pt/C (0.34 mA cm^−2^, 0.43 A g^−1^_PGM_). The inferior performance of the other catalysts underscores their susceptibility to poisoning by nitrogenous species.

In long‐term stability tests (1000 CV cycles in 0.1 m NH_3_ + 1.0 m KOH), shown in Figure S23 (Supporting Information), Pt–WO_x_ (P) demonstrated excellent durability, retaining 90.0% of its initial activity. This retention was markedly higher than that of Pt–WO_3_ (P) (75.9%), Pt–WO_x_ (S) (88.7%), PtIr/C (66.2%), and Pt/C (46.5%). The comparatively poor stability of the commercial catalysts was attributed to their weaker adhesion to the Ni foam substrate, exacerbated by repeated adsorption‐desorption cycles of nitrogen‐containing species due to the dropwise deposition method using Nafion. In contrast, post‐reaction SEM imaging of Pt–WO_x_ (P) after 1000 electrochemical cycles revealed that the nanowire architecture remained intact, with Pt nanoparticles retaining their original morphology without noticeable aggregation or structural collapse (Figure S24a, Supporting Information). Furthermore, XPS analysis of the Pt 3d region showed that the Pt^0^ peak at 2121.89 eV^[^ ^69^ ^]^ remained unchanged after 100 and 1000 cycles, indicating no detectable shift in the oxidation state of Pt during the AOR process (Figure S24b, Supporting Information). These results strongly suggest that the catalyst possesses excellent structural and electronic stability under extended electrochemical operation, underscoring its robustness for ammonia oxidation.

Further kinetic investigations into the AOR were conducted by varying electrolyte compositions and reaction conditions. As shown in Figure S25a (Supporting Information), increasing the KOH concentration from 1 to 5 m reduced the onset potential from 0.46 to 0.43 V versus RHE and significantly increased the peak current density to 65.9 mA cm^−2^, highlighting the essential role of OH^−^ ions in enhancing reaction kinetics and facilitating the desorption of intermediates. Similarly, as depicted in Figure S25b (Supporting Information), increasing the ammonia concentration significantly elevated the peak current density to 82.3 mA cm^−2^, which indicates that ammonia concentration serves as a critical rate‐determining factor in accordance with Nernstian behavior. Temperature‐dependent experiments conducted at 25, 40, and 60 °C further demonstrated a substantial increase in peak current density, from 49.97 mA cm^−2^ at 25 °C to 106.1 mA cm^−2^ at 60 °C (Figure 3g), suggesting that elevated temperatures enhance the feasibility of the rate‐limiting N–N coupling step and improve overall reaction kinetics. An Arrhenius analysis based on these data (Figure 3h) revealed that the activation energy at 0.6 V versus RHE was lower for Pt–WO_x_ (P) (29.56 kJ mol^−1^) compared to PtIr/C (33.27 kJ mol^−1^). These results prove greater temperature sensitivity and superior catalytic performance of Pt–WO_x_ (P) under practical cell operating conditions.

Moreover, a comparative analysis in Figure 3i, alongside recently reported ammonia oxidation electrocatalysts from the literature (Table S3, Supporting Information), revealed that the Pt–WO_x_ (P) catalyst exhibited the highest peak current density (49.67 mA cm^−2^) and mass activity (79.97 A g^−1^_PGM_), underscoring its exceptional performance and efficient utilization of precious metal resources. These enhanced properties are primarily attributed to favorable electronic interactions between Pt nanoparticles and WO_x_ nanowires, which facilitate rapid electron transfer and reduce energy barriers for nitrogen species adsorption and desorption, thereby significantly improving the catalyst's resistance to nitrogen poisoning.

### 2.4 ATR‐SEIRAS for AOR Mechanism Study

**Figure 4:**
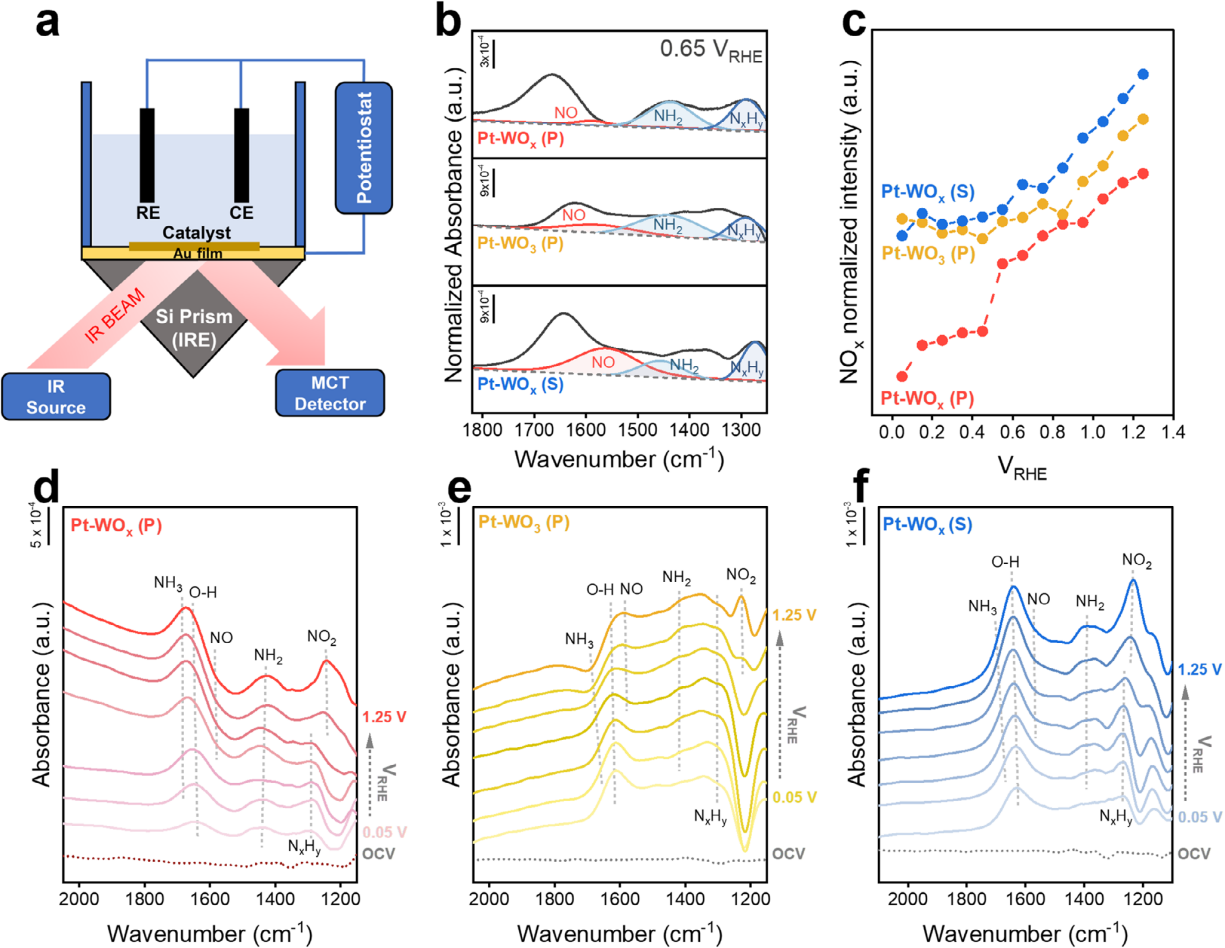
ATR‐SEIRAS analysis of the AOR on Pt–WO_x_ catalysts. a) Schematic illustration of the ATR‐SEIRAS experimental setup using a Si prism and Au film. b) SEIRA spectra in the 1850–1250 cm^−1^ region collected at 0.65 V versus RHE, showing key vibrational features assigned to NO, NH_2_, and N_x_H_y_ species after baseline correction. c) Integrated SEIRA peak intensities of NO (1540–1570 cm^−1^) and NO_2_ (1230–1270 cm^−1^) as a function of applied potential. Potential‐dependent SEIRA spectra (2100–1150 cm^−1^) obtained during multi‐step CA from 0.05 V to 1.25 V versus RHE on d) Pt–WO_x_ (P), e) Pt–WO_3_ (P), and f) Pt–WO_x_ (S) electrodes in Ar‐saturated 1 M KOH + 0.1 m NH_3_. All spectra were recorded after a 2 min potential hold, using the open‐circuit potential (OCV) spectrum as the reference.

To further elucidate the reaction mechanism and intermediates responsible for the enhanced catalytic activity, ATR‐SEIRAS measurements were conducted. As shown in **Figure** **4**a, in situ spectra were collected by applying a series of electrochemical potentials while simultaneously capturing infrared signals reflected from the electrode–electrolyte interface using a Fourier‐transform infrared spectrometer equipped with a variable‐angle specular reflectance accessory and a liquid‐nitrogen‐cooled MCT detector. Catalyst inks were prepared by dispersing the synthesized catalysts in isopropanol with Nafion binder and spray‐coated onto an electroless gold‐coated hemicylindrical silicon prism, which served as the working electrode. The applied potential was increased stepwise from 0.05 V versus RHE in 0.2 V increments, with absorbance measured after holding each potential for 2 min in a solution of 1 m KOH + 0.1 m NH_3_. For the Pt‐WO_x_ (P) catalyst, a distinct vibrational peak at 1260 cm^−1^, attributed to N_x_H_y_ (x = 1–2) species, emerged at low potentials below 0.45 V (Figure 4d).^[^ ^70^ ^]^ Simultaneously, the intensity of the NH_2_‐associated peak at 1430 cm^−1^ increased progressively with applied potential.^[^ ^70^ ^]^ These spectral features suggest that Pt‐WO_x_ (P) facilitates N_2_ formation via N_x_H_y_ dimerization, consistent with the Gerischer–Mauerer mechanism. All three catalysts (Pt–WO_x_ (P), Pt–WO_3_ (P), and Pt–WO_x_ (S)) exhibited N_x_H_y_ peaks at low potentials, indicating early intermediate formation. However, the NH_2_ peak at 1430 cm^−1^ was more pronounced for Pt–WO_x_ (P), implying more efficient ammonia activation at lower voltages (Figure 4d–f). In contrast, the NH_2_ peak in Pt–WO_3_ (P) appeared less distinctly and only at relatively higher potentials, suggesting a delayed onset of the AOR.

At higher potentials relevant to intermediate and byproduct formation, a vibrational peak at 1560 cm^−1^, attributed to NO species, began to increase above 0.65 V.^[^ ^71^ ^]^ Notably, peak deconvolution revealed that Pt–WO_x_ (P) exhibited significantly lower NO byproduct formation at 0.65 V compared to Pt–WO_3_ (P) and Pt–WO_x_ (S) (Figure 4b). Additionally, a NO_2_‐associated peak at 1240 cm^−1^ was observed to grow above 0.95 V on all three catalysts—Pt–WO_x_ (P), Pt–WO_3_ (P), and Pt–WO_x_ (S) (Figure 4d–f).^[^ ^72^ ^]^ When the intensities of the NO and NO_2_ peaks were normalized across the catalysts, the order of peak growth followed the trend: Pt–WO_x_ (S) > Pt–WO_3_ (P) > Pt–WO_x_ (P) (Figure 4c), indicating that the accumulation of poisoning nitrogenous species at elevated potentials was least pronounced on Pt–WO_x_ (P). These findings collectively demonstrate that Pt–WO_x_ (P) not only facilitates efficient ammonia activation at lower potentials but also suppresses the formation and accumulation of deactivating intermediates such as NO and NO_2_ at higher potentials. Figure S26 (Supporting Information) compares spectral differences observed in 1 m KOH and 1 m KOH + 0.1 m NH_3_ electrolytes for each catalyst, highlighting the distinct electrochemical behaviors associated with OER and AOR. These results further support the trends observed in the main figures and underscore catalyst‐dependent variations in reaction pathways and intermediate formation. ATR‐SEIRAS measurements were also conducted on commercial Pt/C and PtIr/C catalysts. Although a direct quantitative comparison was limited due to intrinsic differences in sampling protocols—specifically between the self‐supported catalysts synthesized via direct growth on Ni foam and the conventional powder‐based catalysts—linear sweep voltammetry (LSV) confirmed the formation of intermediates and poisoning species on both commercial samples. These observations reinforce the utility of ATR‐SEIRAS for assessing AOR activity and the accumulation of deactivating species, as illustrated in Figure S27 (Supporting Information).

### 2.5 DFT Calculation of Charge Transfer and Catalytic Activity in Pt–WO_x_ (P)

To support the experimental findings regarding the superior catalytic activity of Pt–WO_x_ (P), we performed density functional theory (DFT) calculations. For comparison, photodeposited Pt–WO_x_ (P) and Pt–WO_3_ (P) samples were considered, as they feature Pt nanoparticles with similar (100) surface facets but differ in the stoichiometry of their WO_3_‐based supports. Since our study focuses on the stoichiometry effect of WO_3_‐based supports, not the effect of deposition method, the sputtered Pt–WO_x_ (S) sample was excluded from our consideration. In our high‐magnification FESEM measurement for Pt–WO_x_ (P) and Pt–WO_3_ (P) samples, Pt–WO_x_ (P) exhibits uniformly distributed and smaller Pt nanoparticles than Pt–WO_3_ (P) (Figure 1b; Figures S3 and S4, Supporting Information). To elucidate this distinct growth behavior of Pt nanoparticles, WO_x_, WO_3,_ and Pt slab structures were constructed to represent the exposed surfaces of WO_x_ and WO_3_ supports and Pt nanoparticles. High‐resolution TEM analysis (Figure 1e,f; Figure S9, Supporting Information) revealed that the exposed surfaces correspond to WO_x_ (100), WO_3_ (001), and Pt (100); these surfaces were therefore adopted in the slab structures. The most stable terminations of WO_x_ (100) and WO_3_ (001) were determined by evaluating their surface energies. Full structural details of all slab structures are provided in the DFT Calculation section of Figures S28–S30 (Supporting Information).

**Figure 5:**
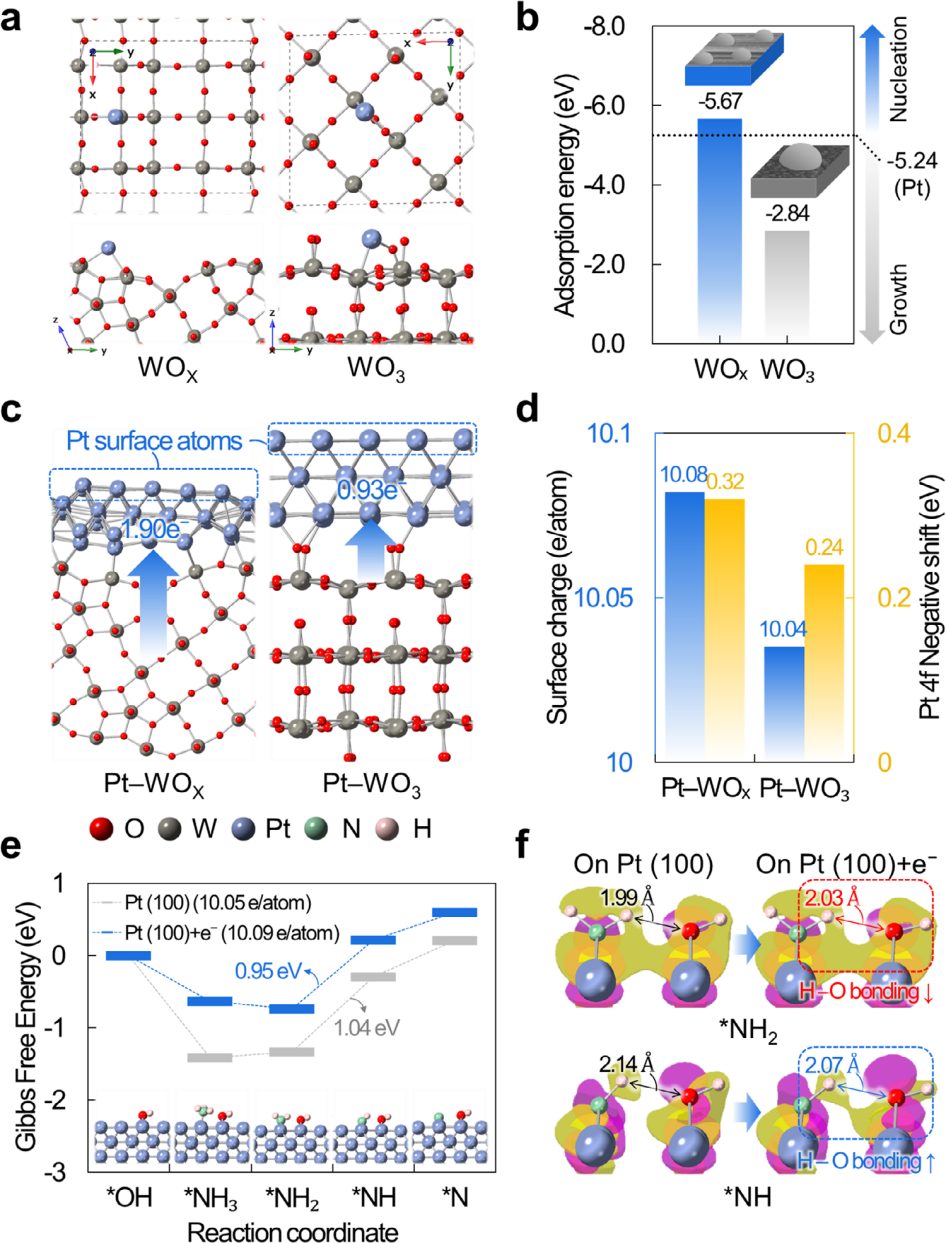
a) Adsorption configuration of a Pt atom on WO_x_ (100) and WO_3_ (001) slabs. b) Calculated adsorption energies of a Pt atom on WO_x_ (100) and WO_3_ (001) slabs. c) Relaxed interface structures of Pt–WO_x_ and Pt–WO_3_. The number of transferred electrons is calculated by Bader charge analysis. d) The surface charge of Pt surface atoms (blue) and corresponding Pt 4f negative shifts of XPS measurements (yellow) for Pt–WO_x_ and Pt–WO_3_ interfaces. e) Gibbs free energy diagram for ammonia oxidation reaction on OH‐covered Pt (100) and Pt (100)+e^−^ surfaces; calculated surface charges are given in brackets. f) Charge density difference for *NH_2_ and *NH adsorbates on OH‐covered Pt (100) and Pt (100)+e^−^ surfaces (isosurface = 0.02 e/Å^3^); magenta and yellow denote electron accumulation and depletion, respectively.

Subsequently, the adsorption energies of a single Pt atom on WO_x_, WO_3_, and Pt slabs were calculated to evaluate which surface the Pt atom prefers to adsorb onto. When the Pt adsorption energy on WO_x_ (or WO_3_) slab is more negative than on Pt slab, the Pt atom preferentially adsorbs on WO_x_ (or WO_3_) support, leading to the nucleation of a new Pt nanoparticle. Conversely, when the Pt adsorption energy on WO_x_ (or WO_3_) slab is less negative than on Pt slab, the Pt atom preferentially adsorbs on existing Pt nanoparticles, promoting the growth of Pt nanoparticles. Based on this assumption, the most stable adsorption configurations of a Pt atom were identified by energetically evaluating the grid‐searched adsorption sites on WO_x_ (100), WO_3_ (001), and Pt (100) slabs. The structural details of Pt‐adsorbed WO_x_ (100), WO_3_ (001), and Pt (100) slabs are in Figure S31 (Supporting Information). **Figure** **5**a shows the most stable adsorption configurations of a Pt atom on WO_x_ (100) and WO_3_ (001) slabs. On WO_x_ (100) (left), the Pt atom binds with two W atoms, whereas on WO_3_ (001) (right), it binds to one W atom and one O atom. The corresponding adsorption energies are presented in Figure 5b.

On WO_x_ (100), the adsorption energy is −5.67 eV, which is more negative than −5.24 eV on Pt (100), thus Pt nucleation is favored. In contrast, on WO_3_ (001), the adsorption energy of −2.84 eV is much weaker than −5.24 eV on Pt (100), thus the growth of existing Pt nanoparticles is preferred. These calculation results suggest the formation of more numerous and smaller‐sized Pt nanoparticles on WO_x_ support than on WO_3_ support, in agreement with high‐magnification FE‐SEM observations (Figure 1b; Figures S3 and S4, Supporting Information) and the corresponding particle‐size distributions (Figure 1h,i) for Pt–WO_x_ (P) and Pt–WO_3_ (P). Therefore, Pt–WO_x_ (P) increases the Pt surface area owing to the preference of Pt nucleation, then exhibits the highest mass activity of 79.79 A g^−1^_PGM_ (Figure S17b, Supporting Information).

Since the ammonia oxidation reaction mainly occurred on the Pt nanoparticle surfaces, we further modeled the Pt nanoparticle surfaces of Pt–WO_x_ (P) and Pt–WO_3_ (P). Since our XPS measurements revealed that Pt–WO_x_ (P) transfers the relatively larger electron density from the support to Pt nanoparticles than Pt–WO_3_ (P) (Figure 2e), we first estimated the interfacial electron transfer by constructing Pt–WO_x_ and Pt–WO_3_ interface structures. The interface structures were constructed by placing 3 layers of Pt (100) on WO_x_ (100) and WO_3_ (001) slabs with minimizing the lattice mismatch, and full structural details were provided in DFT Calculations section of Figure S32 (Supporting Information). As shown in Figure 5c, the relaxed interface structures of Pt–WO_x_ and Pt–WO_3_ represent that the Pt layers strongly interact with WO_x_ support compared to WO_3_ support, which has agreement with our FT‐EXAFS analysis (Figure 2b). The Bader charge analysis also confirms these results: 1.90 e^−^ are transferred from WO_x_ to Pt layers, whereas only 0.93 e^−^ are transferred from WO_3_ to Pt layers (Figure 5c). Our calculation results demonstrate that the relatively stronger metal–support interactions and electron transfer for Pt–WO_x_ compared to Pt–WO_3_. Given that the ammonia oxidation reaction is closely related to charge distribution on the Pt surface atoms, we further analyzed the surface charges of the Pt–WO_x_ and Pt–WO_3_ systems (Figure 5d, blue). The surface charge is defined as the average Bader charge of the Pt surface atoms. We observed higher surface charges on Pt–WO_x_ (10.08 e^−^ atom^−1^) than on Pt–WO_3_ (10.04 e^−^ atom^−1^), indicating a higher electron density of the Pt surface atoms on Pt‐WO_x_ compared to those on Pt‐WO_3_. The negative shift of Pt 4f in our XPS measurement well supports these results, which the larger negative shift of Pt−WO_x_ (P) (0.32 eV) than Pt−WO_3_ (P) (0.24 eV) indicates the more electron transfer to Pt nanoparticles from WO_x_ support than WO_3_ support (Figure 5d, yellow). Therefore, these findings verify that Pt−WO_x_ (P) possesses greater electron transfer from the support to Pt nanoparticles and higher electron density on the Pt nanoparticle surfaces.

To explore how surface charge influences the catalytic activity of Pt nanoparticles, we further modeled the surface of Pt nanoparticles using a bulk Pt (100) slab, as the actual Pt nanoparticles on WO_x_ and WO_3_ are several nanometers thick (Figure 1h,i). To simulate the increase of surface charge on Pt−WO_x_, we added an extra electron to the Pt (100) slab and constructed a Pt (100)+e^−^ slab. Then, we compared the surface charges of Pt (100) and Pt (100)+e^−^ slabs with those of Pt‐WO_x_ and Pt‐WO_3_. The calculated surface charge is 10.05 e^−^ per atom for Pt (100) slab and 10.09 e^−^ per atom for Pt (100)+e^−^ slab, which closely match the surface charges of Pt−WO_3_ and Pt−WO_x_ interfaces (10.04 and 10.08 e^−^ per atom, respectively) in Figure 5d. This agreement suggests that our model Pt (100) and Pt (100)+e^−^ slabs reasonably approximate the Pt nanoparticle surfaces on both WO_x_ and WO_3_ supports. Since ATR‐SEIRAS measurements provided evidence for the Gerischer−Mauerer (G−M) mechanism of the ammonia oxidation reaction (Figure 4), in which the adsorbed NH_3_ molecule is sequentially dehydrogenated by OH^−^ ions,^[^ ^73^ ^]^ OH‐covered Pt (100) and Pt (100)+e^−^ surfaces (1/9 ML) were considered. The adsorption configuration of *OH was evaluated for all top, bridge, and hollow sites on Pt (100) and Pt (100)+e^−^ surfaces, and the bridge site is identified as the most stable configuration. The reaction pathways of the ammonia oxidation reaction were established by identifying the most stable adsorption configurations of *NH_3_, *NH_2_, *NH, and *N on the OH‐covered Pt (100) surface. Finally, the effect of surface charge was examined by calculating the Gibbs free energies along these pathways. Our calculated Gibbs free energy profiles are consistent with trends reported in previous theoretical studies,^[^ ^43^, ^74^ ^]^ and further details regarding the adsorption configurations are provided in the DFT Calculation section of the Figure S43 and Tables S4 and S5 (Supporting Information)

Figure 5e presents the Gibbs free energy diagram for ammonia oxidation reaction on OH‐covered Pt (100) and Pt (100)+e^−^ surfaces. For Pt (100) surface, *NH_3_ adsorption is energetically stabilized, but the dehydrogenations from *NH_3_ to *N need external energies to overcome their energy barriers, with the highest energy barrier of 1.04 eV at the rate‐determining step (RDS) from *NH_2_ to *NH. In contrast, for Pt (100)+e^−^ surface, the dehydrogenation from *NH_3_ to ^*^NH_2_ becomes thermodynamically favorable, and the energy barrier at RDS decreases to 0.95 eV. These results indicate that the strong electron transfer from WO_x_ support to Pt nanoparticles increases the surface charge of Pt, thereby enhancing the ammonia oxidation reaction by lowering the energy barrier at RDS. Charge density difference analysis for *NH_2_ and *NH adsorbates on both Pt (100) and Pt (100)+e^−^ surfaces further supports this result (Figure 5f). For *NH_2_, with an extra electron, the H─O bond is elongated from 1.99 to 2.03 Å and no significant change in charge distribution is observed on the corresponding H and O ions. These results indicate no enhancement of hydrogen bonding between *NH_2_ and *OH adsorbates. In contrast, for *NH, the H─O bond is shortened from 2.14 to 2.07 Å and increased electron accumulation is observed on the corresponding O ion with an extra electron, indicating enhanced hydrogen bonding between *NH and *OH adsorbates. As the surface charge increases, the H─O interaction between *NH and *OH adsorbates is stronger, thereby stabilizing *NH adsorption more than *NH_2_ adsorption and lowering the energy barrier at RDS. In our DFT calculations, the Pt surface was modeled under OH coverage, as surface OH species play a critical role in assisting the H dehydrogenation process. Therefore, we performed kinetic isotope effect (KIE) measurements by replacing H_2_O with D_2_O to further substantiate the involvement of H transfer in the rate‐determining step. As shown in Figure S34 (Supporting Information), the main catalyst Pt−WO_x_ (P) exhibited a KIE value of 1.647, while the control Pt−WO_3_ (P) showed a higher value of 2.011. Since both values are greater than 1, these results confirm that hydrogen transfer is indeed involved in the RDS, consistent with our DFT prediction. Importantly, the lower KIE observed for Pt−WO_x_ (P) suggests that the electron‐enriched Pt surface facilitates more efficient proton‐coupled electron transfer. This finding is in good agreement with the calculated decrease in the *NH_2_ to *NH energy barrier on electron‐enriched Pt (Figure 5e,f), highlighting the synergistic effect of WO_x_ support in promoting the ammonia oxidation reaction. In conclusion, the superior catalytic activity of Pt–WO_x_ (P) is attributed to 1) the increased surface area resulting from the more numerous and smaller‐sized Pt nanoparticles, and 2) the reduced energy barrier at RDS enabled by strong electron transfer from the WO_x_ support to the Pt nanoparticle surface.

### 2.6 Ammonia Electrolysis System and H_2_ Production

**Figure 6:**
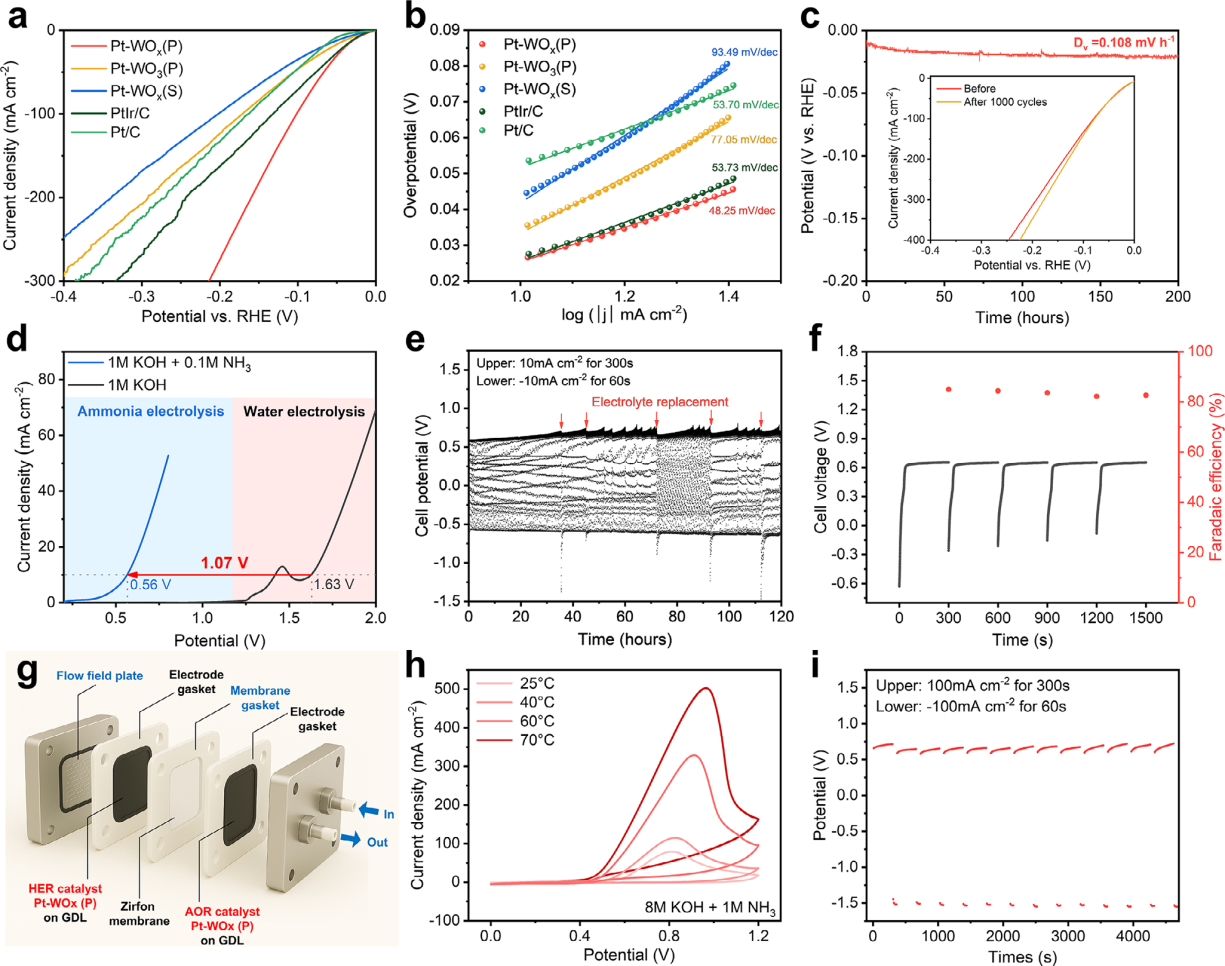
a) LSV curves of HER and b) Tafel plots of Pt–WO_x_ (P), Pt–WO_3_ (P), Pt–WO_x_ (S), PtIr/C, and Pt/C. c) CP profile at 10 mA cm^−2^ for 200 h (inset: LSV curves before and after 1000 HER cycles). d) Polarization curves for ammonia electrolysis and water electrolysis in a two‐electrode H‐type cell. e) Chronopotentiometry (CP) test with pulsed current conditions (upper current: 10 mA cm^−2^ for 300 s, lower current: −10 mA cm^−2^ for 60 s). f) Applied cell voltages and Faradaic efficiency (FE) of H_2_ production at 10 mA cm^−2^. g) Schematic illustration of the ammonia electrolysis flow‐type cell (active catalyst area: 5 cm^2^). h) CV curves in 8 m KOH + 1 m NH_3_ at different temperatures in the ammonia electrolysis flow cell. i) CP profiles under pulsed current conditions (upper current: +100 mA cm^−2^ for 300 s, lower current: −100 mA cm^−2^ for 60 s) in the ammonia electrolysis flow cell.

To evaluate the bifunctionality of the catalysts for ammonia‐based low‐energy hydrogen production systems, we investigated their HER activity. LSV measurements were performed in Ar‐saturated 1.0 m KOH electrolyte at a scan rate of 5 mV s^−1^ without iR compensation. As shown in **Figure** **6**a, Pt–WO_x_ (P) exhibited superior HER activity compared to commercial Pt‐based catalysts. The overpotential required to reach 10 mA cm^−2^ was only 26.0 mV for Pt–WO_x_ (P), outperforming Pt–WO_3_ (P) (35.0 mV), Pt–WO_x_ (S) (44.0 mV), PtIr/C (27.0 mV), and Pt/C (53.0 mV). At a higher current density of 100 mA cm^−2^, Pt‐WO_x_ (P) required just 103 mV, significantly lower than Pt–WO_3_ (P) (172 mV), Pt–WO_x_ (S) (203 mV), PtIr/C (133 mV), and Pt/C (164 mV). These results highlight that Pt–WO_x_ (P) not only operates efficiently at low overpotentials but also maintains excellent performance under high current density conditions, making it highly promising for large‐scale hydrogen production where high‐voltage operation is essential.

Tafel slope analysis (Figure 6b) further confirms the superior HER kinetics of Pt–WO_x_ (P), which exhibited the lowest slope of 48.25 mV dec^−1^, indicating efficient proton‐coupled electron transfer and rapid HER dynamics. Long‐term HER stability was assessed via CA at −10 mA cm^−2^ over 200 h (Figure 6c), during which Pt–WO_x_ (P) showed a minimal overpotential increase of only 0.108 mV h^−1^, confirming its excellent durability. Additionally, after 1000 CV cycles, the LSV profiles of Pt–WO_x_ (P) remained virtually unchanged (Figure S35a, Supporting Information), indicating structural and electrochemical robustness. XPS analysis conducted before and after 100 CV cycles showed no shift in the Pt^0^ peak (2121.89 eV), further demonstrating the stability of the Pt oxidation state throughout HER operation (Figure S35b, Supporting Information). Given the growing demand for ammonia electrolyzers employing symmetrical catalysts,^[^ ^75^ ^]^ we constructed a two‐electrode system using Pt–WO_x_ (P) as both the anode and cathode in a custom‐designed H‐type cell (Figure S36, Supporting Information) to evaluate its applicability as a bifunctional electrocatalyst. Bifunctional Pt–WO_x_ (P) electrodes were employed as both the anode and cathode for facilitating AOR and HER. In a control experiment using 1 m KOH as the anolyte, corresponding to conventional OER–HER electrolysis, the cell required 1.63 V to reach a current density of 10 mA cm^−2^. In contrast, the addition of 0.1 m NH_3_ to the anolyte significantly reduced the required cell voltage to 0.56 V, achieving a 1.07 V reduction in energy input, as shown in Figure 6d. These results confirm the feasibility of low‐voltage hydrogen production by leveraging the thermodynamic advantage of AOR.

Recent studies on AOR have increasingly adopted pulsed current or potential methods, where oxidative AOR is conducted under positive current, followed by reductive pulses at negative current to remove nitrogen‐related poisoning species from the anodic catalyst surface.^[^ ^67^, ^76^ ^]^ Indeed, as shown in Figure S37 (Supporting Information), when electrolysis was operated under a constant current density of 10 mA cm^−2^, the voltage profile revealed a rapid increase beyond 0.8 V after 8.6 h, which indicates oversaturation of the catalyst surface with nitrogen and O/OH adsorbates. Moreover, when the operation was extended beyond 8.8 h, the cell voltage exceeded 1.4 V, demonstrating that the anodic reaction had shifted from AOR to OER. To avoid this poisoning issue, a pulsed current protocol was implemented to assess the operational stability of the ammonia electrolyzer. The protocol alternated between 10 mA cm^−2^ for 300 s and −10 mA cm^−2^ for 60 s, driving AOR at the anode and HER at the cathode. As shown in Figure 6e, the ammonia electrolysis system maintained stable operation below 0.75 V_cell_ for over 120 h, significantly surpassing the stability benchmarks reported in prior AOR studies. A more detailed portion of the time‐dependent cell potential profile is provided in Figure S38 (Supporting Information) for further insight. This exceptional durability is attributed to the tailored electronic structure of Pt induced by WO_x_ during photodeposition, which enables both the optimal adsorption of NH_x_ intermediates to enhance AOR activity and the effective removal of NO_x_‐related poisoning species from the catalyst surface. Following the 120 h stability test, nitrite (NO_2_^−^) and nitrate (NO_3_^−^) ions in the electrolyte were quantified to evaluate the formation of by‐products. Figures S39 and S40 (Supporting Information) present the calibration curves for NO_2_^−^ and NO_3_^−^, respectively, obtained from concentration‐dependent absorbance measurements using a UV–Vis spectrometer. Based on these calibration data, the post‐electrolysis electrolyte exhibited generation rates of 0.492 µmol h^−1^ for NO_2_^−^ and 0.355 µmol h^−1^ for NO_3_^−^. Considering the initial ammonia concentration of 0.1 m, these values indicate that the formation of NO_x_ by‐products was strongly suppressed. This observation is consistent with the ATR‐SEIRAS results, which also revealed effective inhibition of NO_x_ species under the applied conditions.

Also, as shown in Figure S41a (Supporting Information), XPS analysis after 120 h stability testing revealed that the Pt 4f_7/2_ peak remained at 70.01 eV, which confirms the preservation of metallic Pt^0^ without valence change. The N 1s spectrum in Figure S41b (Supporting Information) further exhibited signals at 402.54, 397.89, and 396.32 eV, corresponding to NO, NH_x_, and M–N species, respectively, consistent with previously reported AOR intermediates. Post‐stability TEM analysis confirmed that Pt nanoparticles remained uniformly dispersed on the WO_x_ support without agglomeration even after 120 h (Figure S42a, Supporting Information). High‐resolution TEM and FFT analysis further revealed that the Pt (100) and (111) facets were well preserved (Figure S42b, Supporting Information). Elemental mapping further verified homogeneous Pt distribution across the nanowires (Figure S42c, Supporting Information), consistent with the absence of particle sintering. In addition, as shown in Figure S43 (Supporting Information), XRD peak deconvolution showed an increase in the Pt (200)/Pt (111) intensity ratio from 0.549 to 0.613 (Table S2, Supporting Information), suggesting that a larger fraction of {100} facets was preserved relative to {111}. Electrochemical probing through CV in 1 m KOH consistently supported this observation: the Pt (100) hydrogen (hydroxide) adsorption peak current density increased after long‐term operation, accompanied by a higher Pt (100)/ Pt (111) ratio in Figure S44 (Supporting Information). Taken together, these results demonstrate that Pt (100) facets known to be favorable for AOR activity are preferentially retained on the catalyst surface, thereby providing a structural basis for the sustained catalytic performance during extended operation.

Additionally, hydrogen production at the cathode was quantitatively monitored via gas chromatography every 5 min, as shown in Figure 6f, with an average FE of 83.6%, indicating that AOR‐driven electrolysis is an exceptionally energy‐efficient pathway for sustainable hydrogen production. Also, at the anode, gas‐phase analysis after 30 min of ammonia electrolysis at the anode confirmed selective N_2_ production with a Faradaic efficiency of 98.49 ± 2.85% by gas chromatography in Figure S45 (Supporting Information). Gas phase FT‐IR measurements further showed no detectable N_2_O or NO signals (Figure S46, Supporting Information), highlighting that byproduct formation was negligible.

To explore the scalability and practical integration of the bifunctional catalyst, an MEA‐based flow cell with a 5 cm^2^ active area was fabricated (Figure 6g; Figure S47, Supporting Information). Both the anode and cathode consisted of Ni fiber‐based gas diffusion layers, onto which WO_x_ NWs were directly grown and subsequently modified with Pt via photodeposition, ensuring a high surface area and optimal bifunctionality. For the membrane, a Zirfon Perl UTP 500 separator (0.5 mm thickness) was selected due to its proven chemical stability in concentrated alkaline environments and elevated temperatures, as well as its high ionic conductivity and favorable gas permeability, which facilitates efficient gas diffusion during operation.^[^ ^77^ ^]^ Flow field plates were employed to deliver high‐concentration KOH‐based ammonia electrolytes (8 m KOH with 1 m NH_3_ as both anolyte and catholyte) to the electrodes, while additional gaskets were used to prevent electrolyte leakage. Using this flow cell configuration with the bifunctional Pt–WO_x_ (P) catalyst, a remarkable current density of 501.9 mA cm^−2^ was achieved under 8 m KOH with 1 m NH_3_ at 70 °C. Temperature‐dependent polarization tests (Figure 6h) revealed a dramatic enhancement in current density with increasing temperature: 79.1 mA cm^−2^ at 25 °C, 114.9 mA cm^−2^ at 40 °C, 329.1 mA cm^−2^ at 60 °C, and 501.9 mA cm^−2^ at 70 °C. The enhanced performance at elevated temperatures is attributed to improved mass transport, reduced electrolyte resistance, and the favorable transition to a two‐phase flow regime, which accelerates ammonia delivery to the active sites.

To probe the stability of the system under flow conditions, a pulsed current protocol was implemented, consisting of an upper current of +100 mA cm^−2^ for 300 s and a lower current of −100 mA cm^−2^ for 60 s. The system maintained voltages below 0.75 V_cell_ for over 4500 s without any significant voltage surge into the OER regime, indicating effective mitigation of nitrogen‐species poisoning even under industrially relevant current densities (Figure 6i). These results underscore the practical potential of the Pt–WO_x_ (P) electrode and Zirfon‐based system as a durable and energy‐efficient platform for ammonia‐enabled hydrogen production. Importantly, incorporating a flow‐cell configuration into AOR systems provides a viable strategy for overcoming mass transport limitations and mitigating surface poisoning effects commonly encountered under static conditions. This study represents a significant advancement toward the development of low‐voltage hydrogen production from ammonia and may inform the design of future systems aimed at integrating AOR into scalable electrolysis technologies.

## 3 Conclusion

In this study, we demonstrated that Pt–WO_x_ (P) enables efficient and durable ammonia electrolysis by addressing both the activity and stability challenges inherent to AOR. The metal–support interaction induced by photodeposition facilitates electron transfer from WO_x_ to Pt, increasing the electron density in the Pt 5d orbitals. ATR‐SEIRAS analysis confirmed the Gerischer–Mauerer mechanism and demonstrated that Pt–WO_x_ (P) generates the least amount of NO_x_ poisoning species among the tested catalysts. DFT calculations revealed that the Pt electronic structure, modulated by WO_x_, plays a pivotal role in lowering the energy barrier for the rate‐determining NH_2_ to NH conversion step. The catalyst exhibited excellent AOR performance, achieving a peak current density of 49.69 mA cm^−2^ with a mass activity of 79.97 A g^−1^_PGM_. Moreover, Pt–WO_x_ (P) demonstrated outstanding long‐term stability, sustaining operation for 120 h and achieving a high current density of 501.9 mA cm^−2^ in the 5 cm^2^ flow‐type cell. By integrating molecular‐level mechanistic insights with device‐level validation, this work represents a critical step toward realizing efficient, low‐voltage ammonia electrolysis systems for sustainable hydrogen production.

## 4 Experimental Section

### Materials

Tungsten oxide powder (WO_3_), chloroplatinic acid hexahydrate (H_2_PtCl_6_·6H_2_O, 99.9%), platinum on carbon (Pt/C, 10 wt.%), nafion (5 wt.%), and deuterium oxide (D_2_O) were purchased from Sigma Aldrich. Commercial PtIr/C (40 wt.%) was obtained from Premetek Co. Absolute methanol was obtained from Fisher Scientific Korea Ltd. (Seoul, Korea). Potassium hydroxide solution (KOH, 1 m) and ammonium hydroxide solution (NH_4_OH, 28−30 wt.%) were provided by Samchun Chemicals. Nickel foam, nickel fiber paper, and stainless steel (SS) fiber for substrates were purchased from MTI Korea. To remove the oxide layer of the substrate, the nickel foam and nickel fiber paper were cleaned by sonication in ethanol, acetone, and DI water for 10 min each.

### Characterizations

Surface morphology of the catalysts was examined using field emission scanning electron microscopy (FE‐SEM; JSM‐7401F, JEOL; XL30S with dual EDS, Philips). High‐resolution images, EDS mapping of the catalysts, and FFT patterns were investigated by transmission electron microscopy (TEM; JEM‐2200FS equipped with an image Cs corrector, JEOL). Crystal structure analysis was performed using X‐ray diffraction (XRD; D/MAX‐2500/PC with Cu Kα radiation source, Rigaku). The chemical states of surface elements were analyzed via X‐ray photoelectron spectroscopy (XPS; NEXSA G2, Thermo Fisher Scientific). Only the analysis of Pt 3d orbitals was conducted in SPring‐8 BL09XU. All XPS data were calibrated by referencing binding energies to the C 1s peak positioned at 284.8 eV. Electronic structures at the Pt L_2_‐edge, Pt L_3_‐edge, and W L_3_‐edge were investigated by X‐ray absorption spectroscopy (XAS; beamline BL14B2, SPring‐8, Super Photon ring 8 GeV, Japan). Inductively coupled plasma atomic emission spectroscopy (ICP‐AES) measurements were carried out using an ARCOS instrument (SPECTRO) to determine the elemental composition of Pt and W in the samples.

### Electrochemical Measurements

Electrochemical measurements were conducted using a three‐electrode setup connected to a potentiostat (CHI 760E, Ivium Technologies). A Hg/HgO electrode and a graphite rod were employed as the reference and counter electrodes, respectively. All recorded potentials were converted to the reversible hydrogen electrode (RHE) scale using the equation:

(4) $$V_{RHE}=V_{measured}+V_{Hg/HgO}+0.0591\times pH$$

Prior to evaluating ammonia oxidation reaction (AOR) performance, the catalyst was electrochemically activated by conducting 20 cycles of cyclic voltammetry (CV) in 1.0 m KOH at a scan rate of 50 mV s^−1^ and recorded at a scan rate of 20 mV s^−1^. Subsequent AOR activity was assessed by CV in an electrolyte containing Ar‐saturated 1.0 m KOH and 0.1 m NH_3_, using a scan rate of 5 mV s^−1^ at 0.2–1.2 V versus RHE. To investigate the influence of operating temperature, ammonia concentration, and KOH concentration on catalytic performance, a newly prepared working electrode was employed for each individual test. The electrochemical surface area (ECSA) was estimated by evaluating the double‐layer capacitance (C_dl_), which was derived from CV conducted in a non‐faradaic potential window ranging from 0.9 to 1.0 V versus RHE. Tafel slopes were calculated by the linear portion of the potential versus log (current density) plot to evaluate reaction kinetics. The charge transfer coefficient (α) was calculated using the following relation: α = (2.3 × R × T) / (b × F), where R is the gas constant, T is the temperature, F is the Faraday constant, and b is the measured Tafel slope.

### Ammonia Electrolysis Flow Cell Test

The ammonia electrolysis flow cell was assembled using nickel flow fields, a PTFE gasket, O‐rings, and an insulating kit, all procured from Dioxide Materials. Electrolyte circulation was maintained by a peristaltic pump (EMP‐600G2, EMS TECH) at a constant flow rate of 100 mL min^−1^. The temperature of the electrolyte was controlled using a hot plate stirrer (HSD 150‐03P, Misung) in conjunction with a thermocouple. The membrane used in the flow cell was a Zirfon separator (UTP 500+, Agfa; 0.5 mm thickness), obtained from Yulim Engineering. Electrochemical performance of the ammonia electrolysis flow cell was evaluated using a potentiostat (CHI 760E, Ivium Technologies) connected via high‐power cable to accommodate the demanding operational conditions.

## Conflict of Interest

The authors declare no conflict of interest.

## Supporting information



Supporting Information

## Data Availability

The data that support the findings of this study are available in the supplementary material of this article.
