# Supplementary material for: Selective and Long‐Term Stable Ammonia Electrolysis Using Pt–WOx Catalysts with Suppressed NOx Formation and Enhanced Activity
Source: Adv Sci (Weinh). 2025 Oct 27;13(3):e15944. doi: 10.1002/advs.202515944 (PMC12806440; doi:10.1002/advs.202515944)
Supplement: Supplementary file 1 — Supporting Information [file ADVS-13-e15944-s001.docx]

Supporting Information

**Selective and Long-Term Stable Ammonia Electrolysis Using Pt–WO_x_ Catalysts with Suppressed NO_x_ Formation and Enhanced Activity**

*Changhyun Lim*^†^*, Hyogyun Roh*^†^*, Hyeon Kim*^†^, *Dayoung Kwon, Okkyun Seo, Jiayi Tang, Takeshi Watanabe, Wooyul Kim, Donghwa Lee^*^ and* *Kijung Yong^*^*

C. Lim, H. Roh, Prof. K. Yong

Surface Chemistry Laboratory of Electronic Materials (SCHEMA), Department of Chemical Engineering, Pohang University of Science and Technology (POSTECH), Pohang37673, South Korea; Research Center for Carbon-zero Green Ammonia Cycling, Pohang University of Science and Technology (POSTECH), Pohang 37673, Republic of Korea

E-mail: [kyong@postech.ac.kr](mailto:kyong@postech.ac.kr)

H. Kim^a^, Prof. D. Lee^a,b,c,^

**^a^** Department of Materials Science and Engineering (MSE), Pohang University of Science and Technology (POSTECH), Pohang 37673, Republic of Korea

**^b^** Division of Advanced Materials Science (AMS), Pohang University of Science and Technology (POSTECH), Pohang 37673, Republic of Korea

**^c^** Institute for Convergence Research and Education in Advanced Technology (I_CREATE), Yonsei University, Incheon 21983, Republic of Korea

E-mail: [donghwa96@postech.ac.kr](mailto:donghwa96@postech.ac.kr)

D. Kwon, Prof. W. Kim

Department of Energy Engineering, Korea Institute of Energy Technology (KENTECH), Naju 58217, South Korea

O. Seo, J. Tang, T. Watanabe

Japan Synchrotron Radiation Research Institute (JASRI), Kouto, Sayo-cho, Sayo-gun, Hyogo 679-5198, Japan

† These authors contributed equally to this work.

**Experimental Sections**

**Synthesis of WO_x_ and WO_3_ Nanowires on NF:** WO_x_ nanowires were grown by a thermal evaporation method. Nickel foam (2.5 cm $\times$ 5.0 cm) was placed on a porcelain boat filled with WO_3_ powder (2 g). For flow cell electrodes, nickel fiber (4.0 cm $\times$ 6.0 cm) was placed on a quartz boat filled with 4 g of WO_3_ powder. The boat was placed in a tube furnace under 50 mTorr. Then, the substrate and WO_3_ powder were heated at a ramping rate of 10 °C/min to 1050 °C, held at this temperature for 1 hour, and then allowed to cool naturally to room temperature. Finally, a deep blue WO_x_ (W_18_O_49_) was found on the surface of NF. The obtained product was annealed at 700 °C for 40 minutes in ambient air condition to synthesize fully oxidized WO_3_ (greenish yellow) nanowires on NF.

**Photodeposition of Pt onto WO_x_ and WO_3_ Nanowires:** Pt nanoparticles were deposited by photodeposition method using Xe lamp (Oriel 66902-500 F/1, Newport Corp., USA). WO_x_ nanowires grown on Ni foam were cut into rectangular pieces to serve as substrates for photodeposition. A precursor solution was prepared by dissolving 1 mM H_2_PtCl_6_ in a mixed solvent of deionized water (16 mL) and methanol (4 mL), followed by sonication and argon purging to ensure homogeneity and removal of dissolved oxygen. The prepared substrates were immersed in the solution and irradiated under a Xe lamp for 3 hours. After illumination, the substrates were washed with DI and dried to yield the Pt–WO_x_ (P) sample. The Pt–WO_3_ (P) sample was synthesized using the same procedure, except using WO_3_ nanowires on Ni foam as the substrate.

**Sputter Deposition of Pt on WO_x_ Nanowires:** Pt was deposited onto WO_x_ nanowires using RF magnetron sputtering (SNTEK, MSS 50). A metallic Pt target was placed on the bottom stage, while the WO_x_ nanowires grown on Ni foam were affixed to the upper holder using masking tape. During deposition, the substrate was rotated at 12 rpm under an argon atmosphere. Sputtering was conducted at a power of 50 W for two 15-minute intervals. The resulting material is referred to as the Pt–WO_x_ (S) catalyst.

**Drop-Casting of PtIr/C and Pt/C on Nickel Foam:** A commercial catalyst ink was prepared by dispersing 4 mg of PtIr/C (40 wt%) or 16 mg of Pt/C (10 wt%) in a mixture of 960 μL ethanol and 40 μL Nafion solution, followed by sonication for at least 1 hour. The ink was drop-cast onto Ni foam substrates using a micropipette, followed by drying after each application, and this process was repeated several times to achieve consistent and uniform catalyst loading. The total amount of ink was adjusted such that the loading of platinum group metals (Pt and PtIr) was fixed at 0.8 mg/cm^2^.

**Gas chromatography & Faradaic efficiency for H_2_ and N_2_ production:** The amount of hydrogen and nitrogen was quantitatively analyzed using a gas chromatograph (Agilent 7890A-5975C) equipped with a thermal conductivity detector (TCD), with argon employed as the carrier gas. The Faradaic efficiency (FE) for hydrogen and nitrogen evolution was calculated using the equation:

$$FE \left( \% \right)= \frac{n\times z \times F}{\int_{0}^{t} I \left( t \right) dt} \times100$$

where $n$ is the amount of hydrogen or nitrogen generated in moles, determined via gas chromatography; $z$ is the number of electrons required to form one mole of H₂ ($z=2$) and N_2_ ($z=6)$; F is the Faraday constant (96,485 C mol^–1^); and $\int_{0}^{t} I \left( t \right) dt$ represents the total charge passed during the electrolysis, obtained by integrating the current over time.

**Fourier Transform Infrared spectroscopy (FT-IR) for gas phase byproduct**

We employed the Nicolet iS-50 FTIR spectrometer (Thermo Fisher Scientific), equipped with a liquid nitrogen-cooled MCT-B detector, for analyzing N_2_O and NO as gaseous samples. For N_2_O, characteristic absorption bands were monitored at ~1285 cm^–1^ (ν_1_, symmetric stretch), ~2224 cm^–1^ (ν_3_, asymmetric stretch; typically spanning 2100–2250 cm^–1^), and ~589 cm^–1^ (ν_2_, bending mode). For NO, the fundamental rovibrational band (ν = 1←0) centered at ~1904 cm^–1^, with transitions distributed over ~1700–2000 cm^–1^, was analyzed.

**Quantification of NO_2_^−^**

The NO_2_^−^ concentration was measured using a standard method based on UV-Vis spectroscopy. The reacted electrolyte (1 mL), 1M HCl (1mL) and DI water (4mL) were mixed with a Griess colorimetric reagent (0.1 mL) comprising sulfanilamide, N-(1-naphthyl) ethylenediamine dihydrochloride, and phosphoric acid. The mixture was standing for 20 min, and the absorbance at 540 nm was measured by UV-vis spectrometer (Optizen POP, K LAB). To obtain a standard curve between concentration and absorbance, standard solutions of 0, 50, 100, 150 and 200 μmol of KNO_2_^−^ were prepared using the same method as the reacted electrolyte. The obtained absorbance values were then quantitatively compared with the calibration results (Figure S39) to determine the concentration of the NO_2_^−^.

**Quantification of NO_3_^−^**

The NO_3_^−^ concentration was determined using a standard method based on UV-Vis spectroscopy. The reacted electrolyte (4 mL) was mixed with 1M HCl (0.2 mL) and 0.8 wt% sulfamic acid (0.01 mL). After 15 minutes, the mixture was agitated, and the absorbances at 220 and 275 nm were measured by UV-Vis spectrometer. The total NO_3_^−^ absorbance was calculated using [Abs]_total_ = [Abs]_220 nm_ − 2 × [Abs]_275 nm_. To obtain a standard curve between concentration and absorbance, standard solutions of 0, 100, 200, 300 and 400 μmol of KNO_3_^−^ solution were prepared using the same method as the reacted electrolyte. The total absorbance values were quantitatively compared with the calibration results (Figure S40) to determine the concentration of the NO_3_^−^.

**ATR-SEIRAS Measurements:** The synthesized catalysts were carefully scissored into a fine powder to preserve their intrinsic properties. For ink preparation, 2 mg of catalyst powder was dispersed in 600 µL of isopropyl alcohol (IPA) and 30 µL of Nafion 117 solution, followed by sonication to ensure uniform dispersion. For commercial catalysts (Pt, PtIr), 1 mg of catalyst was mixed with 600 µL of IPA and 5 µL of Nafion 117 solution under the same sonication conditions. Catalyst inks were spray-coated onto a hemicylindrical silicon prism (Pike Technologies) pre-coated with electroless gold, which served as the working electrode. A Hg/HgO reference electrode (Basi, 1 M NaOH) and a Pt wire counter electrode were used to complete the three-electrode cell setup. ATR-SEIRAS experiments were conducted using a Fourier-transform infrared spectrometer (VERTEX 80v, Bruker) equipped with a variable-angle specular reflectance accessory (Veemax III, Pike Technologies) and a liquid-nitrogen-cooled mercury cadmium telluride (MCT) detector. Spectra were recorded in absorbance mode with a spectral resolution of 4 cm^−1^. Absorbance was calculated as A = −log(I/I₀), where I and I₀ represent the spectral intensities under applied potential and open-circuit conditions, respectively. Potential-dependent IR spectra were collected using linear sweep voltammetry (LSV) and multiple-step chronoamperometry (CA). LSV was performed from 0.05 V to 1.25 V at a scan rate of 20 mV/s. For CA, the potential was stepped in 0.1 V increments from 0.05 V to 1.25 V, with each potential held for 2 minutes. The final spectrum recorded with an increase of 0.2 V was presented in **Figure 4**. All experiments were repeated at least three times to confirm reproducibility and reliability.

**DFT Calculation:** Density-functional-theory (DFT) calculations were performed with the Vienna Ab-initio Simulation Package (VASP).^[1]^ The projector-augmented-wave (PAW) method, together with the Perdew–Burke–Ernzerhof generalized-gradient-approximation (PBE-GGA) exchange–correlation functional, was used to describe electron–ion interactions.^[2–4]^ A plane-wave cutoff energy of 450 eV was used for the plane wave representation of the wavefunctions, and electronic and ionic structures were relaxed with an energy threshold of 10^–6^ eV and a force threshold of 10^–2^ eV/Å for our calculations.

The bulk structures of monoclinic W_18_O_49_(=WO_x_) (space group P2/m), monoclinic WO_3_ (P21/n), and cubic Pt (Fm‒3m) are optimized with the Brillouin zone sampled by Monkhorst-Pack scheme with $2\times9\times3$, $5\times5\times5$, and 9$\times9\times9$ k-point grid, respectively. The optimized lattice parameters are in good agreement with available experimental values (*Figure* *S28*).^[5–7]^

The slab structures of WO_x_, WO_3_, and Pt are constructed by following sequence. High-resolution TEM analysis revealed the preferential growth orientation of [010] for WO_x_ support, [200] for WO_3_ support, and [100] for Pt nanoparticles (**Figures 1e**, **1f** and *S9*). Accordingly, the exposed surfaces were selected as WO_x_ (100) and WO_3_ (001), which are perpendicular facets to [010] and [200] growth orientations, and Pt (100), which is parallel facet to [200] growth orientation. Because the WO_x_ (100) and WO_3_ (001) facets can exhibit several possible terminations, the most stable surface termination is identified. For WO_x_ (100), the six terminations of WO_2_, O, W_2_O_3_, W_2_O_7_, WO, and WO_6_ were compared, and WO_2_ termination is identified as the most stable termination under W-rich condition (*Figure* *S29a*). For WO_3_ (001), the three terminations of O_0.5_, O, and WO were evaluated and the O_0.5_ termination is identified as the most stable termination under O-rich condition (*Figure* *S29b*). These terminations are consistent with previous theoretical studies.^[8–12]^ All slab structures were symmetrized and separated by a vacuum region greater than 20 Å along the z‐direction to eliminate interactions between periodic images.

The resulting slab structures of WO_2_-terminated WO_x_ (100), O_0.5_-terminated WO_3_ (001), Pt (100) are shown in *Figure* S30. For WO_2_-terminated WO_x_ (100), a $3\times1$ supercell with a thickness of 11 layers slab was constructed; the bottom 5 layers were fixed during the geometric optimization, and the Brillouin zone was sampled with $3\times3\times1$ k-point grid (*Figure* *S30a*). For O_0.5_-terminated WO_3_ (001), a 7 layered $(2\times2)$ slab was constructed; the bottom 3 layers are fixed, and $3\times3\times1$k-point grid was applied (*Figure* *S30b*). For Pt (100), a 7 layered$(3/\surd2 \times3/\surd2)$ supercell slab was employed; the bottom 3 layers were fixed, and a 4 × 4 × 1 k-point grid was used (*Figure* *S30c*).

The interface structures of Pt–WO_x_ and Pt–WO_3_ were constructed by minimizing the lattice mismatches between the Pt lattice and the WO_x_ or WO_3_ support lattices. Since the Pt nanoparticles are relatively lower than WO_x_ or WO_3_ support thickness, Pt (100) lattice was fitted for WO_x_ (100) or WO_3_ (001) lattices. In doing so, the Pt lattice was positioned at the most stable Pt adsorption site on WO_x_ (100) or WO_3_ (001), as identified in *Figure S31*. The lattice mismatches are calculated by the following equations:

$$\delta_{a}=\frac{a_{sup}-na_{Pt}}{a_{sup}}, \delta_{b}=\frac{b_{sup}-mb_{Pt}}{b_{sup}} (sup=WO_{x} or WO_{3})$$

which n and m are the repeat numbers of Pt (100) unit cell along the a and b directions, respectively. Positive δ indicates tensile strain, whereas negative δ indicates compressive strain in the Pt lattice. For Pt–WO_x_, n=4 and m=5 minimize the lattice mismatch with δ_a_ = 1.96 % and δ_b_ = 0.83 %; therefore, a 4 × 5 supercell of three-layer Pt (100) was placed on the WO_x_ (100) surface (*Figure* *S32a*). For Pt–WO₃, n=m=4 minimizes the lattice mismatch with δ_a_= δ_b_=-5.06%; accordingly, a 4 × 4 three-layer Pt (100) supercell was placed on the WO_3_ (001) surface (*Figure* *S32b*). All Pt layers were fully relaxed in both in Pt–WO_x_ and Pt–WO_3_ interface structures.

A computational hydrogen electrode (CHE) was used to estimate the adsorption energies of NH_x_ dehydrates to avoid direct calculations of charged system.^[13]^ The Gibbs free energy of the NH_x_ dehydrates adsorption is computed as following Equations:

$$\Delta G_{*NH_{3}+*OH} = G_{*NH_{3}+*OH} - G_{*OH}-\frac{1}{2}\left( G_{N_{2}} -{3G}_{H_{2}} \right)$$

$$\Delta G_{*NH_{x}+*OH} = G_{*NH_{x}+*OH} - G_{*NH_{x+1}+*OH}+\frac{1}{2}G_{H_{2}} (x=0,1,and 2)$$

$$G = E + ZPE - T\Delta S$$

Where $G_{*NH_{x}+*OH}$ is the Gibbs free energy of NH_x_ dehydrates adsorbed surface, $G_{N_{2}}$ is the Gibbs free energy of N_2_ gas at 1atm, $G_{H_{2}}$ is the Gibbs free energy of H_2_ gas at 1atm, U_RHE_ is the electrode potential referenced to the reversible hydrogen electrode (RHE), E is the DFT calculated energy, ZPE and TΔS are the zero-point energy and entropy at 298K each. When calculating the vibrational frequencies of an adsorbed system, only the adsorbed molecules and combined atom(s) at the surface were considered. Bader charge analysis has been carried out using a method developed by the Henkelman group.^[14]^

*
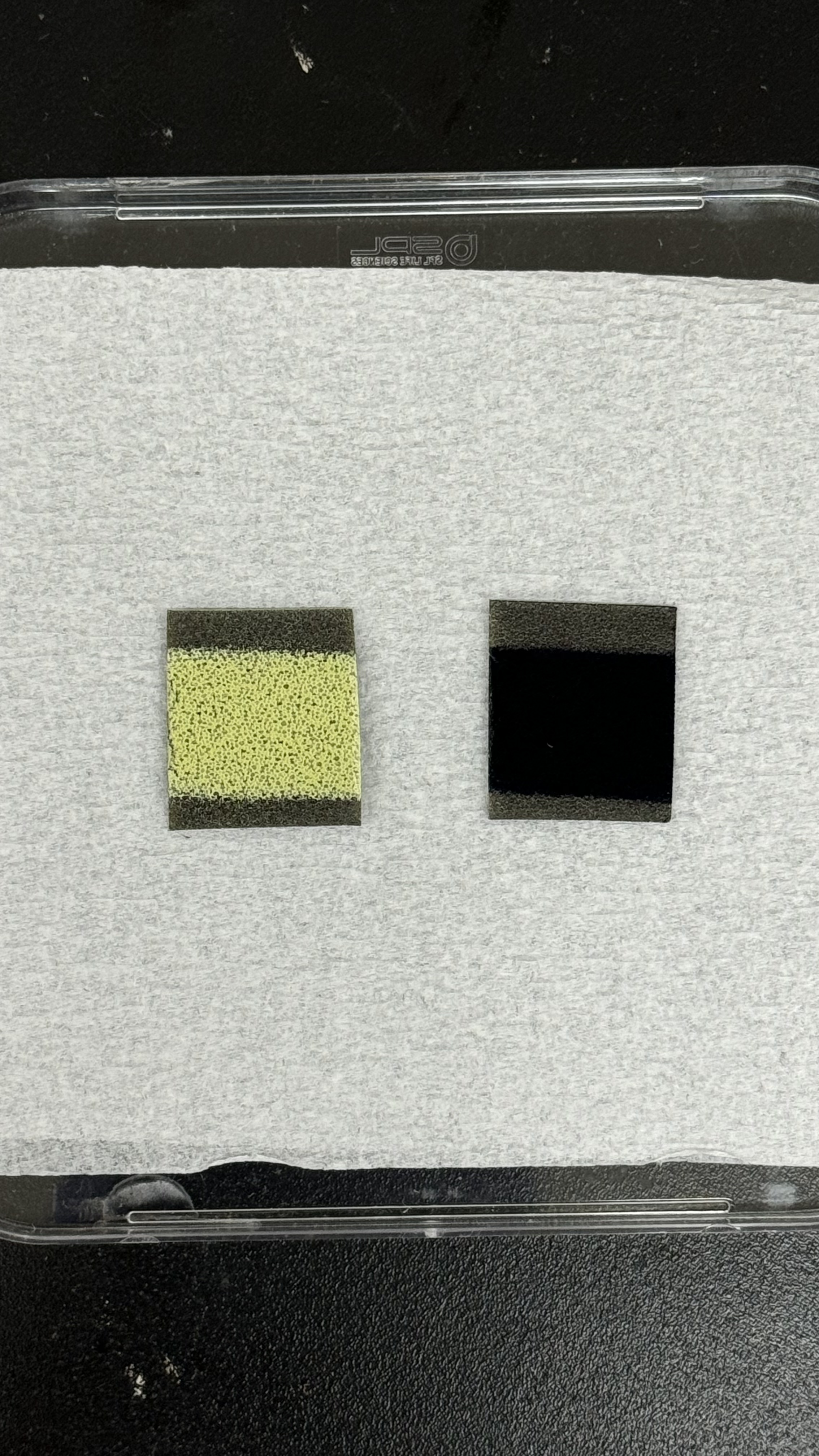
*

**Figure S1.** Photograph of the dark bluish WO_x_ nanowires on Ni foam.

*
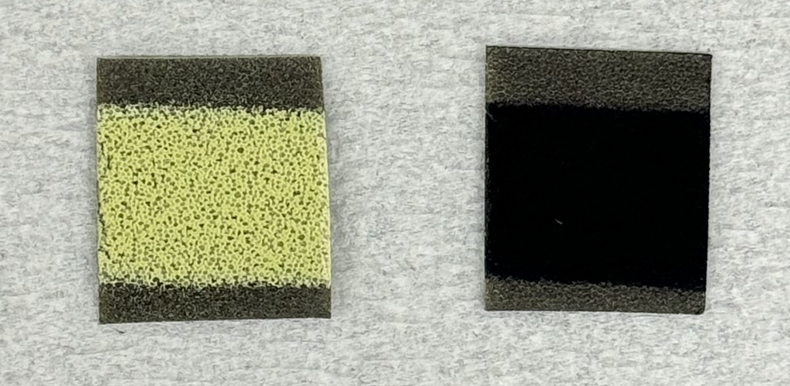
*

**Figure S2.** Photograph of the greenish yellow WO_3_ nanowires on Ni foam.


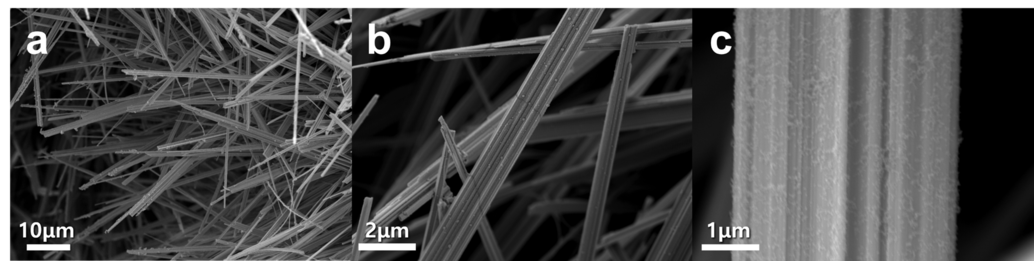


**Figure S3.** Field emission scanning electron microscopy (FESEM) images of Pt–WO_x_ (P) in various magnification.


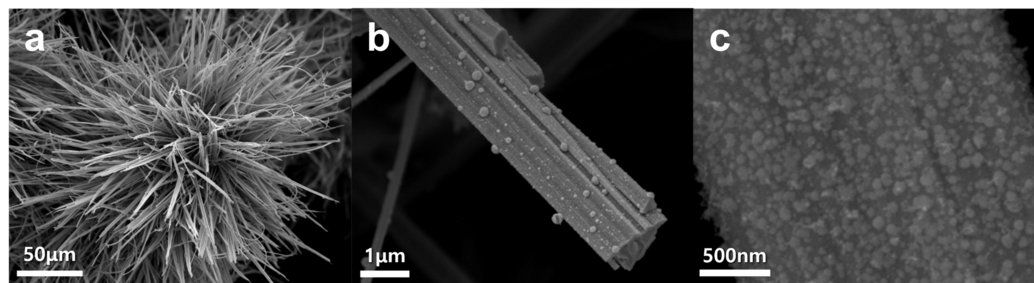


**Figure S4.** FESEM images of Pt–WO_3_ (P) in various magnification.


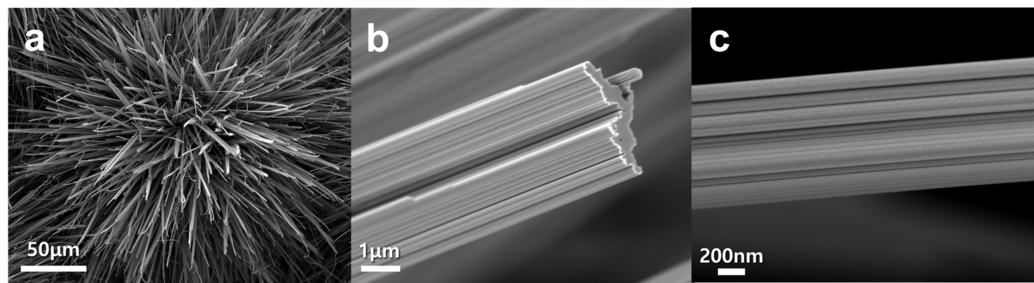


**Figure S5.** FESEM images of Pt–WO_x_ (S) in various magnification.


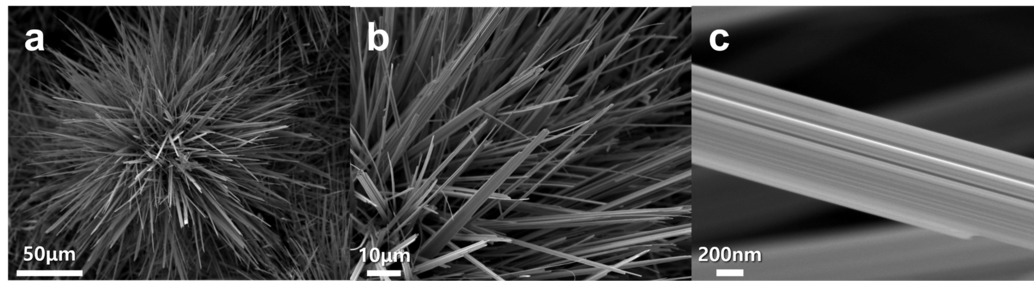


**Figure S6.** FESEM images of WO_x_ NWs in various magnification.


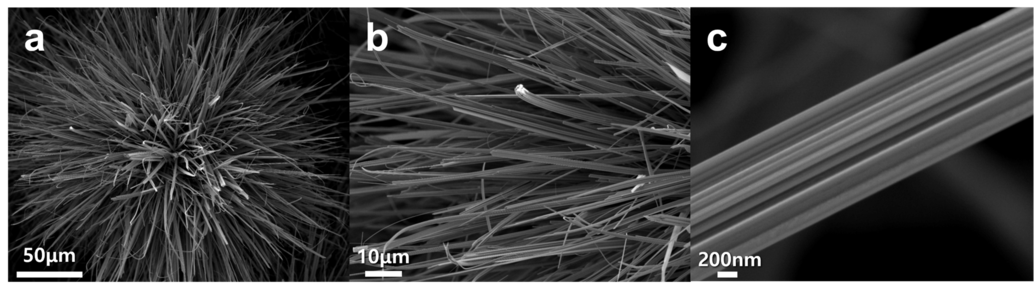


**Figure S7.** FESEM images of WO_3_ NWs in various magnification.


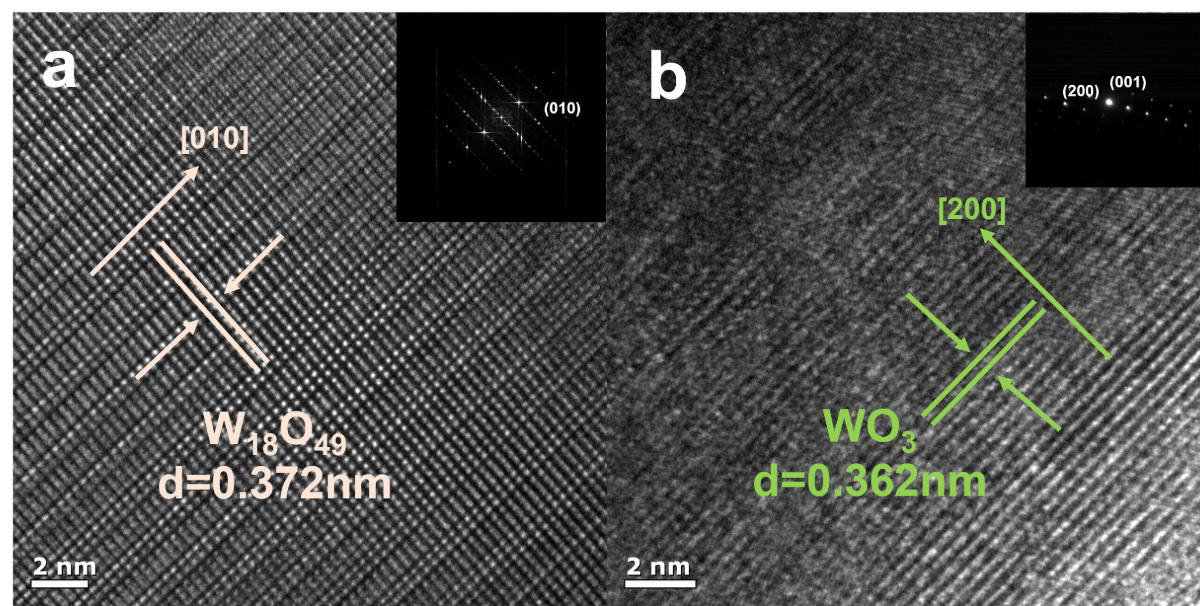


**Figure S8.** HRTEM images and corresponding FFT patterns of a) W_18_O_49_ and b) WO_3_.


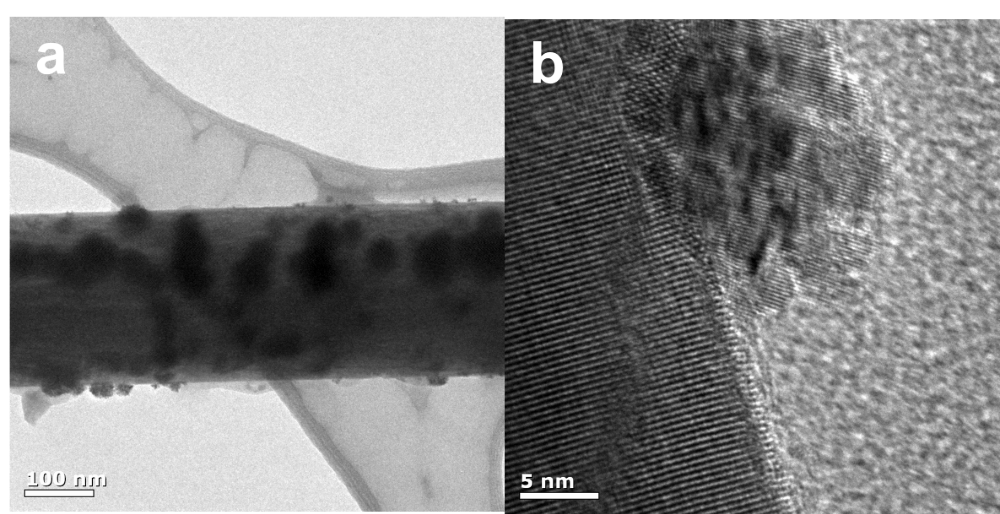


**Figure S9.** a) Low-resolution and b) high-resolution TEM images of Pt–WO_3_ (P).


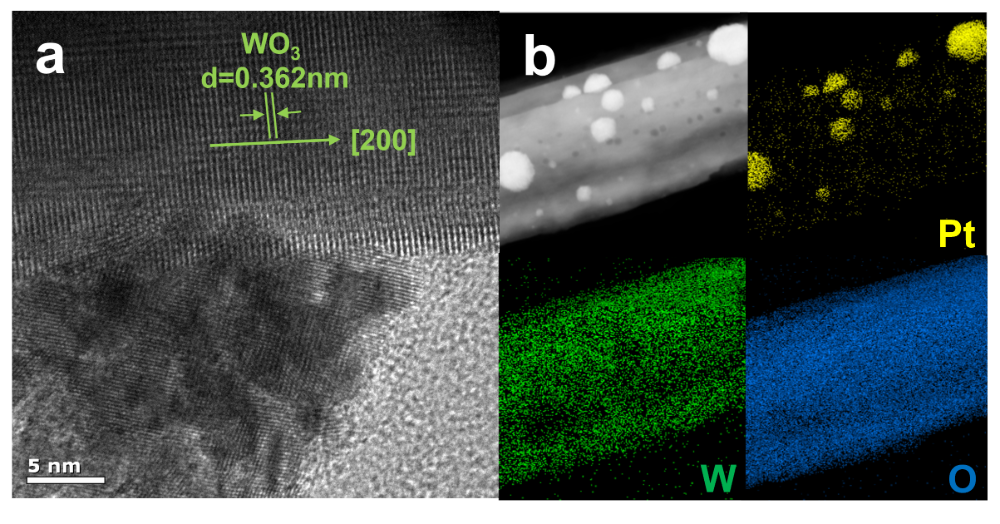


**Figure S10.** a) HRTEM image and b) HAADF-STEM image and corresponding EDS elemental mappings (Pt, W, O) of Pt–WO_3_ (P).


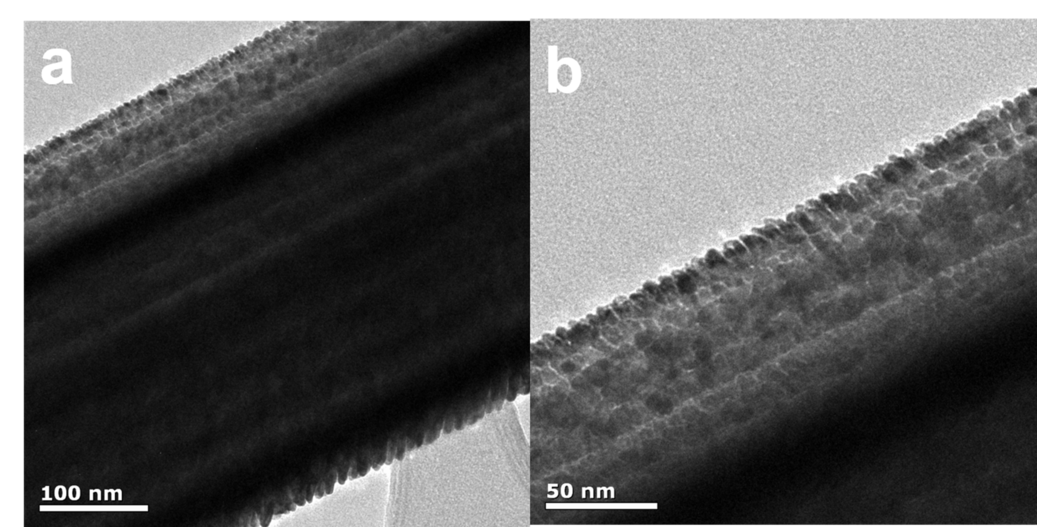


**Figure S11.** a) Low-resolution and b) high-resolution TEM images of Pt–WO_x_ (S).


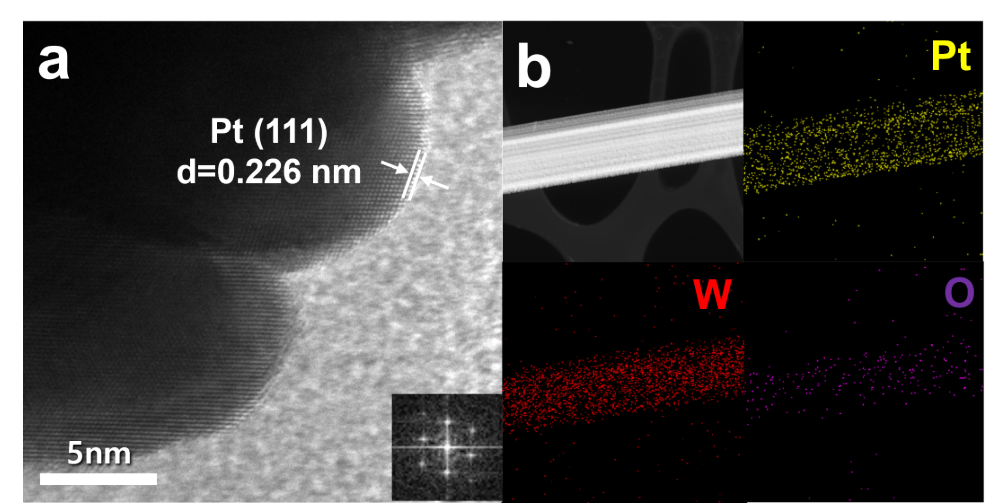


**Figure S12.** a) HRTEM image and b) HAADF-STEM image and corresponding EDS elemental mappings (Pt, W, O) of Pt–WO_x_ (S).


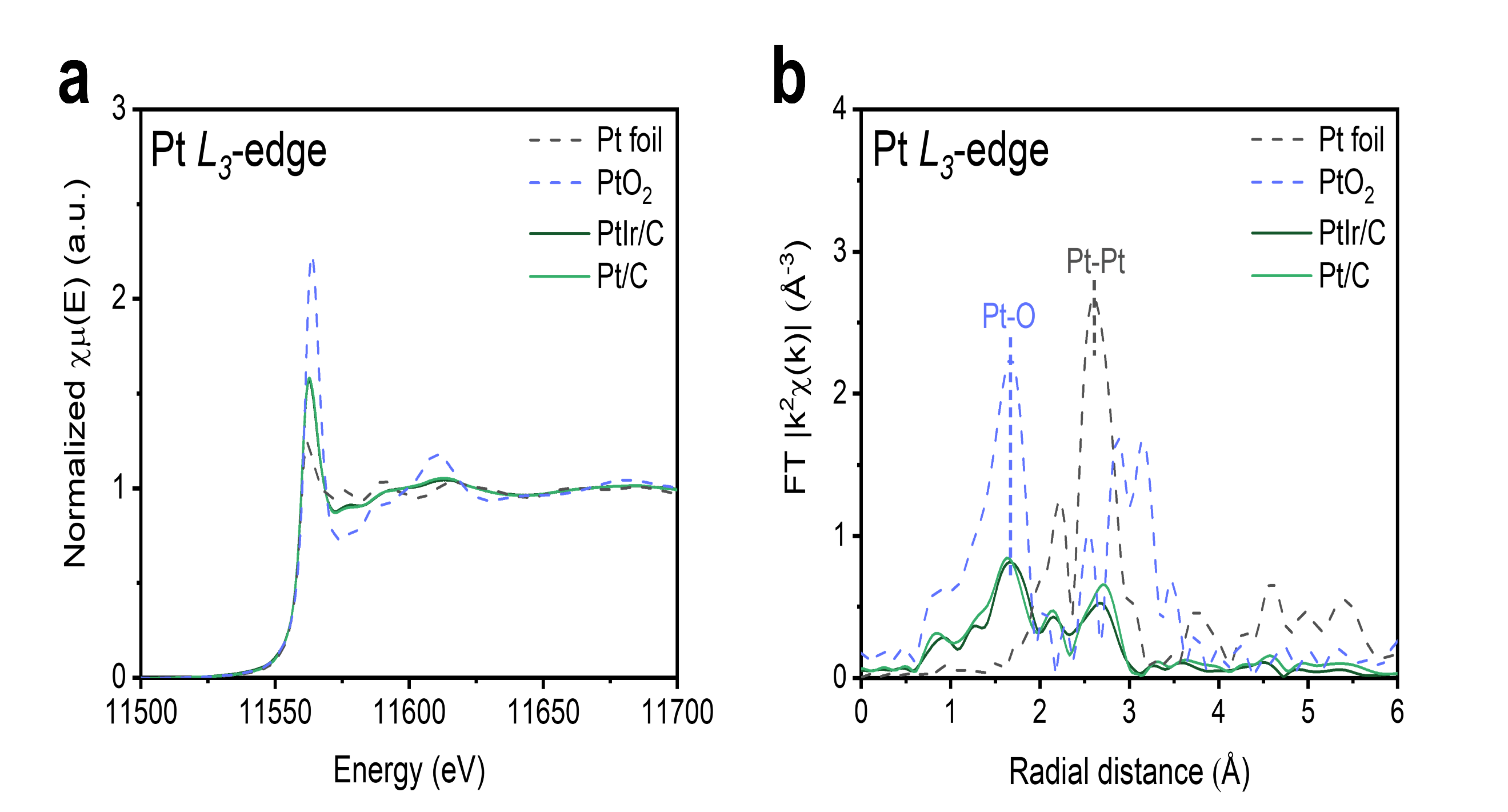


**Figure S13.** a) Normalized XANES spectra b) FT-EXAFS spectra of Pt foil, PtO_2_, PtIr/C, and Pt/C.


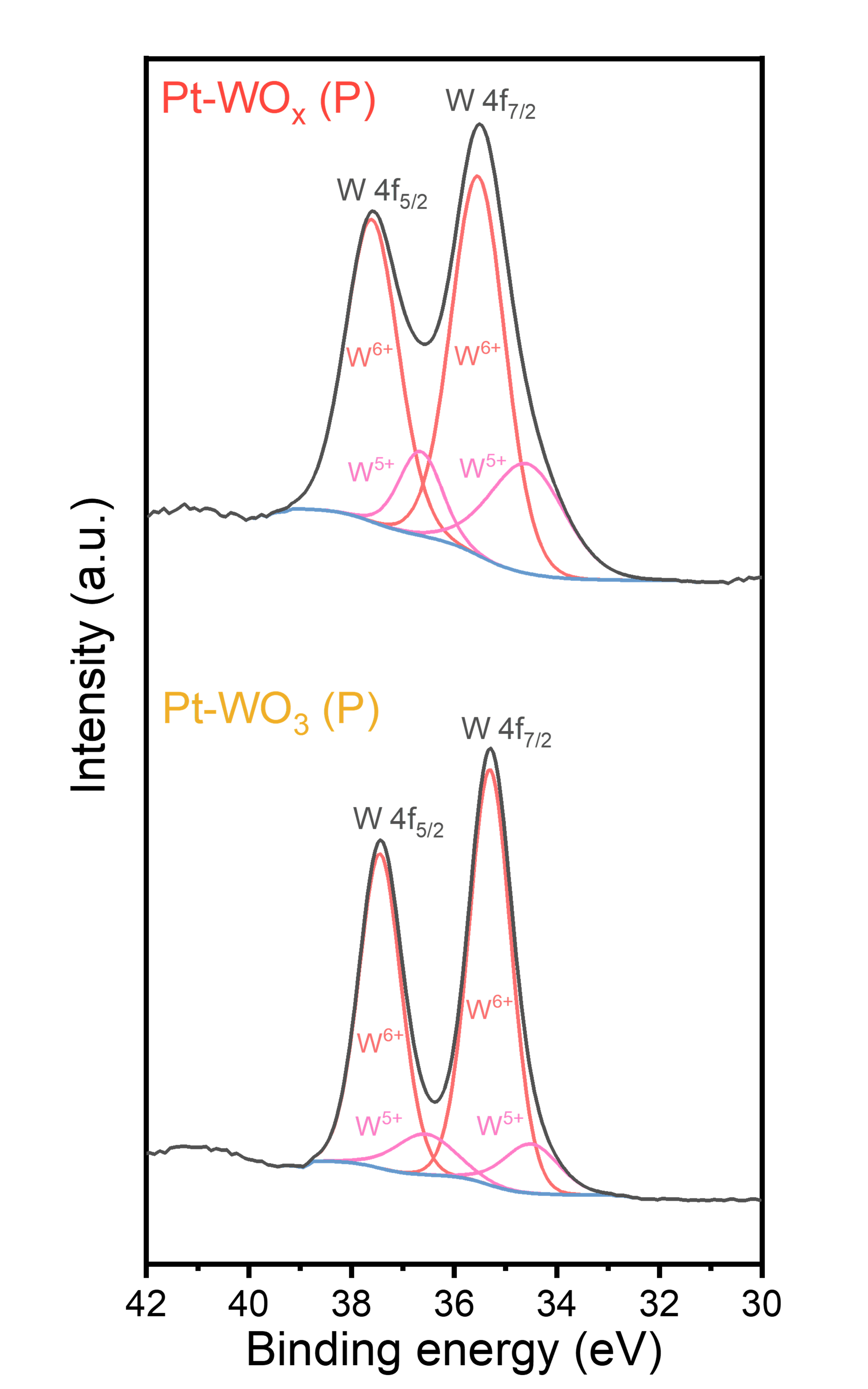


**Figure S14.** W 4f XPS of Pt–WO_x_ (P) and Pt–WO_3_ (P).


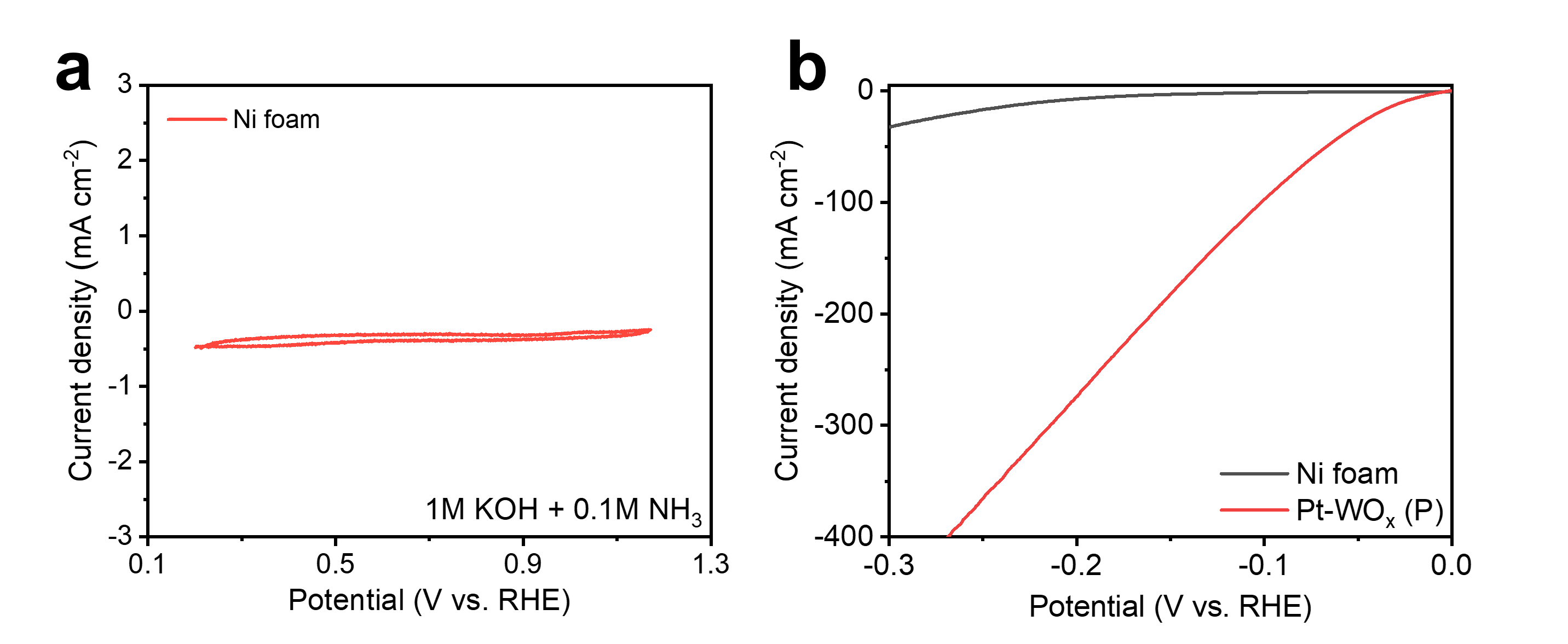


**Figure S15.** a) CV curve of Ni foam in 1 M KOH + 0.1 M NH_3_ and b) polarization curves of Ni foam and Pt-WO_x_ (P) on NF in 1 M KOH at a scan rate of 5 mV s^–1^ without iR correction.


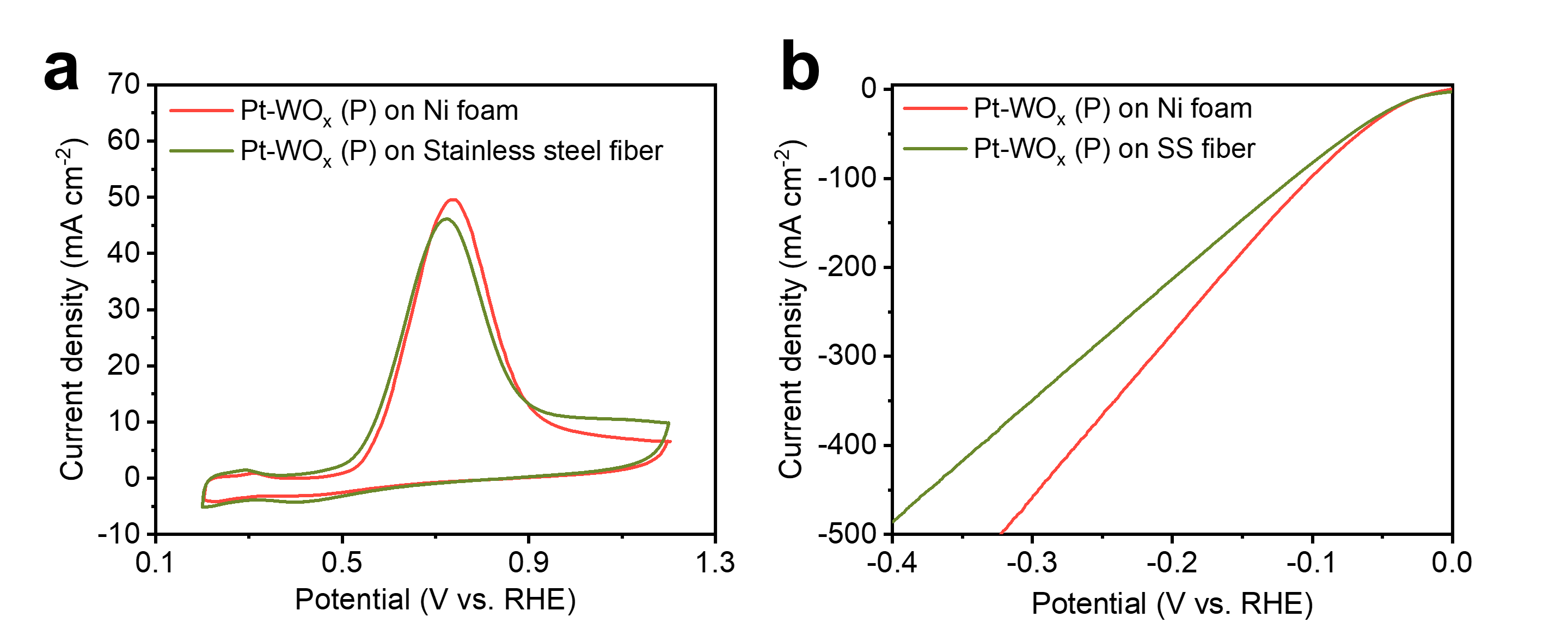


**Figure S16.** a) CV curve of Pt-WO_x_ (P) on NF and stainless-steel fiber in 1 M KOH + 0.1 M NH_3_ and b) polarization curves of Pt-WO_x_ (P) on NF and stainless-steel fiber in 1 M KOH at a scan rate of 5 mV s^–1^ without iR correction.

**Table S1.** Determination of Pt and W contents (Wt%) of Pt–WO_x_ (P), Pt–WO_3_ (P) and Pt–WO_x_ (S) via inductively coupled plasma optical emission spectroscopy (ICP-OES).

| **Catalyst** | **Pt content [Wt%]** | **W content [Wt %]** |
| --- | --- | --- |
| Pt–WO_x_ (P) | 1.38 | 27.97 |
| Pt–WO_3_ (P) | 1.65 | 25.65 |
| Pt–WO_x_ (S) | 0.80 | 26.67 |


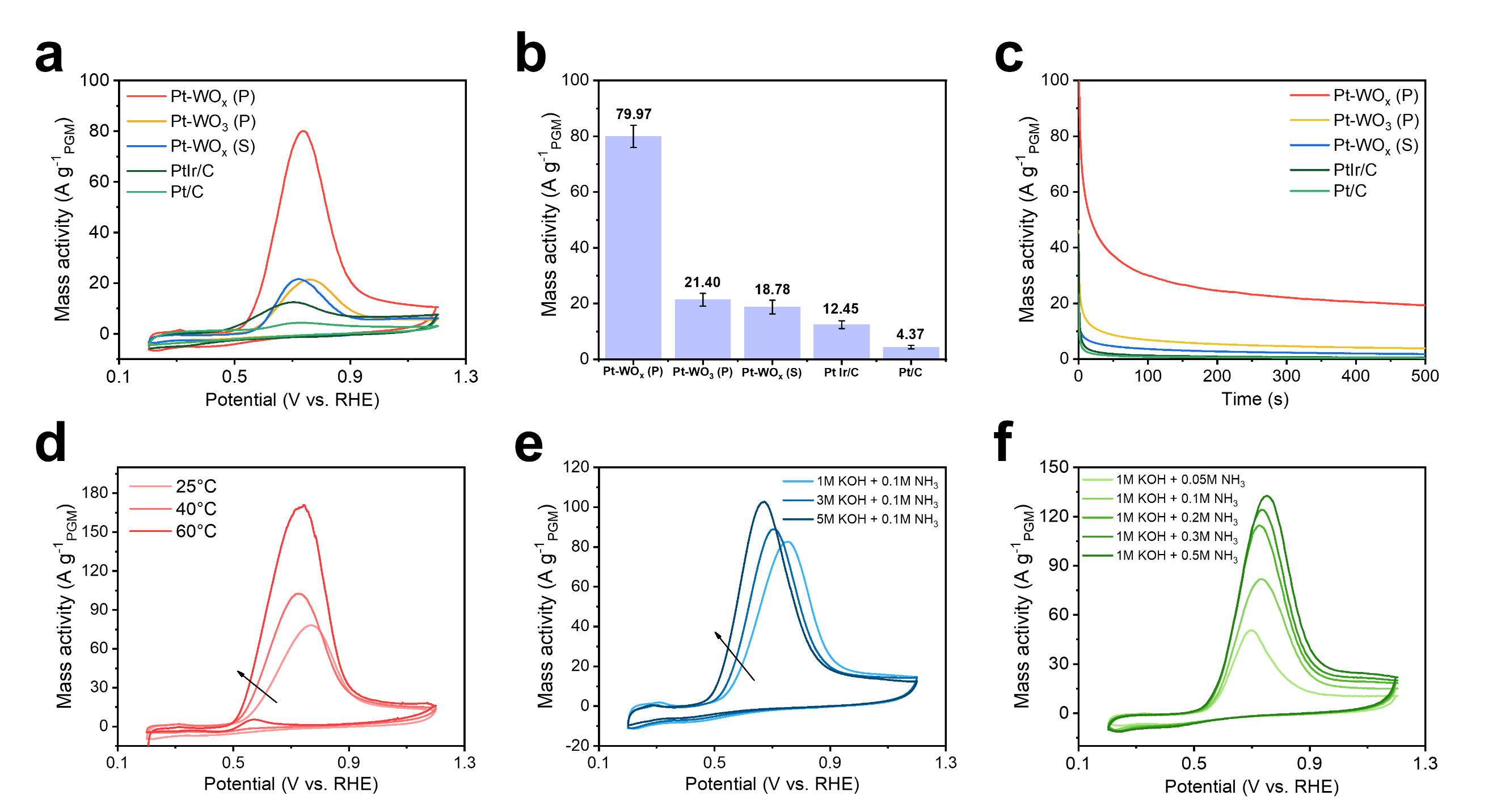


**Figure S17.** a) Mass activity for different catalysts and b) comparison diagram in Ar-saturated 1.0 M KOH + 0.1 M NH_3_ at scan rate of 5 mV s^–1^. c) CA profiles for 500 s in Ar-saturated 1.0 M KOH + 0.1 M NH_3_. Mass activity under varying d) different temperatures, e) concentration of KOH and f) NH_3_.


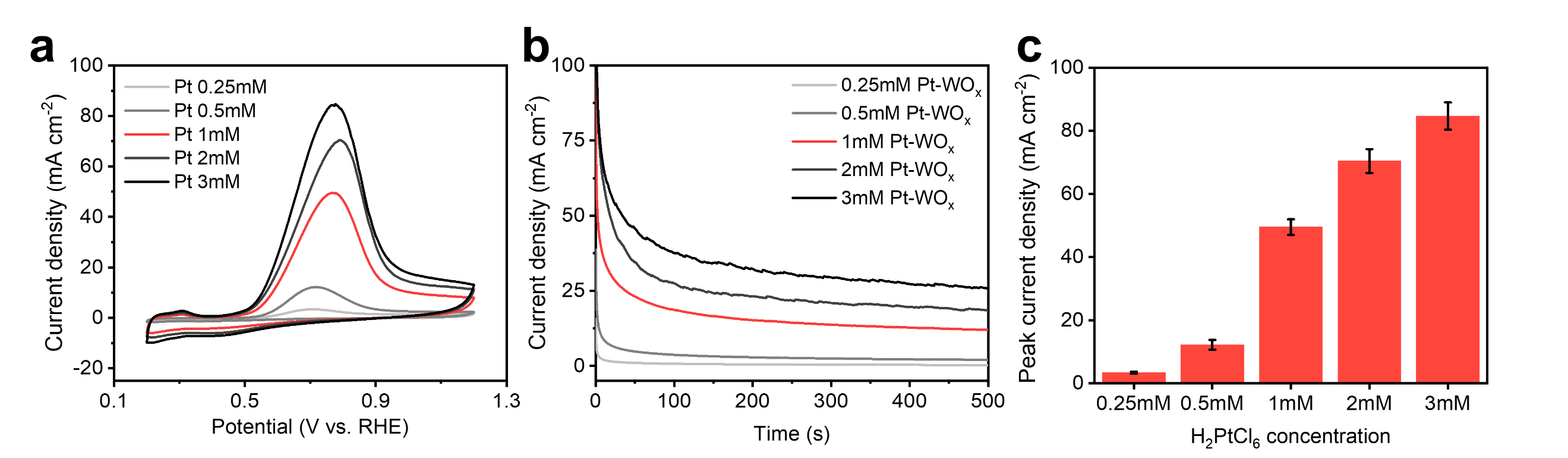


**Figure S18.** a) CV curves, b) CA profiles in 1 M KOH + 0.1 M NH_3_ with different concentration of H_2_PtCl_6_ during photodeposition and c) corresponding peak current density index.


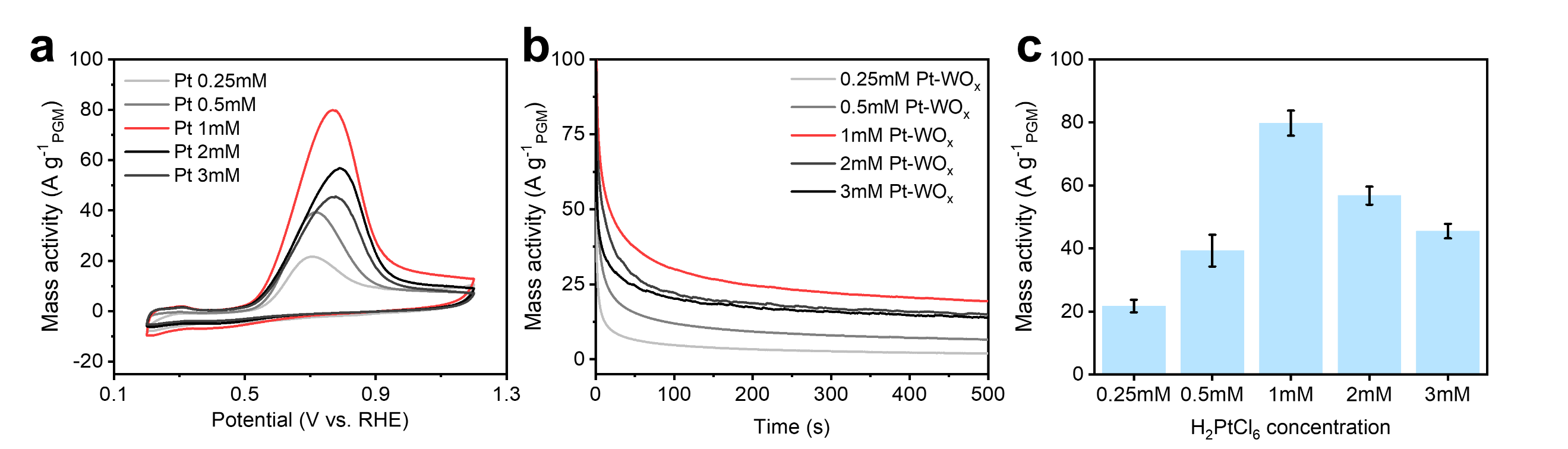


**Figure S19.** a) CV curves, b) CA profiles in 1 M KOH + 0.1 M NH_3_ with different concentration of H_2_PtCl_6_ during photodeposition and c) corresponding mass activity index.


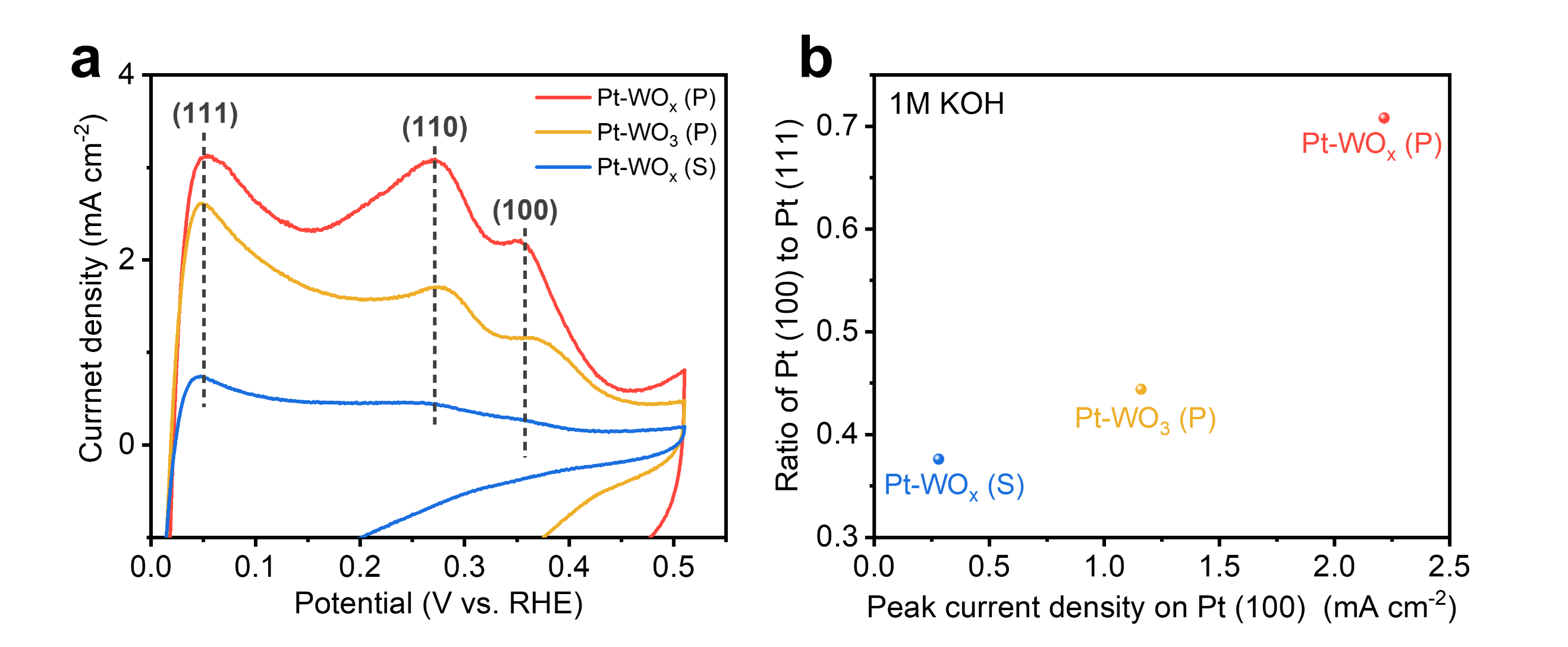


**Figure S20.** a) CV curves in 1 M KOH between 0 to 0.5 V vs. RHE at scan rate of 20 mV s^–1^. b) Correlation between adsorbate charge on the Pt (100) facet and current density on Pt (100) relative to Pt (111).


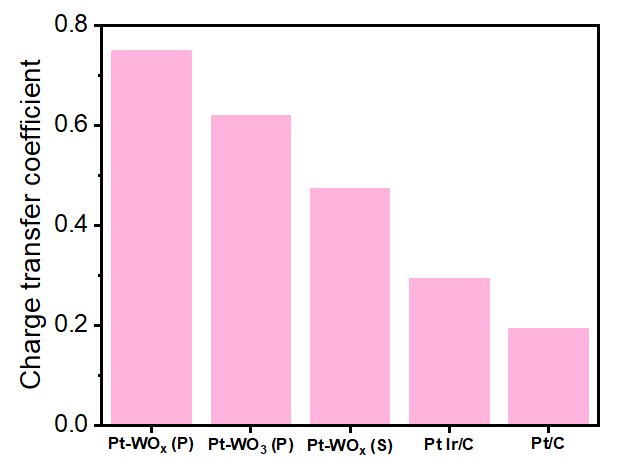


**Figure S21.** Charge transfer coefficients of Pt–WO_x_ (P), Pt–WO_3_ (P), Pt–WO_x_ (S), PtIr/C and Pt/C.


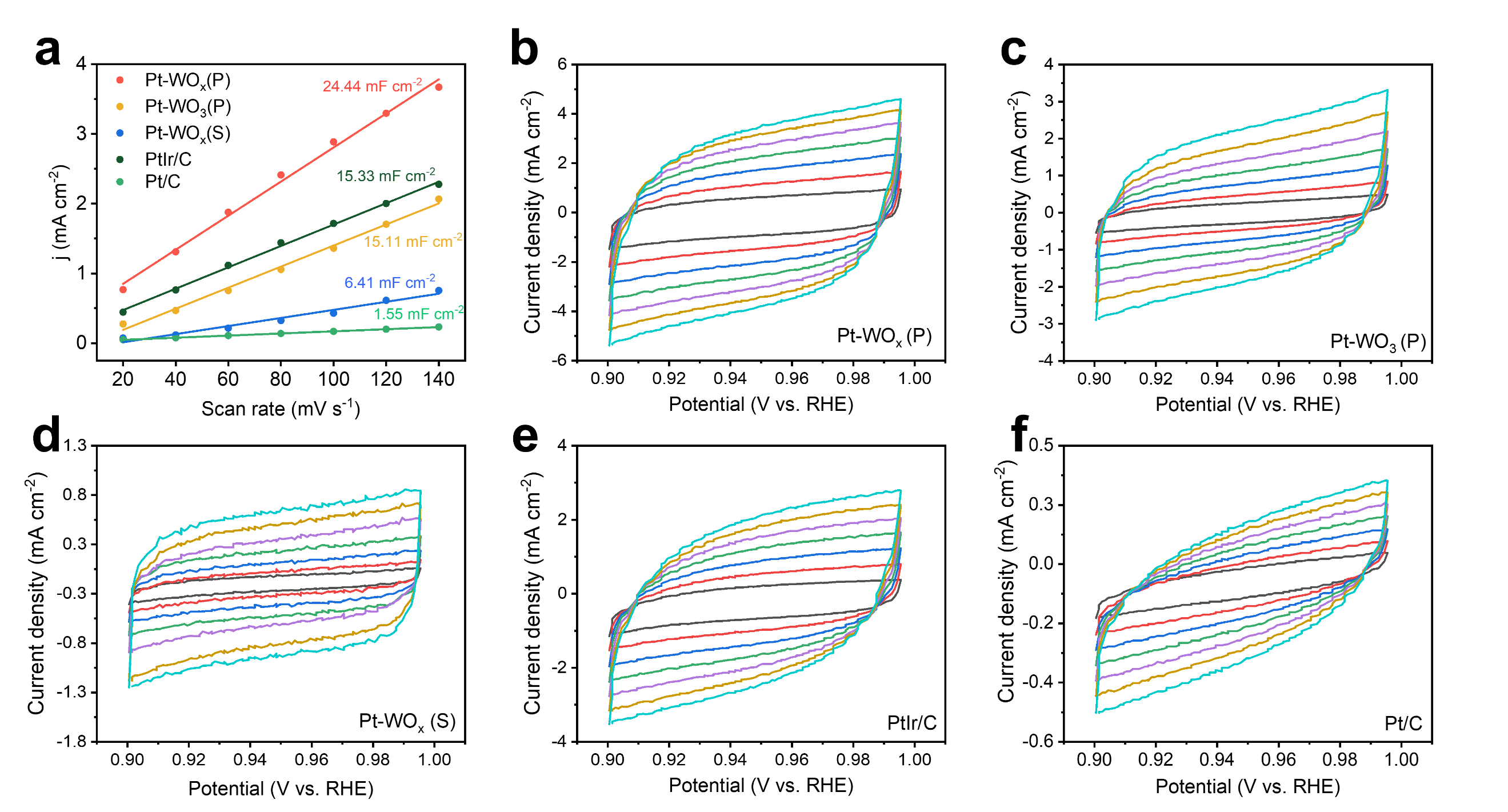


**Figure S22.** a) Electrochemical double-layer capacity (C_dl_) of different catalysts and CV curves of b) Pt–WO_x_ (P), c) Pt–WO_3_ (P), d) Pt–WO_x_ (S), e) PtIr/C, and f) Pt/C.


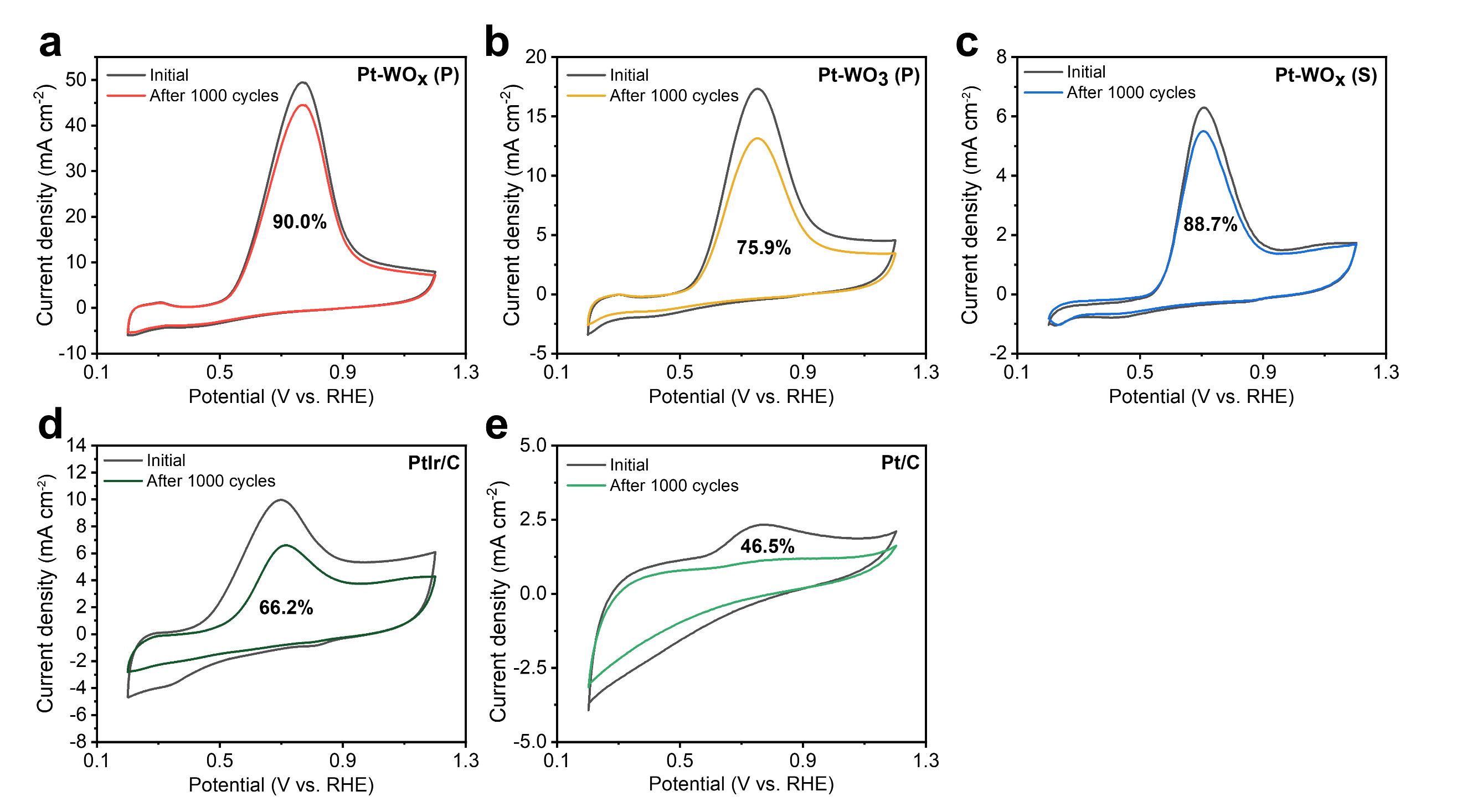


**Figure S23.** Stability test for AOR. CV curves of a) Pt–WO_x_ (P), b) Pt–WO_3_ (P), c) Pt–WO_x_ (S), d) PtIr/C, and e) Pt/C between 0.2 V to 1.2 V vs. RHE in 1 M KOH + 0.1 M NH_3_ before and after 1000 cycles of ammonia oxidation.


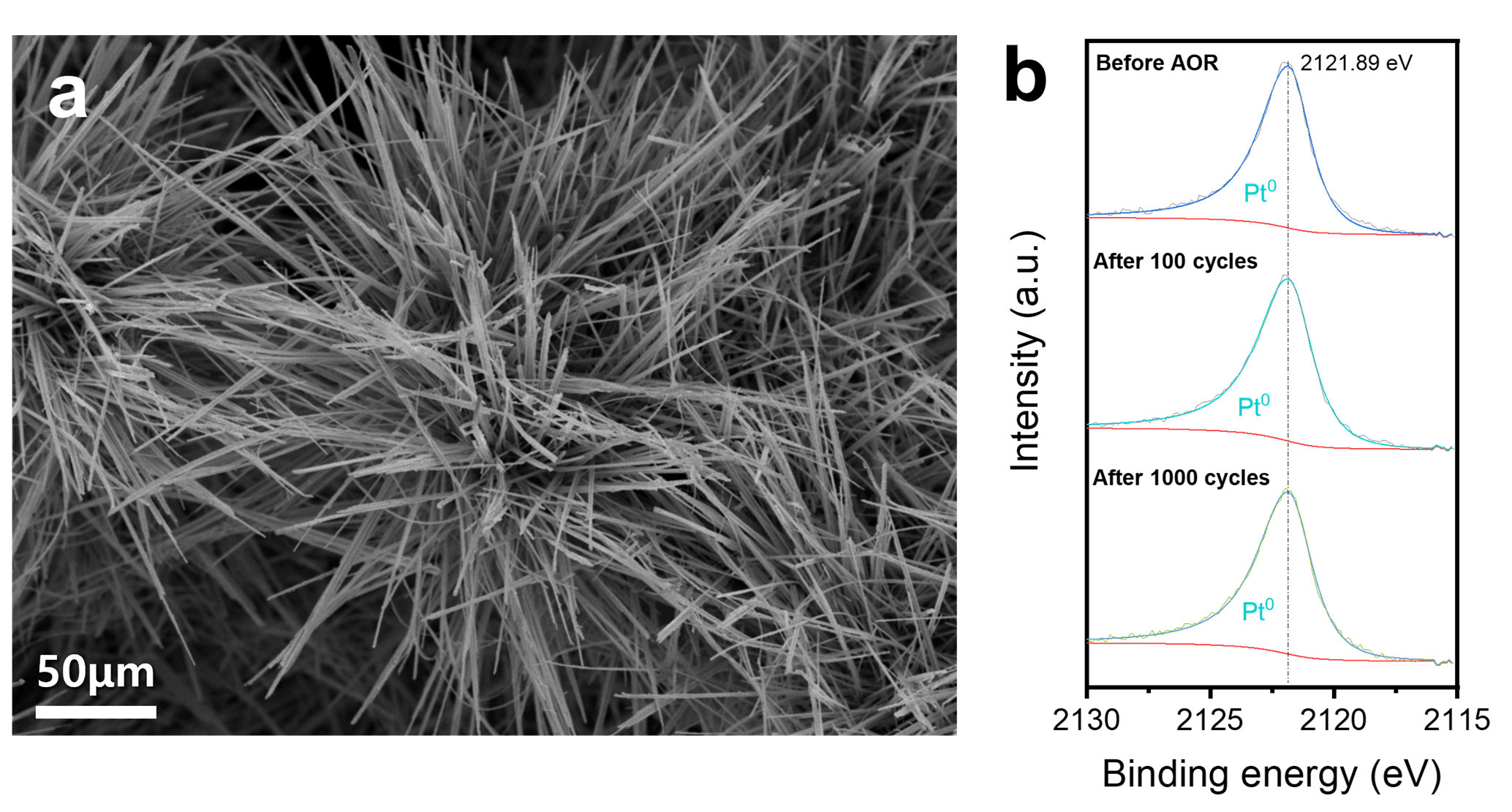
 **Figure S24.** a) SEM image of Pt–WO_x_ (P) after 1000 cycles of ammonia oxidation. XPS analysis of Pt 4d orbital before and after 100 and 1000 cycles of ammonia oxidation with Pt–WO_x_ (P).


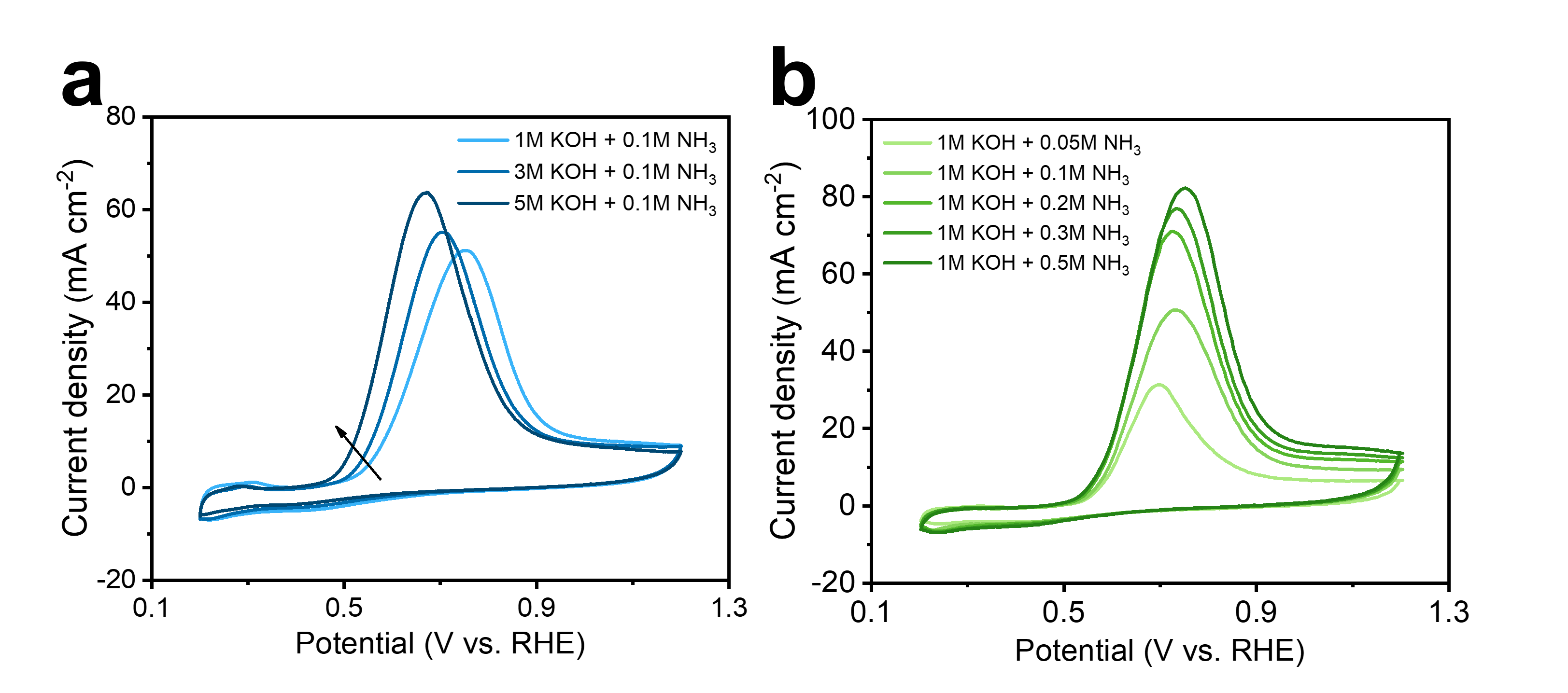
**Figure S25.** a) CV curves of Pt–WO_x_ (P) with different concentration of a) KOH and b) NH_3_.


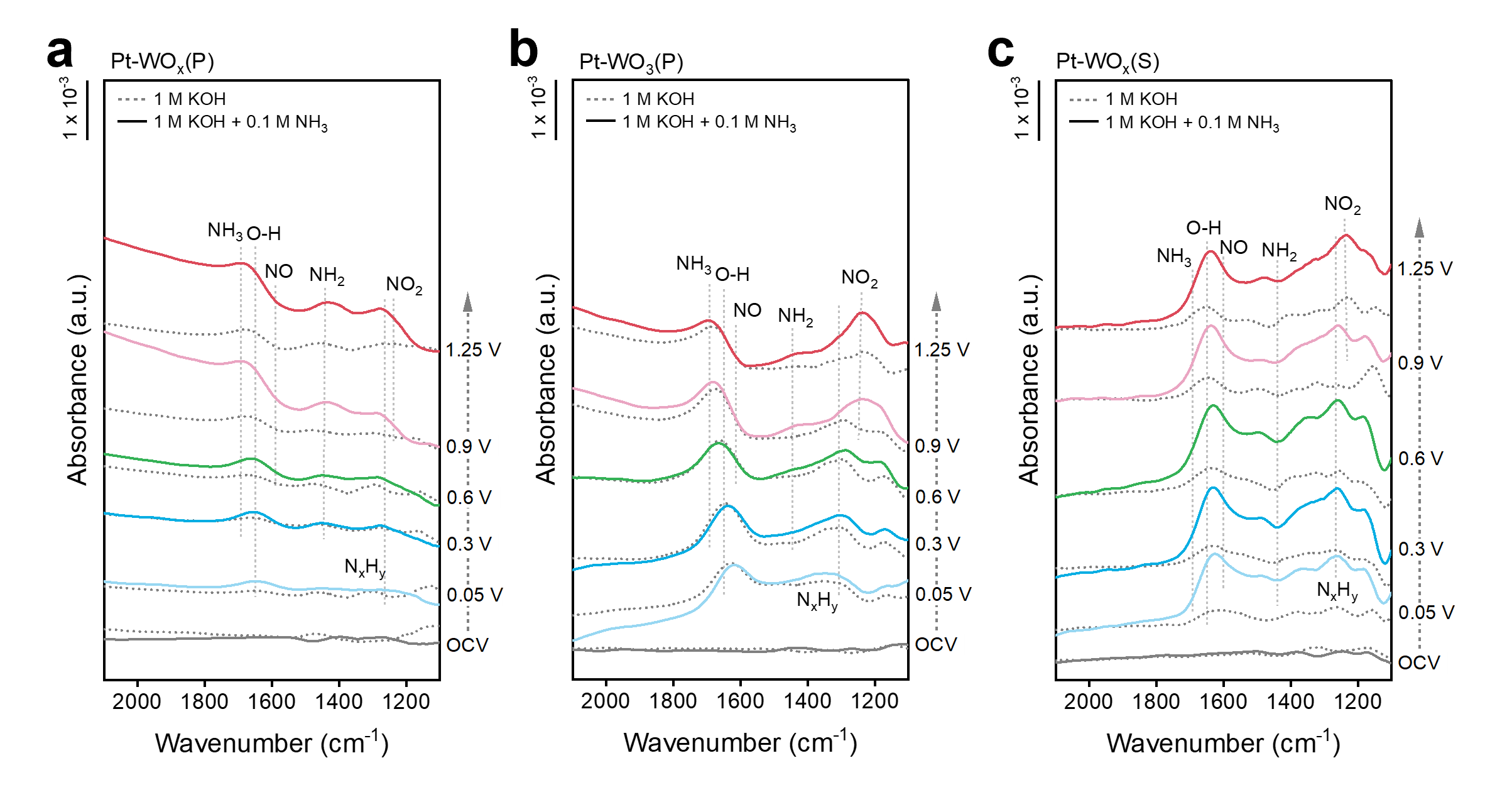
**Figure S26.** ATR-SEIRA spectra acquired during LSV from 0.05 to 1.25 V (vs. RHE) in 1 M KOH (dotted) and 1 M KOH + 0.1 M NH_3_ electrolyte (colored) with a scan rate of 0.02 V s^–1^ on a) Pt–WO_x_ (P), b) Pt–WO_3_ (P), and c) Pt–WO_x_ (S) in the 2100–1150 cm^–1^ range. The reference spectrum was collected at open-circuit potential (OCV).


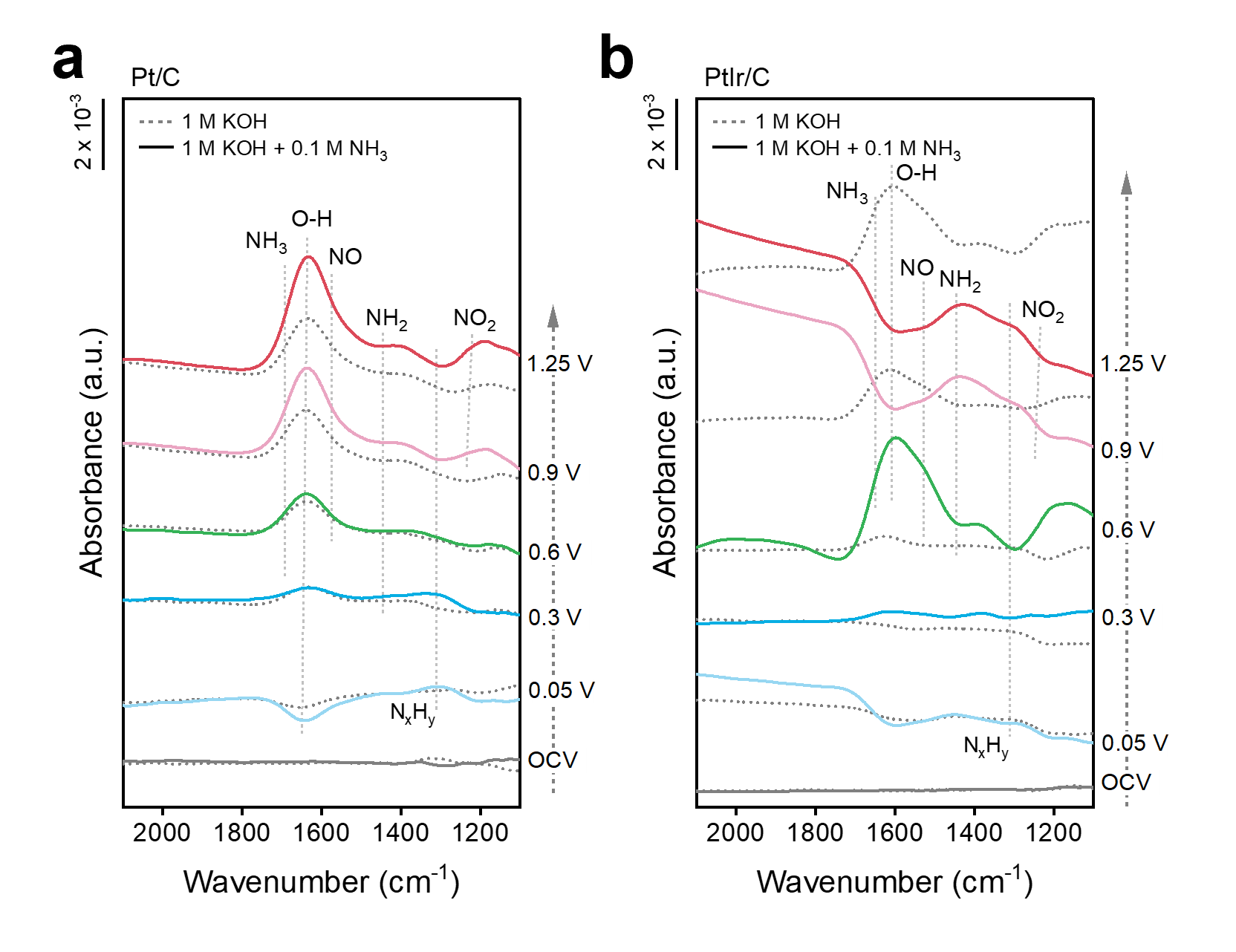


**Figure S27.** ATR-SEIRA spectra acquired during LSV from 0.05 to 1.25 V (vs. RHE) in 1 M KOH (dotted) and 1 M KOH + 0.1 M NH_3_ electrolyte (colored) with a scan rate of 0.02 V s^–1^ on a) Pt/C and b) PtIr/C in the 2100–1150 cm^–1^ range. The reference spectrum was collected at open-circuit potential (OCV).

**
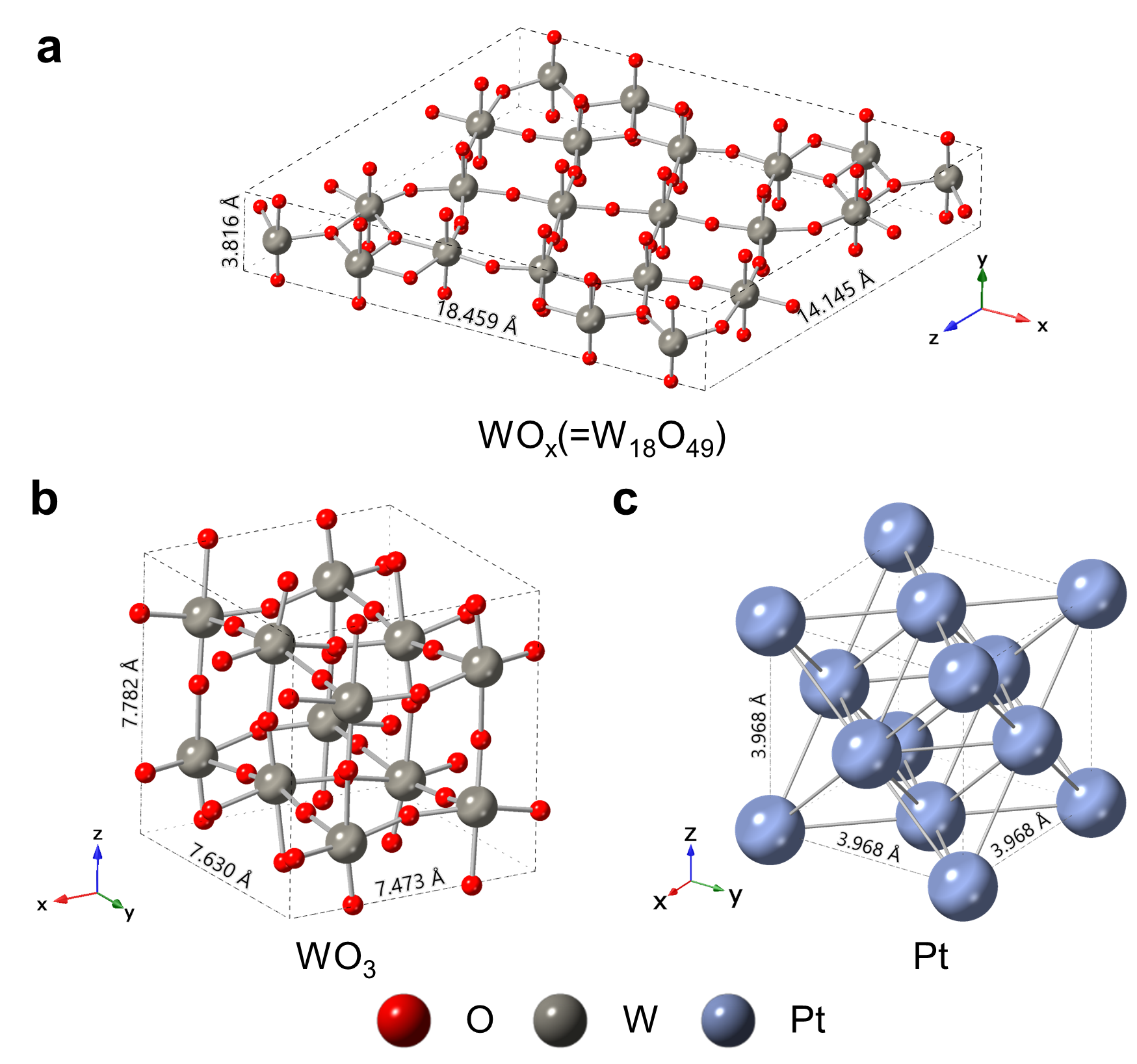
 Figure S28.** Relaxed bulk structures of a) monoclinic WO_x_(=W_18_O_49_) b) monoclinic WO_3_ and c) cubic Pt.


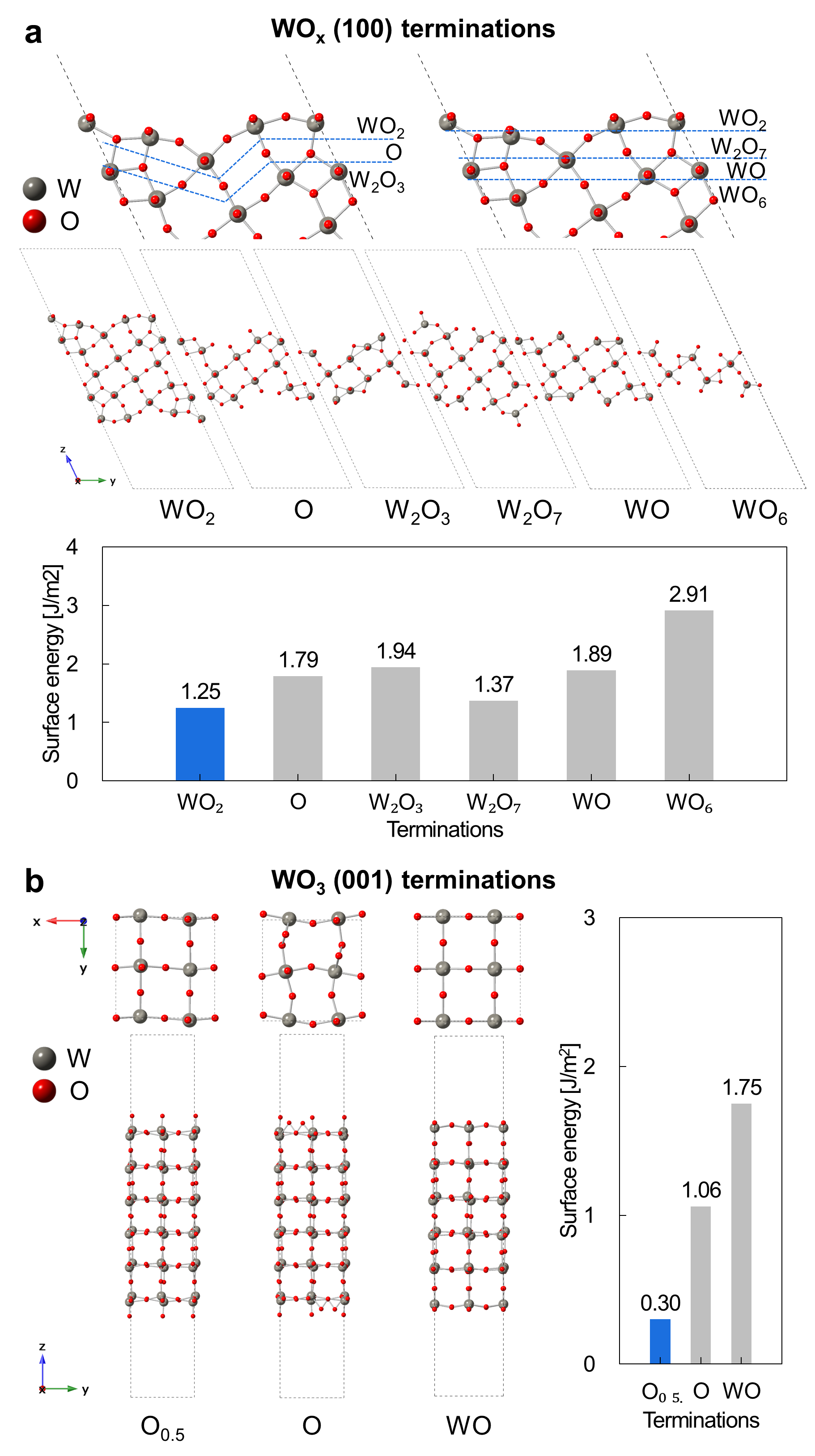


**Figure S29.** Different terminations and calculated surface energies of a) WO_x_ (100) b) WO_3_ (001) slab structures.


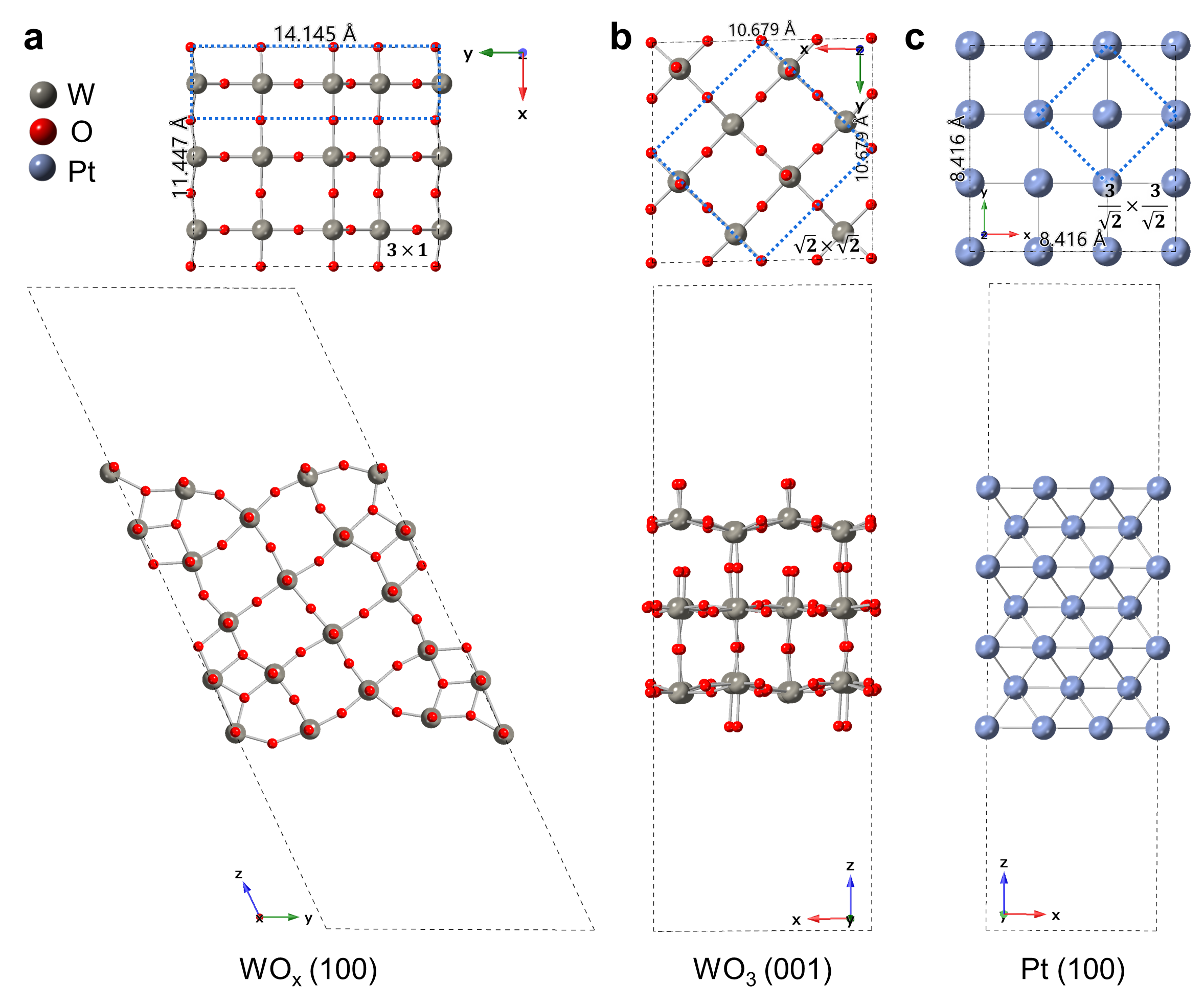


**Figure S30.** Relaxed slab structures of a) WO_x_ (100) b) WO_3_ (001) and c) Pt (100). Blue dash rectangle is the unit cell area of each system.


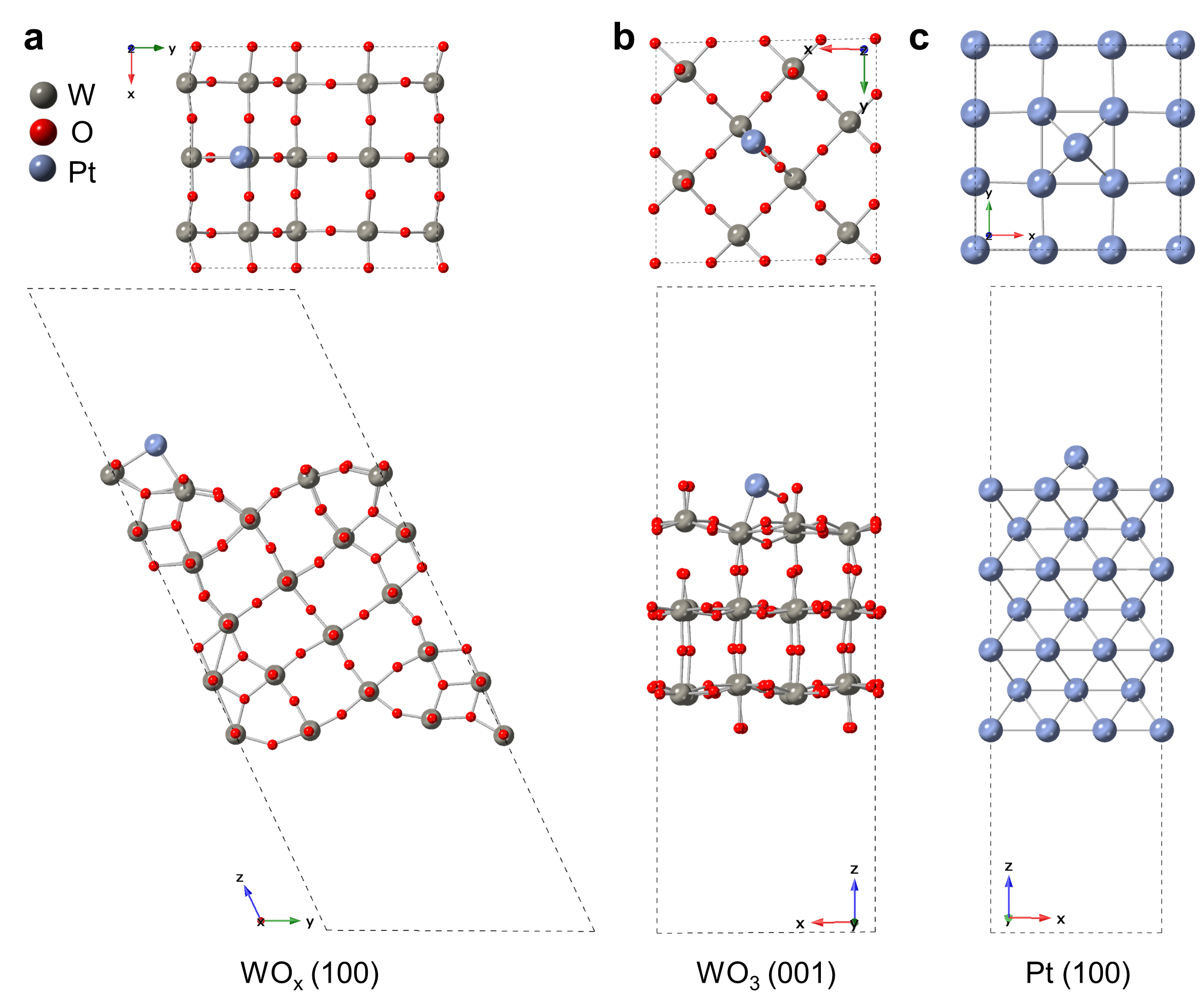


**Figure S31.** Adsorption configurations of a Pt atom on a) WO_x_ (100) b) WO_3_ (001) and c) Pt (100).


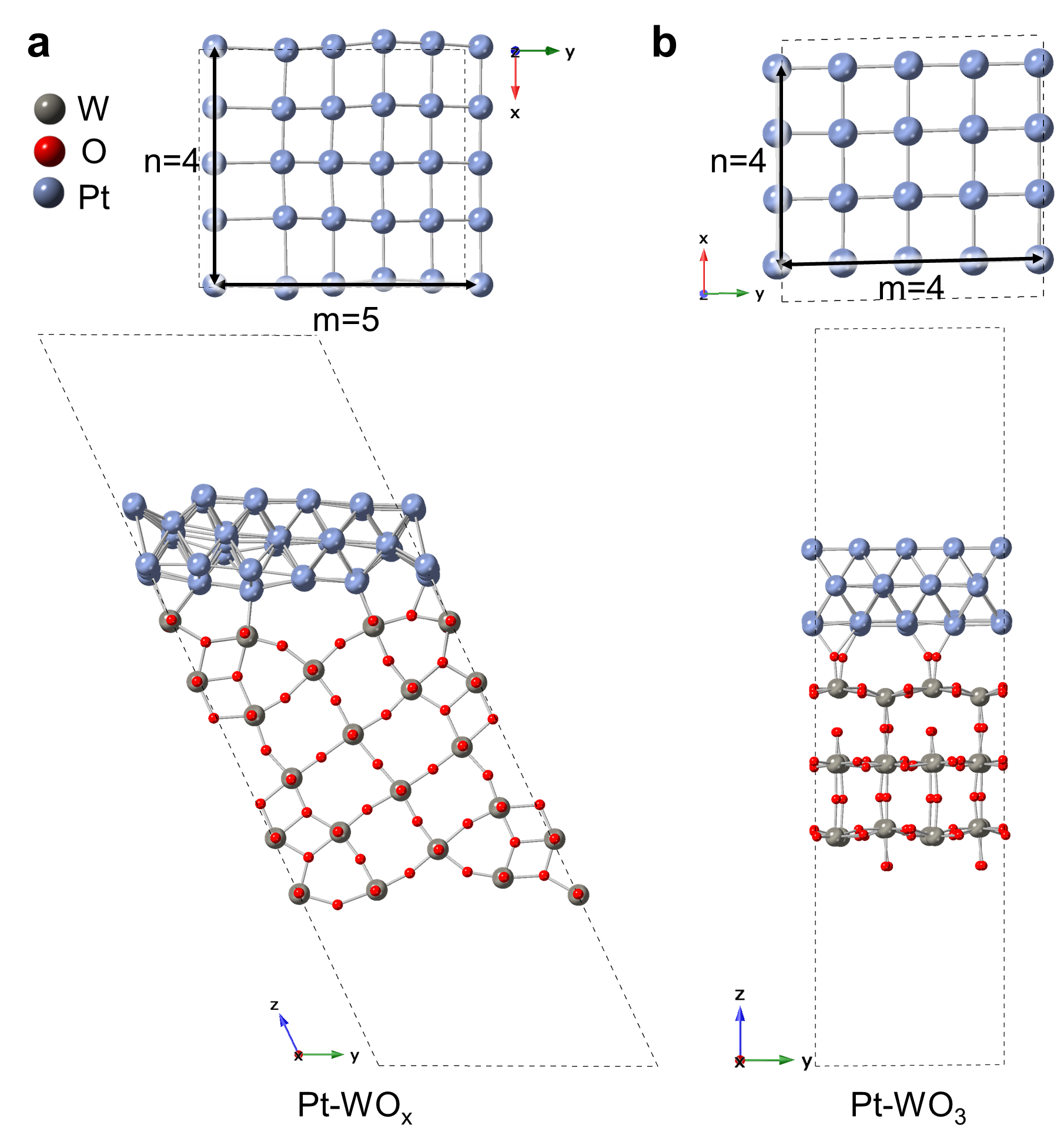


**Figure S32.** Relaxed interface structures of a) Pt–WO_x_ and b) Pt–WO_3_.


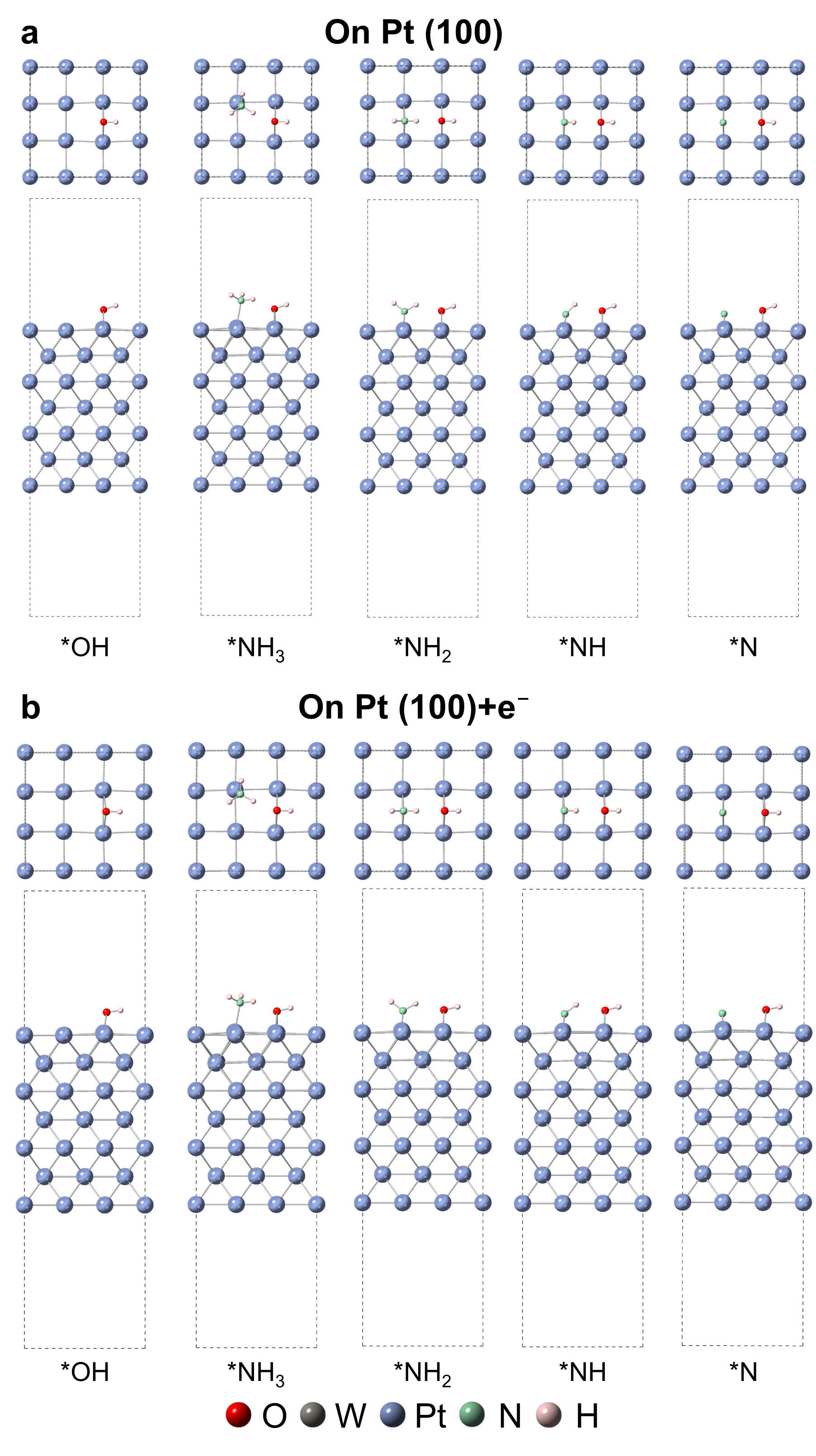


**Figure S33.** Adsorption configurations of *OH, *NH_3_, *NH_2_, *NH, *N on a) Pt (100) and b) Pt (100)+e⁻.


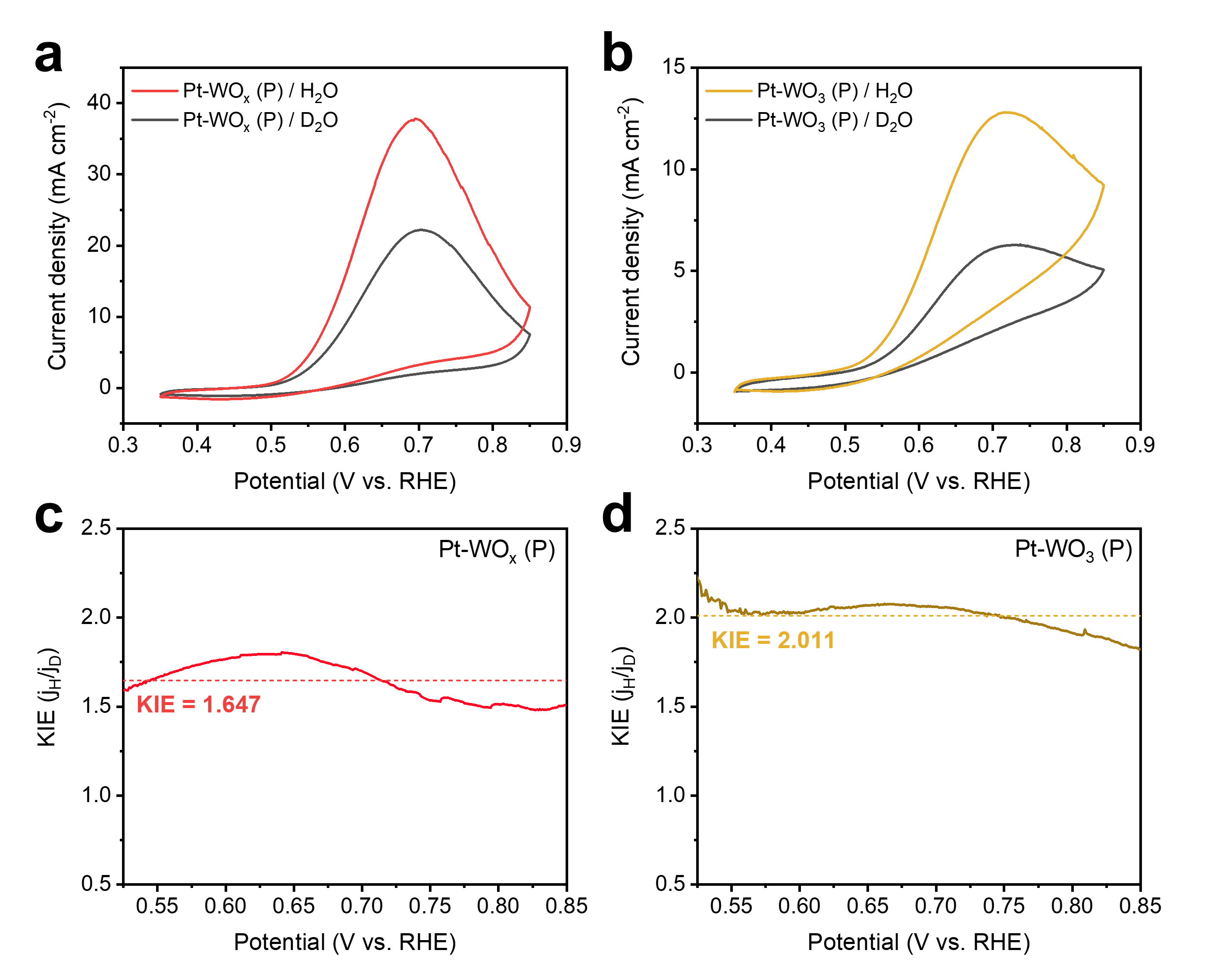


**Figure S34.** Kinetic isotope effect (KIE) measurements. CV curves for a) Pt–WO_x_ (P) and b) Pt–WO_3_ (P) in 0.1 M NH_3_ + 1 M KOH and 0.1 M NH_3_ + 1 M KOD at scan rate 2 mV s^–1^. KIE values as a function of potential (V vs. RHE) for c) Pt–WO_x_ (P) and d) Pt–WO_3_ (P).


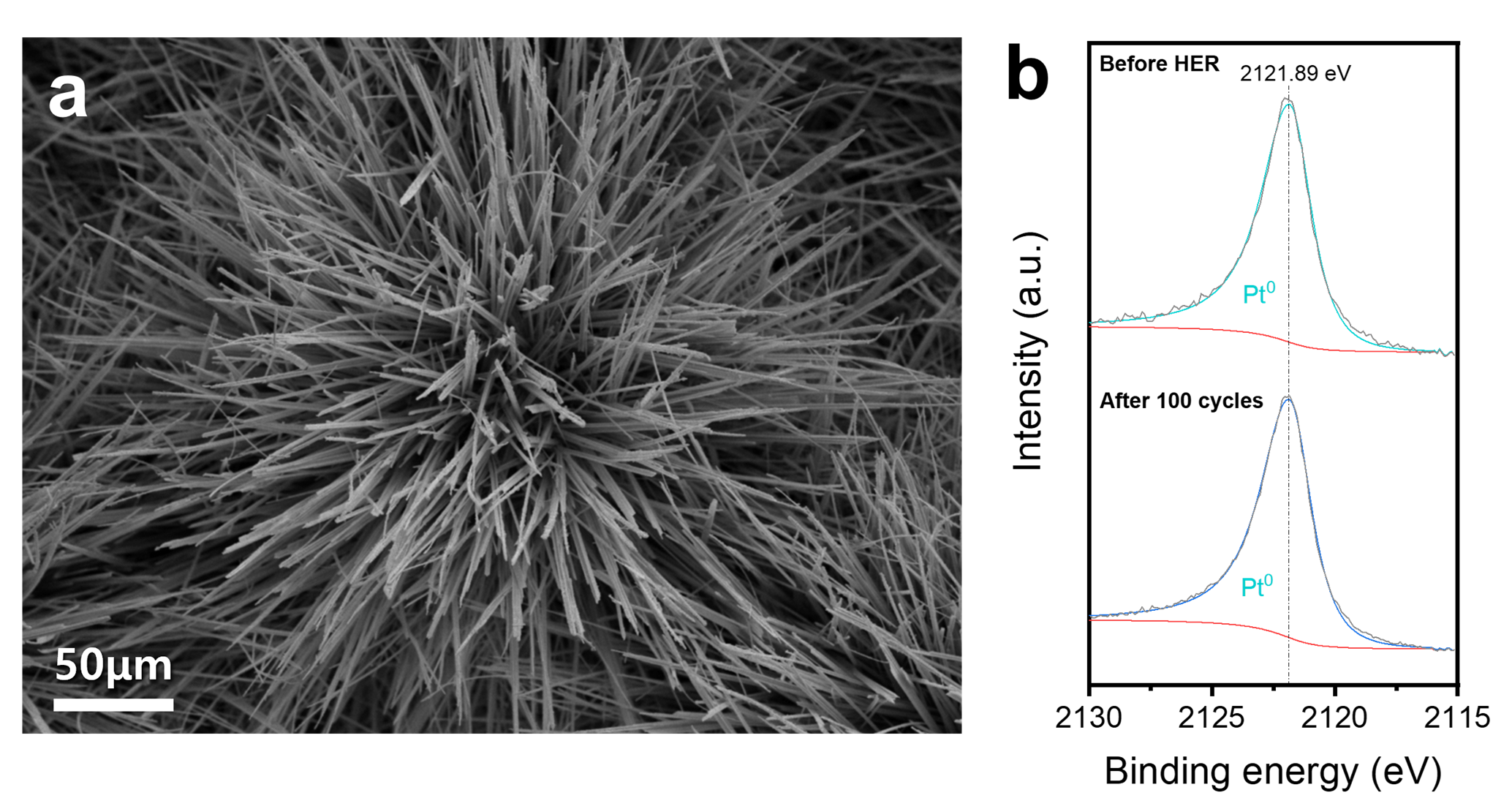


**Figure S35**. a) SEM image of Pt–WO_x_ (P) after 1000 cycles of HER. XPS analysis of Pt 3d orbital before and after 100 cycles of HER with Pt–WO_x_ (P).


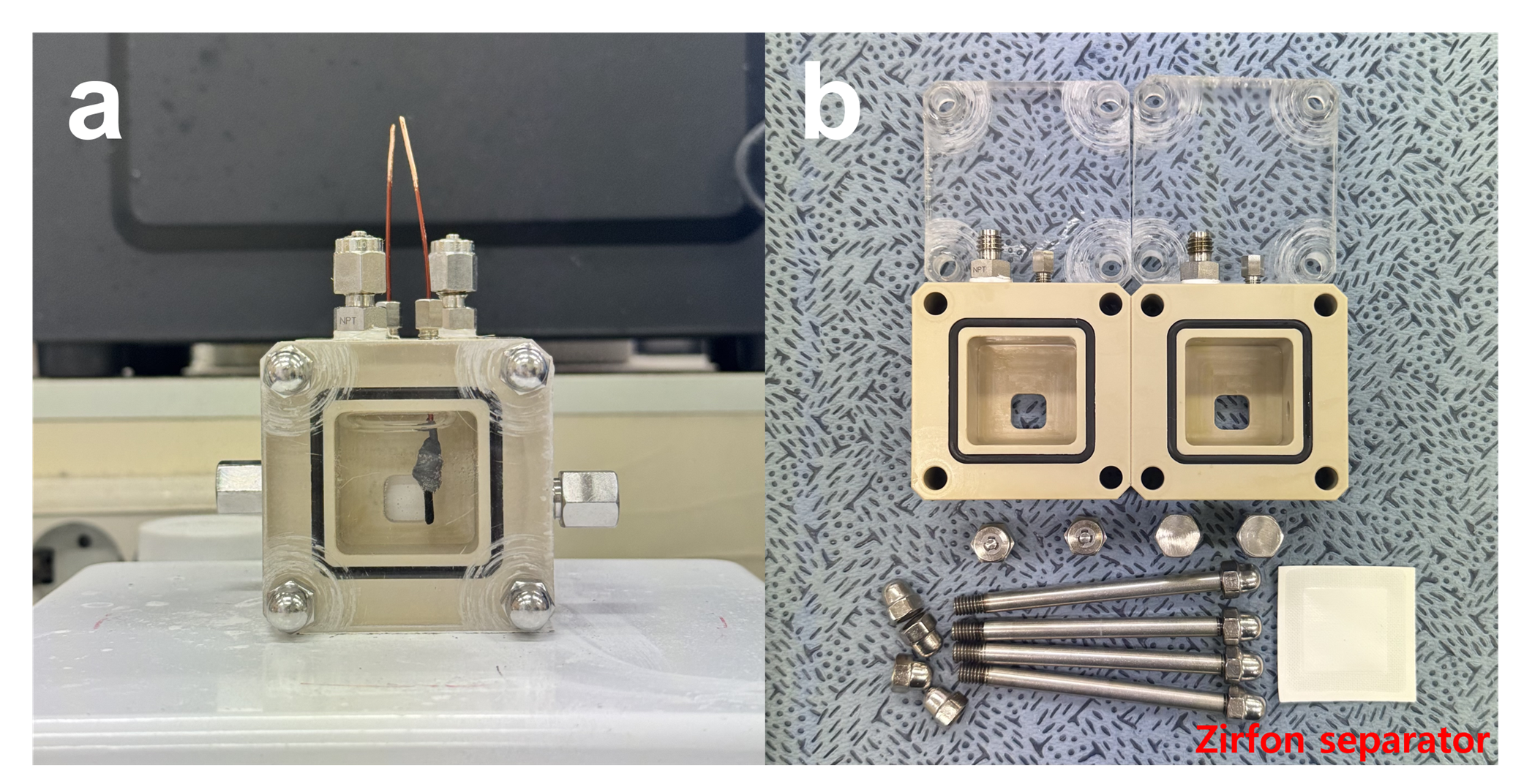


**Figure S36.** Photographs of a) custom H-type cell and b) individual components.


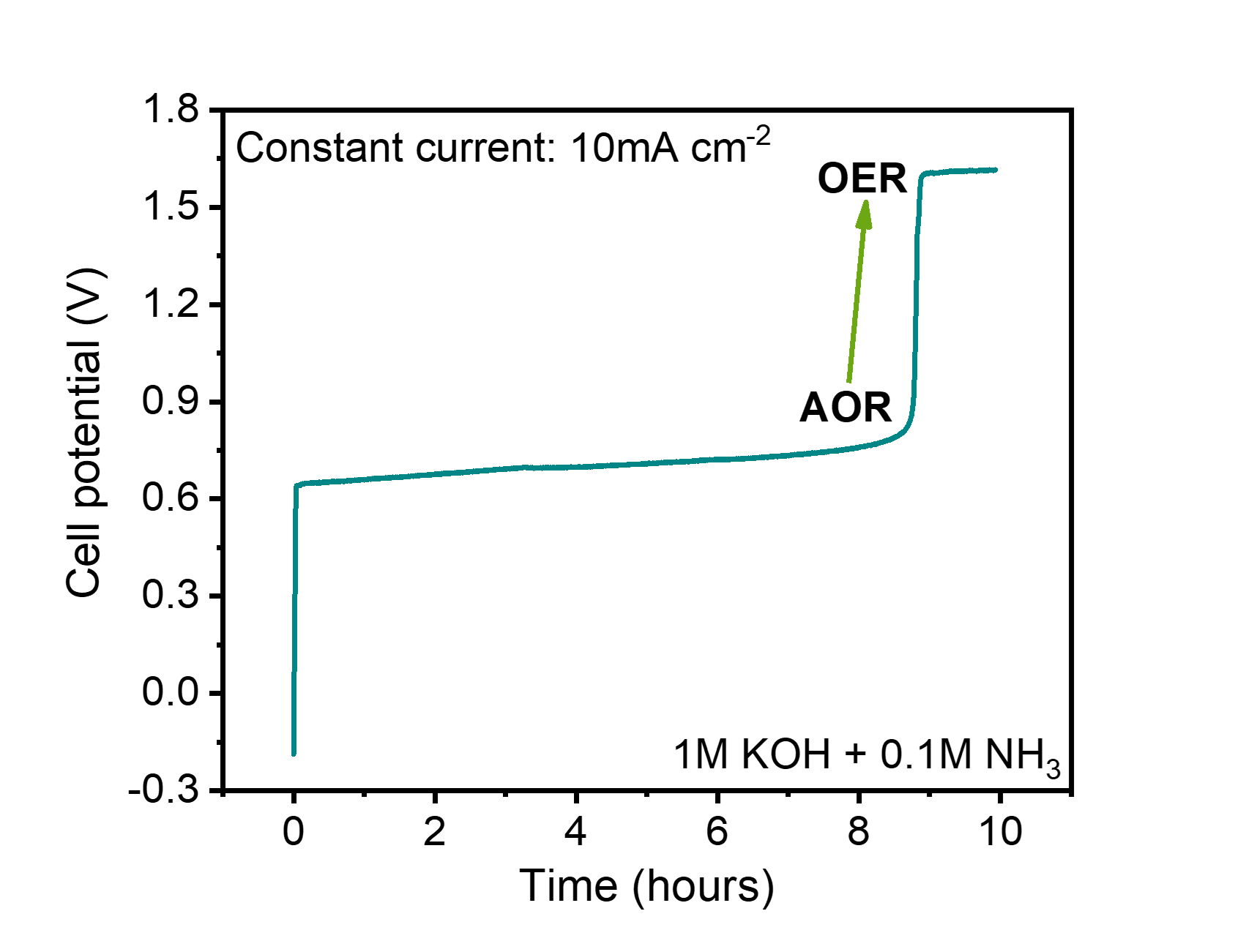


**Figure S37.** CA test at constant current (10 mA cm^−2^) of Pt–WO_x_ (P).

**Figure S38.** CP profiles under pulsed current conditions (10 mA cm^−2^ for 300 s and −10 mA cm^−2^ for 60 s), recorded in a two-electrode H-type cell during a portion of the long-term stability test (10.0 to 10.5 h).


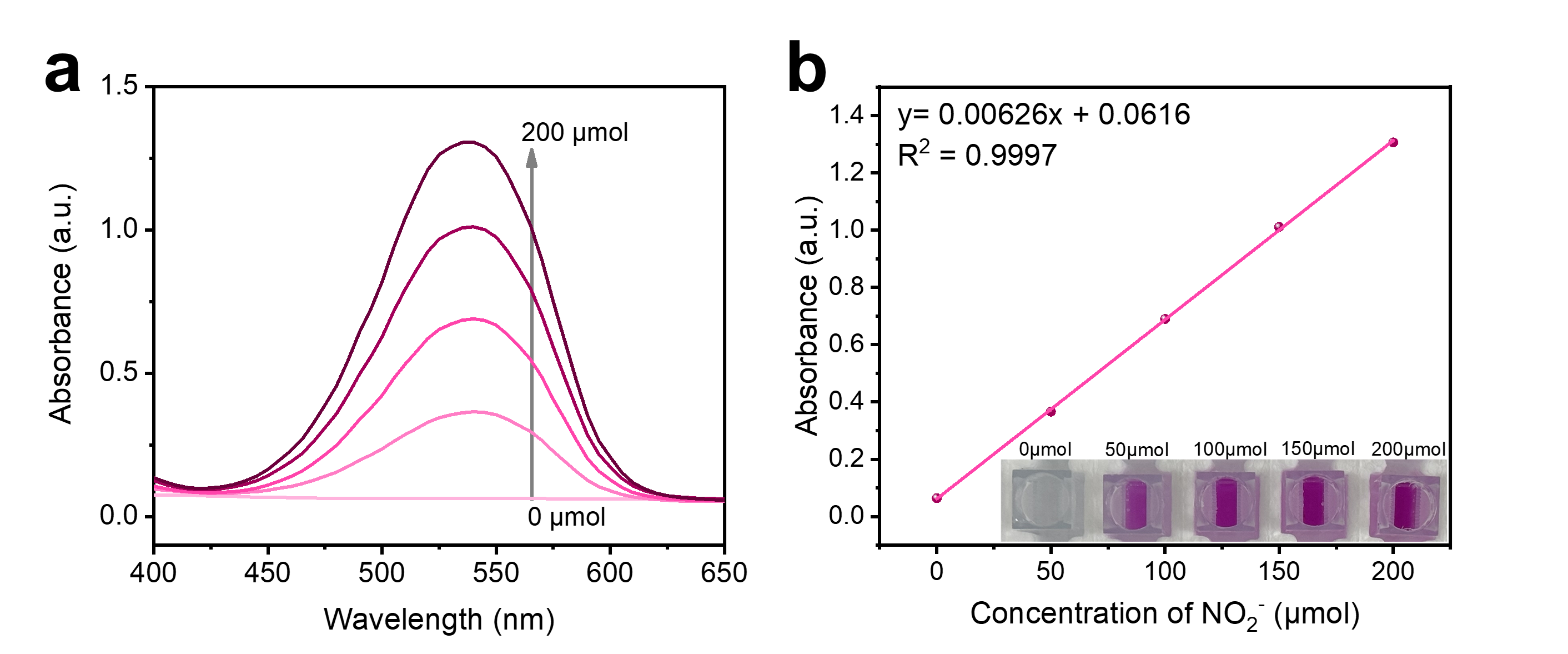


**Figure S39.** a) UV-Vis curves of electrolyte solution with different NO_2_^–^ concentrations. b) Linear fitting of the corresponding calibration curve for the relationship between the absorbance and NO_2_^–^ concentration; Insert: Photograph of solution colored by the Griess colorimetric reagent with increasing NO_2_^–^ concentration.


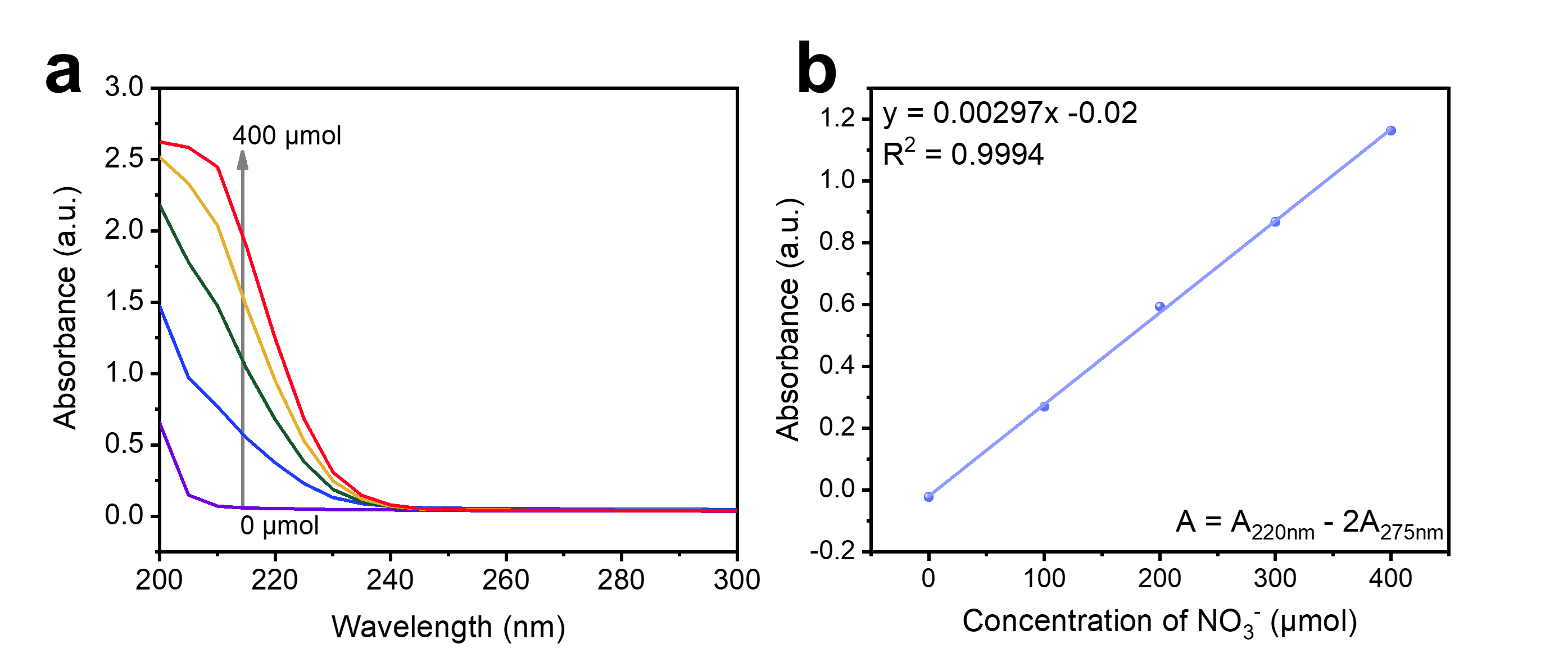


**Figure S40.** a) UV-Vis curves of electrolyte solution with different NO_3_^–^ concentrations. b) Linear fitting of the corresponding calibration curve for the relationship between the absorbance and NO_3_^–^ concentration.


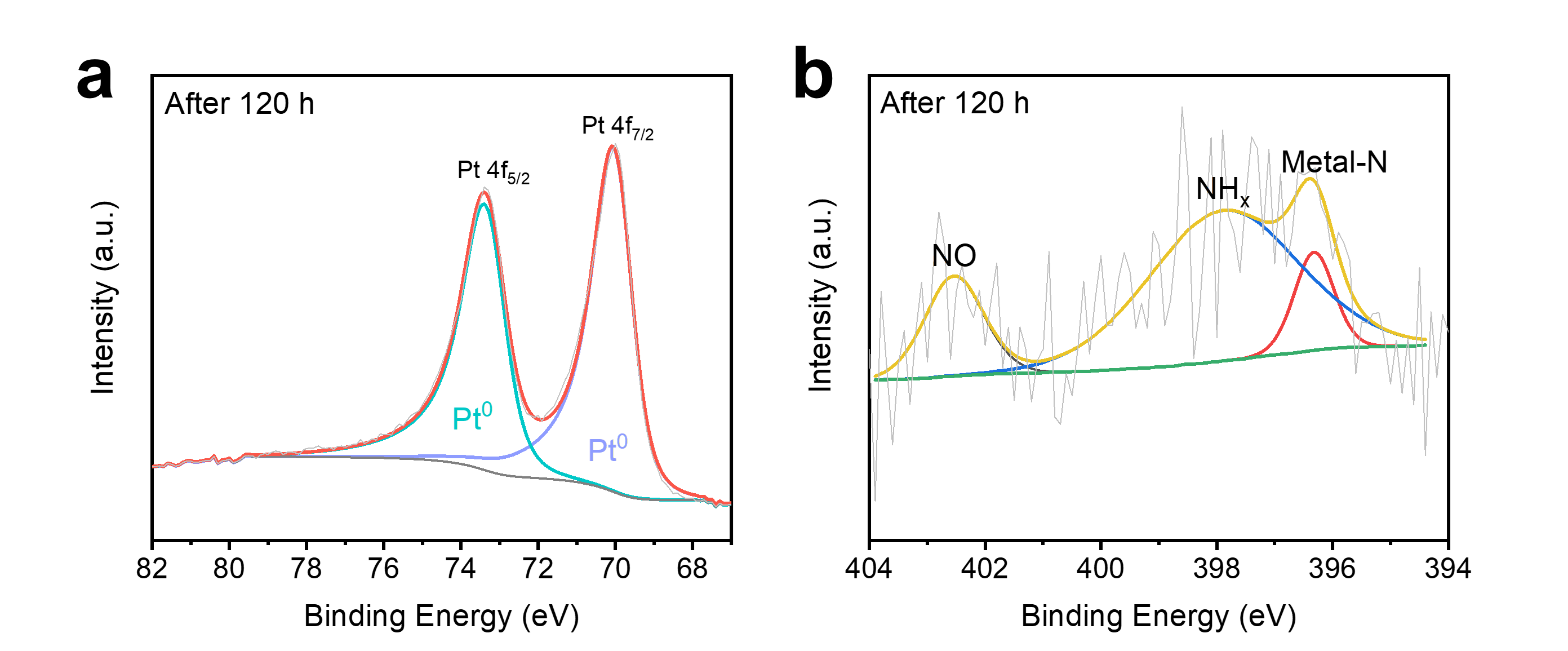


**Figure S41.** XPS spectra of a) Pt 4f and b) N 1s after 120 h ammonia electrolysis using Pt–WO_x_ (P).


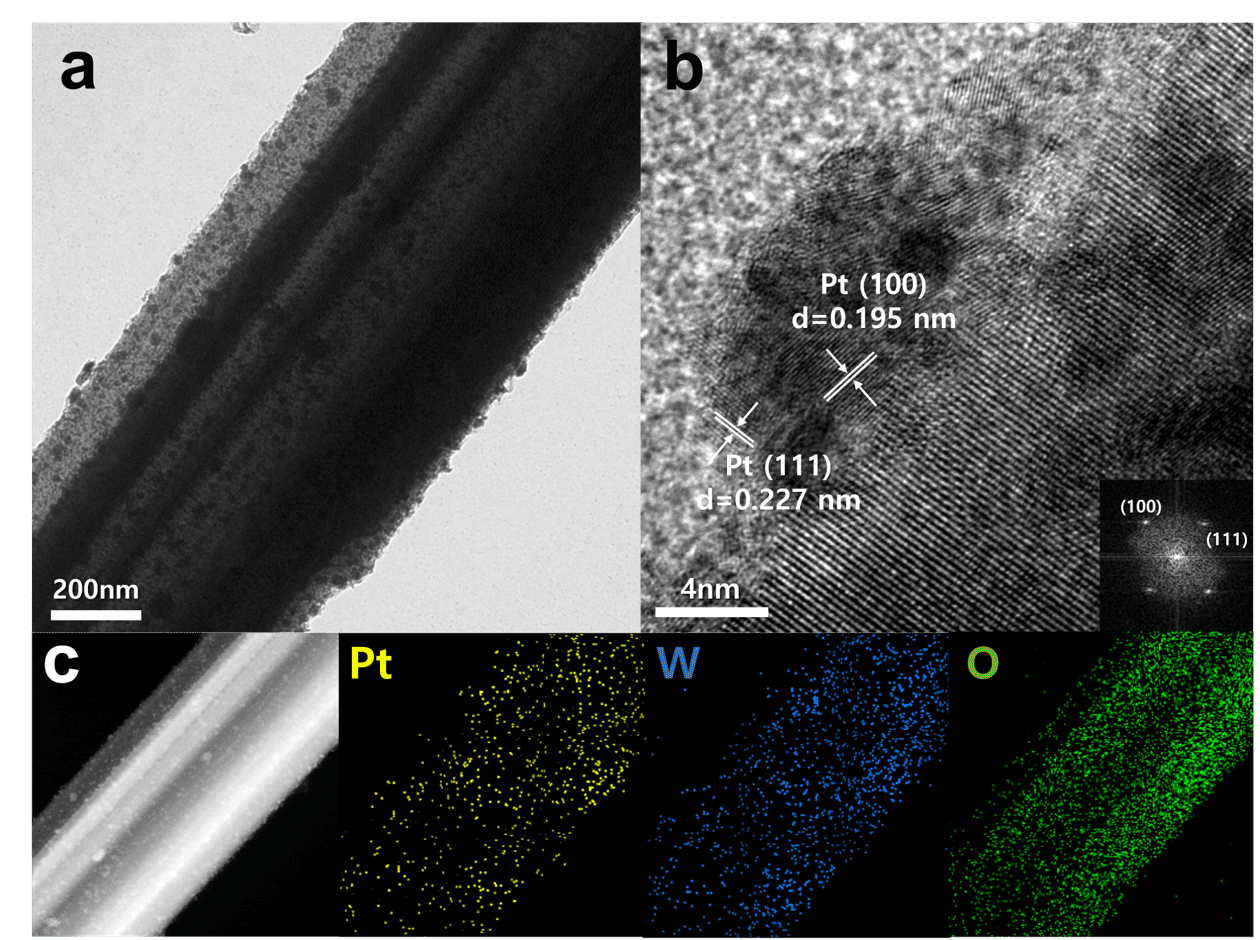


**Figure S42.** a) TEM image of Pt–WO_x_ (P) after 120 h stability test. b) HRTEM image of Pt–WO_x_ (P) after stability test showing the crystallinity of Pt (100) and (111) (insets: corresponding FFT patterns) and c) corresponding EDS elemental mappings (Pt, W, O) of Pt–WO_x_ (P).


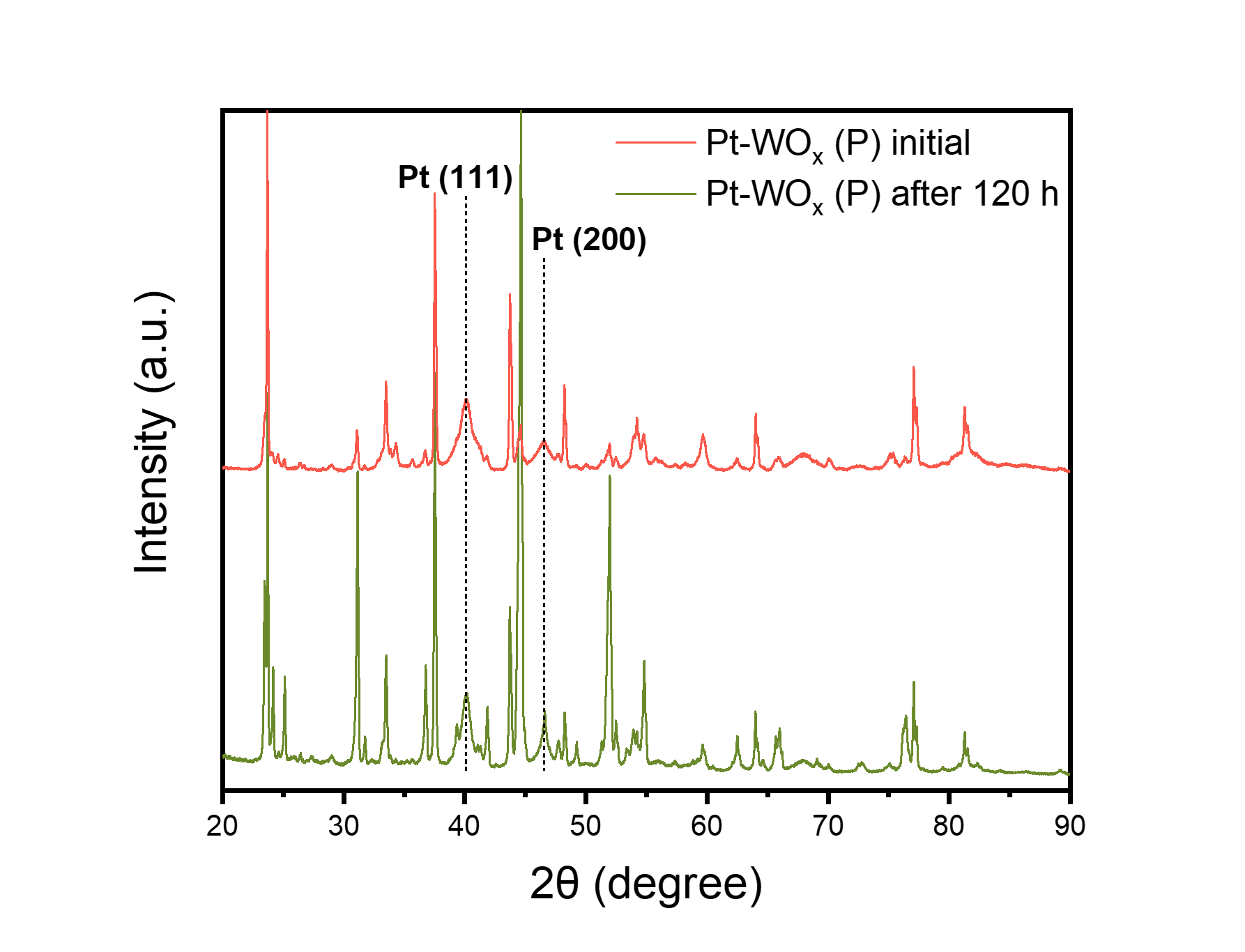


**Figure S43.** XRD patterns Pt–WO_x_ (P) before and after 120 h ammonia electrolysis.

**Table S2.** Integrated intensity (Int. I.) of Pt (111) and Pt (200) through peak deconvolution of XRD results and facet ratio (Pt (200)/Pt (111)) of Pt–WO_x_ (P) before and after 120 h ammonia electrolysis.

| **Pt-WO_x_ (P)** | Int. I. (counts/°)  Pt (111) | Int. I. (counts/°)  Pt (200) | Int. I. ratio  Pt (200)/Pt (111) |
| --- | --- | --- | --- |
| Initial | 8964 ± (518) | 4922 ± (50) | 0.549 |
| After 120 h | 7576 ± (221) | 4644 ± (55) | 0.613 |


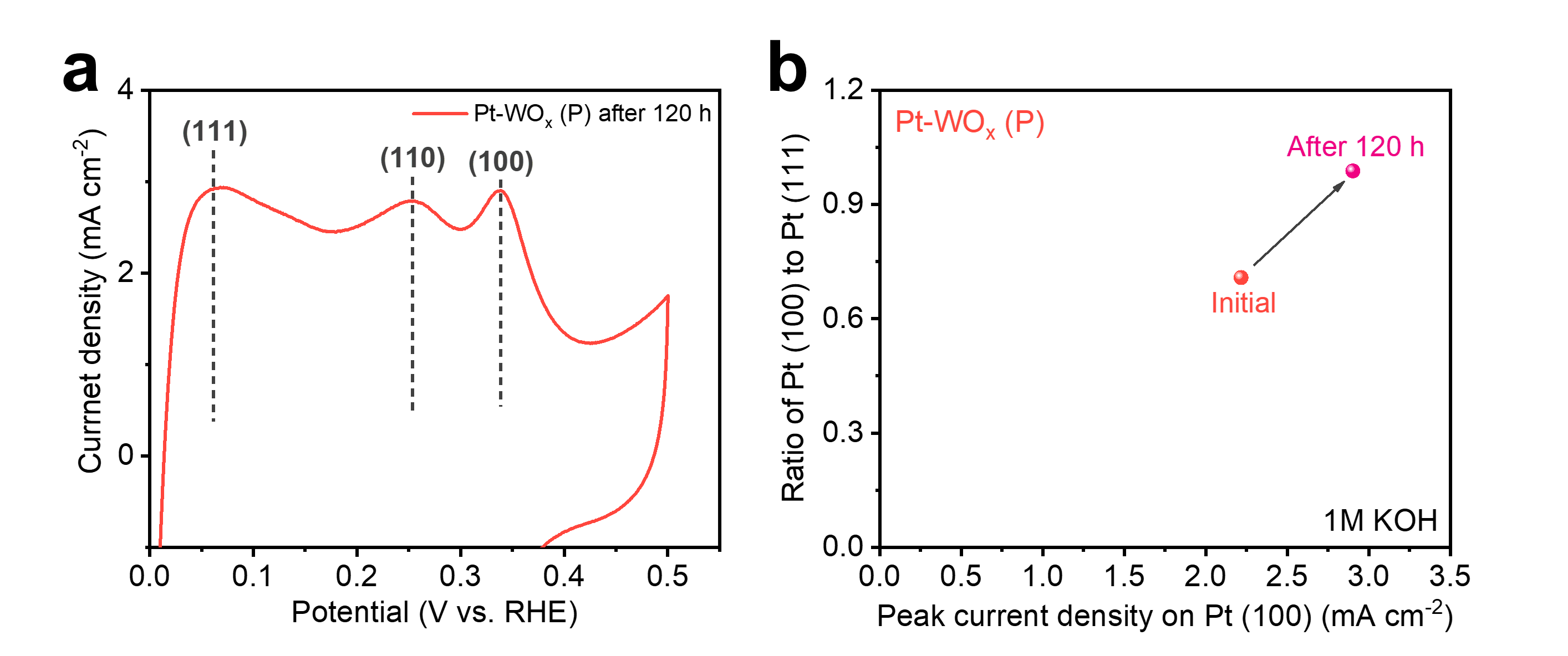


**Figure S44.** a) CV curves of Pt-WO_x_ (P) after 120 h durability test in 1 M KOH between 0 to 0.5 V vs. RHE at scan rate of 20 mV s^–1^. b) Comparison of Pt (100) peak current density and Pt (100)/Pt (111) ratio for Pt–WO_x_ (P) before and after 120 h durability test.


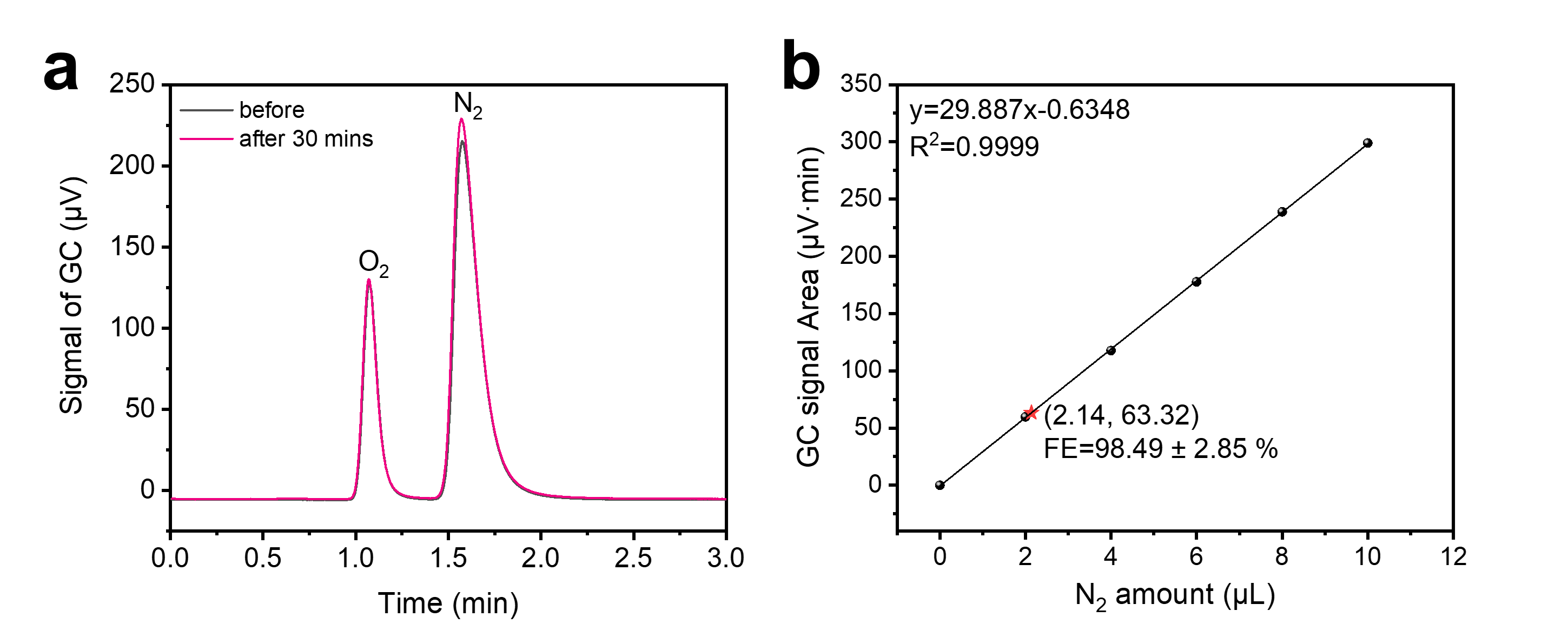


**Figure S45.** a) Gas chromatography (GC) analysis of N₂ and O₂ before and after 30 min of ammonia electrolysis at the anode. b) Calibration curve of N₂ obtained from the signal area versus injected volume, together with the quantified N₂ amount after 30 min of electrolysis and the corresponding Faradaic efficiency.


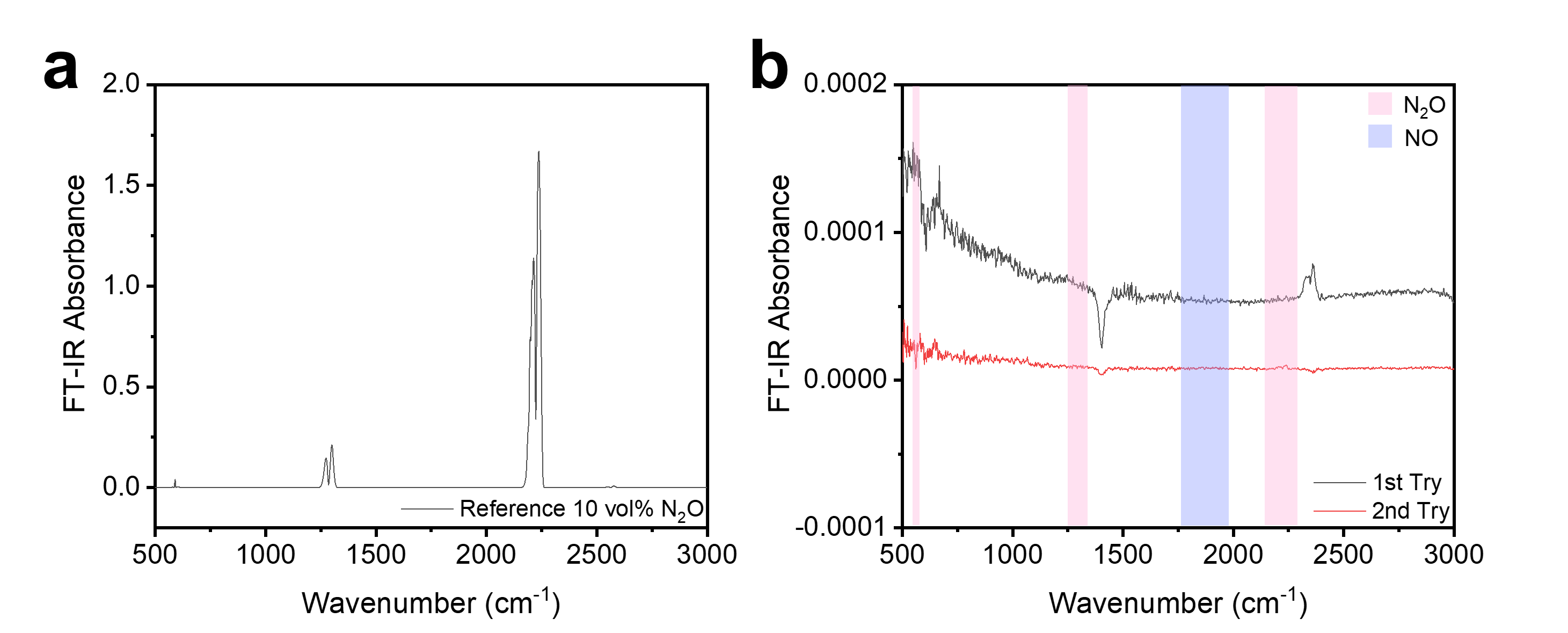


**Figure S46.** a) FT-IR spectrum of reference gas containing 10 vol% N_2_O. b) FT-IR detection of N_2_O and NO peaks in the gas phase after 30 min of ammonia electrolysis.


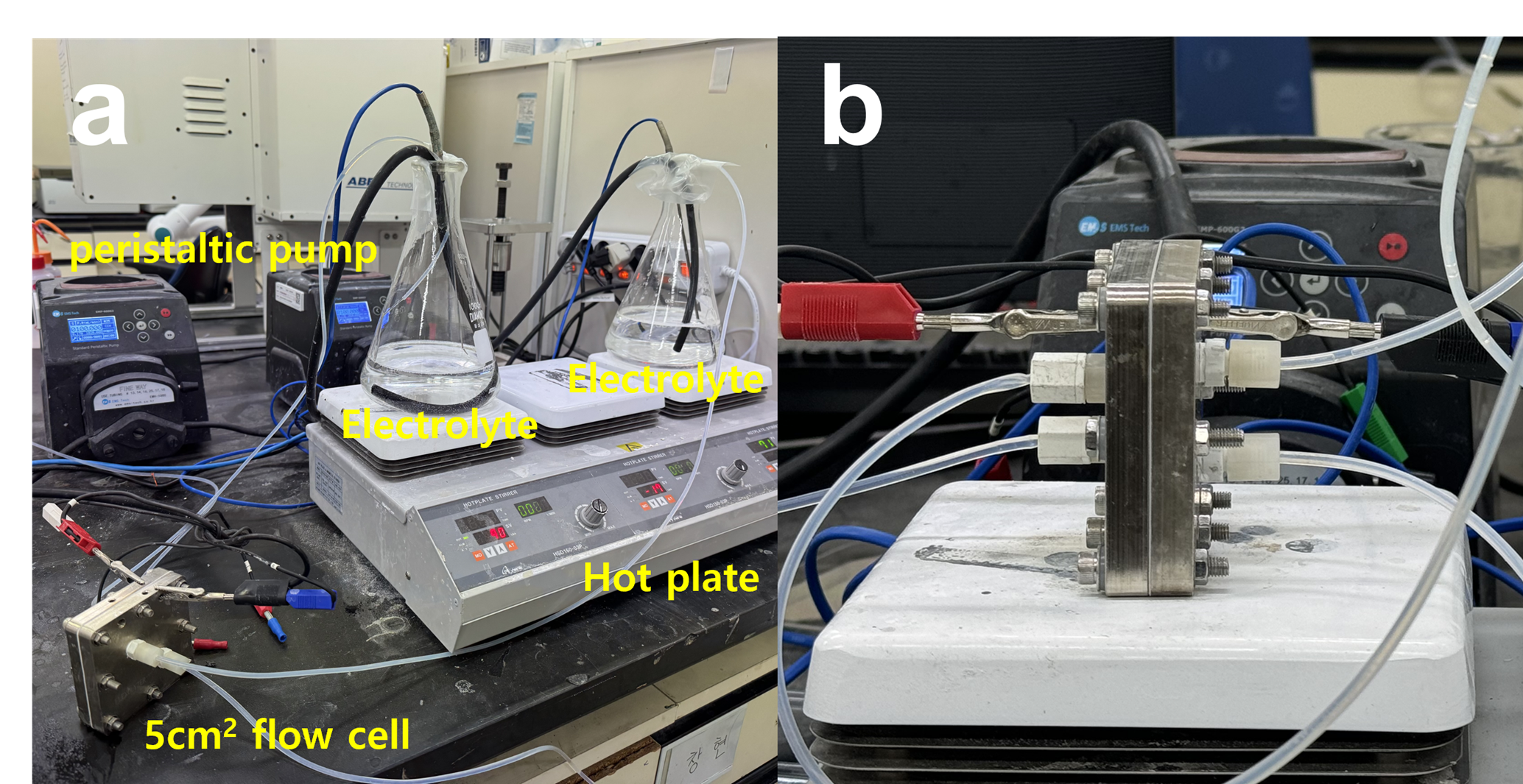


**Figure S47.** Photographs of a) ammonia electrolysis flow system and b) close-up of the 5 cm^2^ flow-type cell.

**Table S3.** Comparison of electrocatalytic AOR performance with previously reported catalysts.

| **Catalyst** | **Electrolyte** | **Scan rate**  **[mV s^−1^]** | **Peak current density**  **[mA cm^−2^]** | **Mass activity**  **[A g^−1^]** | **Onset potential** | **Ref.** |
| --- | --- | --- | --- | --- | --- | --- |
| Pt–WO_x_ (P) | 1M KOH +  0.1M NH_3_ | 5 | 49.67 | 79.97 | 0.46 V vs.  RHE | **This work** |
| Pt–WO_3_ (P) | 1M KOH +  0.1M NH_3_ | 5 | 17.34 | 21.40 | 0.51 V vs.  RHE | **This work** |
| Pt–WO_x_ (S) | 1M KOH +  0.1M NH_3_ | 5 | 6.20 | 18.78 | 0.56 V vs.  RHE | **This work** |
| Ni–Pt_86_Mo_7_ | 1M KOH +  0.1M NH_3_ | 5 | 12.11 | 94.96 | 0.49 V vs.  RHE | [15] |
| Pt_93_Mo_7_ | 1M KOH +  0.1M NH_3_ | 5 | 10.96 | 58.83 | 0.50 V vs.  RHE | [15] |
| Pt_44_W_11_ | 1M KOH +  0.1M NH_3_ | 5 | 11.70 | 57.00 | 0.46 V vs.  RHE | [16] |
| PtIrZn_2_/CeO_2_-ZIF-8 | 1M KOH +  0.1M NH_3_ | 5 | 3.24 | 31.8 | 0.345 V vs.  RHE | [17] |
| C-Pt/SnO2 | 1M KOH +  0.1M NH_3_ | 20 | 1.62 | 57.86 | 0.45 V vs.  RHE | [18] |
| SnO2-Pt/C | 1M KOH +  0.1M NH_3_ | 20 | 1.52 | 54.29 | 0.45 V vs.  RHE | [18] |
| Pt  nanoparticle | 1 MKOH +  0.2M NH_4_OH | 50 | 0.93 | 7.05 | **−**0.48 V vs. Hg/HgO | [19] |
| Pt/Ir/MWCNT nanoparticle | 0.1M KOH +  0.1M NH_3_ | 50 | 0.23 | 57.00 | ~0.36 V vs.  RHE | [20] |

**Table S4.** Calculated Gibbs free energies of H_2_ (g), N_2_ (g) molecules.

| **Molecules** | **E (eV)** | **ZPE (eV)** | **TΔS (eV)** | **G (eV)** |
| --- | --- | --- | --- | --- |
| H_2_ (g) | -6.77 | 0.27 | 0.40 | -6.90 |
| N_2_ (g) | -16.63 | 0.15 | 0.63 | -17.11 |

**Table S5.** Calculated Gibbs free energies of *OH, *NH_3_, *NH_2_, *NH, and *N intermediates adsorbed on Pt (100) and Pt (100)+e⁻.

| **Pt (100)** | | | | | |
| --- | --- | --- | --- | --- | --- |
| **Intermediates** | **E (eV)** | **ZPE (eV)** | **TΔS (eV)** | **G (eV)** | **ΔG (eV) at U=0 V** |
| *OH | -378.00 | 0.40 | 0.37 | -377.97 | 0.00 |
| *NH_3_ | -399.11 | 1.46 | 0.65 | -398.29 | -1.41 |
| *NH_2_ | -395.24 | 1.18 | 0.70 | -394.76 | -1.34 |
| *NH | -390.39 | 0.82 | 0.70 | -390.27 | -0.30 |
| *N | -386.15 | 0.53 | 0.69 | -386.32 | 0.21 |

| **Pt (100)+e⁻** | | | | | |
| --- | --- | --- | --- | --- | --- |
| **Intermediates** | **E (eV)** | **ZPE (eV)** | **TΔS (eV)** | **G (eV)** | **ΔG (eV) at U=0 V** |
| *OH | -376.42 | 0.40 | 0.37 | -376.39 | 0.00 |
| *NH_3_ | -396.74 | 1.45 | 0.64 | -395.93 | -0.64 |
| *NH_2_ | -393.04 | 1.17 | 0.71 | -392.59 | -0.74 |
| *NH | -388.29 | 0.81 | 0.70 | -388.18 | 0.21 |
| *N | -384.17 | 0.52 | 0.70 | -384.34 | 0.60 |

**References**

[1] G. Kresse, J. Furthmüller, *Phys. Rev. B* **1996**, *54*, 11169.

[2] P. E. Blöchl, *Phys. Rev. B* **1994**, *50*, 17953.

[3] J. P. Perdew, K. Burke, M. Ernzerhof, *Phys. Rev. Lett.* **1996**, *77*, 3865.

[4] S. Grimme, *J. Comput. Chem.* **2004**, *25*, 1463.

[5] K. Viswanathan, K. Brandt, E. Salje, *J. Solid State Chem.* **1981**, *36*, 45.

[6] H. M. Le, N. H. Vu, B.-T. Phan, *Comput. Mater. Sci.* **2014**, *90*, 171.

[7] J. W. Arblaster, *Selected values of the crystallographic properties of elements*, **2018**.

[8] H. Zhang, Y. Wang, S. Zuo, W. Zhou, J. Zhang, X. W. D. Lou, *J. Am. Chem. Soc.* **2021**, *143*, 2173.

[9] F. Wang, C. Di Valentin, G. Pacchioni, *J. Phys. Chem. C* **2012**, *116*, 10672.

[10] O. Hurtado-Aular, R. Añez, A. Sierraalta, J. Calderón, *Appl. Surf. Sci.* **2020**, *506*, 144719.

[11] I. N. Yakovkin, M. Gutowski, *Surf. Sci.* **2007**, *601*, 1481.

[12] C. Lim, H. Roh, E. H. Kim, H. Kim, T. Park, D. Lee, K. Yong, *Small* **2023**, *19*, 2304274.

[13] J. K. Nørskov, J. Rossmeisl, A. Logadottir, L. Lindqvist, J. R. Kitchin, T. Bligaard, H. Jónsson, *J. Phys. Chem. B* **2004**, *108*, 17886.

[14] G. Henkelman, A. Arnaldsson, H. Jónsson, *Comput. Mater. Sci* **2006**, *36*, 354.

[15] S. Liu, Y. Jiang, M. Wang, Y. Huan, Y. He, Q. Cheng, Y. Cheng, J. Liu, X. Zhou, T. Qian, C. Yan, *Adv. Funct. Mater.* **2023**, *33*, 2306204.

[16] Y. Huan, Y. Jiang, M. Wang, X. Zhou, X. Shen, Y. Cao, C. Yan, T. Qian, *Chem. Eng. J.* **2023**, *475*, 146027.

[17] Y. Li, H. S. Pillai, T. Wang, S. Hwang, Y. Zhao, Z. Qiao, Q. Mu, S. Karakalos, M. Chen, J. Yang, D. Su, H. Xin, Y. Yan, G. Wu, *Energy Environ. Sci.* **2021**, *14*, 1449.

[18] T. Okanishi, Y. Katayama, H. Muroyama, T. Matsui, K. Eguchi, *Electrochim. Acta* **2015**, *173*, 364.

[19] S. Ntais, A. Serov, N. I. Andersen, A. J. Roy, E. Cossar, A. Allagui, Z. Lu, X. Cui, E. A. Baranova, P. Atanassov, *Electrochim. Acta* **2016**, *222*, 1455.

[20] B. K. Boggs, G. G. Botte, *Electrochim. Acta* **2010**, *55*, 5287.
